# Supplementary material for: Structures and Bioactivities of Quadrangularisosides A, A1, B, B1, B2, C, C1, D, D1–D4, and E from the Sea Cucumber Colochirus quadrangularis: The First Discovery of the Glycosides, Sulfated by C-4 of the Terminal 3-O-Methylglucose Residue. Synergetic Effect on Colony Formation of Tumor HT-29 Cells of these Glycosides with Radioactive Irradiation
Source: Mar Drugs. 2020 Jul 28;18(8):394. doi: 10.3390/md18080394 (PMC7460491; doi:10.3390/md18080394)
Supplement: Supplementary file 1 [file marinedrugs-18-00394-s001.pdf]

Supplementary data content page

Title: Structures and bioactivities of quadrangularisosides A, A_1, B, B_1, B_2, C, C_1, D, D_1–D_4, and E from the sea cucumber *Colochirus quadrangularis*. The first discovery of the glycosides, sulfated by C-4 of the terminal 3-*O*-methylglucose residue. Synergetic effect on colony formation of tumor HT-29 cells of these glycosides with radioactive irradiation

Authors: Alexandra S. Silchenko*, Anatoly I. Kalinovsky, Sergey A. Avilov, Vladimir I. Kalinin, Pelageya V. Andrijaschenko, Roman S. Popov, Pavel. S Dmitrenok, Ekaterina A. Chingizova, Svetlana P. Ermakova, Olesya S. Malyarenko, Salim Sh. Dautov

Address: [1]*G.B. Elyakov Pacific Institute of Bioorganic Chemistry, Far Eastern Branch of Russian Academy of Sciences, Pr. 100-let Vladivostoku 159, 690022 Vladivostok, Russia*

2A.V. Zhirmunsky National Scientific Center of Marine Biology, Far Eastern Branch, Russian Academy of Sciences, 17 Palchevskogo Street, Vladivostok, 690041, Russia

Correspondence: sialexandra@mail.ru; Tel.: +7-423-231-1168

Contents:

Fig. 1. The ^{13}C NMR (176.03 MHz) spectrum of quadrangularisoside A (**1**) in C_5D_5N/D_2O (4/1)

Fig. 2. The ^{1}H NMR (700.00 MHz) spectrum of quadrangularisoside A (**1**) in C_5D_5N/D_2O (4/1)

Fig. 3. The COSY (700.00 MHz) spectrum of quadrangularisoside A (**1**) in C_5D_5N/D_2O (4/1)

Fig. 4. The HSQC (700.00 MHz) spectrum of quadrangularisoside A (**1**) in C_5D_5N/D_2O (4/1)

Fig. 5. The HMBC (700.00 MHz) spectrum of quadrangularisoside A (**1**) in C_5D_5N/D_2O (4/1)

Fig. 6. The ROESY (700.00 MHz) spectrum of quadrangularisoside A (**1**) in C_5D_5N/D_2O (4/1)

Fig. 7. 1 D TOCSY (700.00 MHz) spectra of quadrangularisoside A (**1**) in C_5D_5N/D_2O (4/1)

Fig. 8. 1 D TOCSY (700.00 MHz) spectra of quadrangularisoside A (**1**) in C_5D_5N/D_2O (4/1)

Fig. 9. The ^{13}C NMR (176.03 MHz) spectrum of quadrangularisoside A_1 (**2**) in C_5D_5N/D_2O (4/1)

Fig. 10. The ^{1}H NMR (700.00 MHz) spectrum of quadrangularisoside A_1 (**2**) in C_5D_5N/D_2O (4/1)

Fig. 11. The COSY (700.00 MHz) spectrum of quadrangularisoside A_1 (**2**) in C_5D_5N/D_2O (4/1)

Fig. 12. The HSQC (700.00 MHz) spectrum of quadrangularisoside A_1 (**2**) in C_5D_5N/D_2O (4/1)

Fig. 13. The ROESY (700.00 MHz) spectrum of quadrangularisoside A_1 (**2**) in C_5D_5N/D_2O (4/1)

Fig. 14. The HMBC (700.00 MHz) spectrum of quadrangularisoside A_1 (**2**) in C_5D_5N/D_2O (4/1)

Fig. 15. The ^{13}C NMR (176.03 MHz) spectrum of quadrangularisoside B (**3**) in C_5D_5N/D_2O (4/1)

Fig. 16. The ^{1}H NMR (700.00 MHz) spectrum of quadrangularisoside B (**3**) in C_5D_5N/D_2O (4/1)

Fig. 17. The COSY (700.00 MHz) spectrum of quadrangularisoside B (**3**) in C_5D_5N/D_2O (4/1)

Fig. 18. The HSQC (700.00 MHz) spectrum of quadrangularisoside B (**3**) in C_5D_5N/D_2O (4/1)

Fig. 19. The HMBC (700.00 MHz) spectrum of quadrangularisoside B (**3**) in C_5D_5N/D_2O (4/1)

Fig. 20. The ROESY (700.00 MHz) spectrum of quadrangularisoside B (**3**) in C_5D_5N/D_2O (4/1)

Fig. 21. 1D TOCSY (700.00 MHz) spectra of quadrangularisoside B (**3**) in C_5D_5N/D_2O (4/1)

Fig. 22. 1D TOCSY (700.00 MHz) spectra of quadrangularisoside B (**3**) in C_5D_5N/D_2O (4/1)

Fig. 23. The ^{13}C NMR (176.03 MHz) spectrum of quadrangularisoside B_1 (**4**) in C_5D_5N/D_2O (4/1)

Fig. 24. The ^{1}H NMR (700.00 MHz) spectrum of quadrangularisoside B_1 (**4**) in C_5D_5N/D_2O (4/1)

Fig. 25. HR-ESI-MS and ESI-MS/MS spectra of quadrangularisoside B_1 (**4**)

Fig. 26. The ^{13}C NMR (176.03 MHz) spectrum of quadrangularisoside B_2 (**5**) in C_5D_5N/D_2O (4/1)

Fig. 27. The ^{1}H NMR (700.00 MHz) spectrum of quadrangularisoside B_2 (**5**) in C_5D_5N/D_2O (4/1)

Fig. 28. The COSY (700.00 MHz) spectrum of quadrangularisoside B_2 (**5**) in C_5D_5N/D_2O (4/1)

Fig. 29. The HSQC (700.00 MHz) spectrum of quadrangularisoside B_2 (**5**) in C_5D_5N/D_2O (4/1)

Fig. 30. The ROESY (700.00 MHz) spectrum of quadrangularisoside B_2 (**5**) in C_5D_5N/D_2O (4/1)

Fig. 31. The HMBC (700.00 MHz) spectrum of quadrangularisoside B_2 (**5**) in C_5D_5N/D_2O (4/1)

Fig. 32. The ^{13}C NMR (176.03 MHz) spectrum of quadrangularisoside C (**6**) in C_5D_5N/D_2O (4/1)

Fig. 33. The ^{1}H NMR (700.00 MHz) spectrum of quadrangularisoside C (**6**) in C_5D_5N/D_2O (4/1)

Fig. 34. The COSY (700.00 MHz) spectrum of quadrangularisoside C (**6**) in C_5D_5N/D_2O (4/1)

Fig. 35. The HSQC (700.00 MHz) spectrum of quadrangularisoside C (**6**) in C_5D_5N/D_2O (4/1)

Fig. 36. The HMBC (700.00 MHz) spectrum of quadrangularisoside C (**6**) in C_5D_5N/D_2O (4/1)

Fig. 37. The ROESY (700.00 MHz) spectrum of quadrangularisoside C (**6**) in C_5D_5N/D_2O (4/1)

Fig. 38. 1D TOCSY (700.00 MHz) spectra of quadrangularisoside C (**6**) in C_5D_5N/D_2O (4/1)

Fig. 39. 1D TOCSY (700.00 MHz) spectra of quadrangularisoside C (**6**) in C_5D_5N/D_2O (4/1)

Fig. 40. HR-ESI-MS and ESI-MS/MS spectra of quadrangularisoside C (**6**)

Fig. 41. The ^{13}C NMR (176.03 MHz) spectrum of quadrangularisoside C_1 (**7**) in C_5D_5N/D_2O (4/1)

Fig. 42. The ^{1}H NMR (700.00 MHz) spectrum of quadrangularisoside C_1 (**7**) in C_5D_5N/D_2O (4/1)

Fig. 43. The COSY (700.00 MHz) spectrum of the aglycone part of quadrangularisoside C_1 (**7**) in C_5D_5N/D_2O (4/1)

Fig. 44. The HSQC (700.00 MHz) spectrum of the aglycone part of quadrangularisoside C_1 (**7**) in C_5D_5N/D_2O (4/1)

Fig. 45. The ROESY (700.00 MHz) spectrum of the aglycone part of quadrangularisoside C_1 (**7**) in C_5D_5N/D_2O (4/1)

Fig. 46. HR-ESI-MS and ESI-MS/MS spectra of quadrangularisoside C_1 (**7**)

Fig. 47. The ^{13}C NMR (176.03 MHz) spectrum of quadrangularisoside D (**8**) in C_5D_5N/D_2O (4/1)

Fig. 48. The ^{1}H NMR (700.00 MHz) spectrum of quadrangularisoside D (**8**) in C_5D_5N/D_2O (4/1)

Fig. 49. The COSY (700.00 MHz) spectrum of quadrangularisoside D (**8**) in C_5D_5N/D_2O (4/1)

Fig. 50. The HSQC (700.00 MHz) spectrum of quadrangularisoside D (**8**) in C_5D_5N/D_2O (4/1)

Fig. 51. The HMBC (700.00 MHz) spectrum of quadrangularisoside D (**8**) in C_5D_5N/D_2O (4/1)

Fig. 52. The ROESY (700.00 MHz) spectrum of quadrangularisoside D (**8**) in C_5D_5N/D_2O (4/1)

Fig. 53. 1D TOCSY (700.00 MHz) spectra of quadrangularisoside D (**8**) in C_5D_5N/D_2O (4/1)

Fig. 54. 1D TOCSY (700.00 MHz) spectra of quadrangularisoside D (**8**) in C_5D_5N/D_2O (4/1)

Fig. 55. The ^{13}C NMR (176.03 MHz) spectrum of quadrangularisoside D_1 (**9**) in C_5D_5N/D_2O (4/1)

Fig. 56. The ^{1}H NMR (700.00 MHz) spectrum of quadrangularisoside D_1 (**9**) in C_5D_5N/D_2O (4/1)

Fig. 57. HR-ESI-MS and ESI-MS/MS spectra of quadrangularisoside D_1 (**9**)

Fig. 58. The ^{13}C NMR (176.03 MHz) spectrum of quadrangularisoside D_2 (**10**) in C_5D_5N/D_2O (4/1)

Fig. 59. The ^{1}H NMR (700.00 MHz) spectrum of quadrangularisoside D_2 (**10**) in C_5D_5N/D_2O (4/1)

Fig. 60. HR-ESI-MS and ESI-MS/MS spectra of quadrangularisoside D_2 (**10**)

Fig. 61. The ^{13}C NMR (176.03 MHz) spectrum of quadrangularisoside D_3 (**11**) in C_5D_5N/D_2O (4/1)

Fig. 62. The ^{1}H NMR (700.00 MHz) spectrum of quadrangularisoside D_3 (**11**) in C_5D_5N/D_2O (4/1)

Fig. 63. HR-ESI-MS and ESI-MS/MS spectra of quadrangularisoside D_3 (**11**)

Fig. 64. The ^{13}C NMR (176.03 MHz) spectrum of quadrangularisoside D_4 (**12**) in C_5D_5N/D_2O (4/1)

Fig. 65. The ^1H NMR (700.00 MHz) spectrum of quadrangularisoside D_4 (**12**) in C_5D_5N/D_2O (4/1)

Fig. 66. HR-ESI-MS and ESI-MS/MS spectra of quadrangularisoside D_4 (**12**)

Fig. 67. The ^{13}C NMR (176.03 MHz) spectrum of quadrangularisoside E (**13**) in C_5D_5N/D_2O (4/1)

Fig. 68. The ^1H NMR (700.00 MHz) spectrum of quadrangularisoside E (**13**) in C_5D_5N/D_2O (4/1)

Fig. 69. The COSY (700.00 MHz) spectrum of quadrangularisoside E (**13**) in C_5D_5N/D_2O (4/1)

Fig. 70. The HSQC (700.00 MHz) spectrum of quadrangularisoside E (**13**) in C_5D_5N/D_2O (4/1)

Fig. 71. The HMBC (700.00 MHz) spectrum of quadrangularisoside E (**13**) in C_5D_5N/D_2O (4/1)

Fig. 72. The ROESY (700.00 MHz) spectrum of quadrangularisoside E (**13**) in C_5D_5N/D_2O (4/1)

Fig. 73. 1D TOCSY (700.00 MHz) spectra of quadrangularisoside E (**13**) in C_5D_5N/D_2O (4/1)

Fig. 74. 1D TOCSY (700.00 MHz) spectra of quadrangularisoside E (**14**) in C_5D_5N/D_2O (4/1)

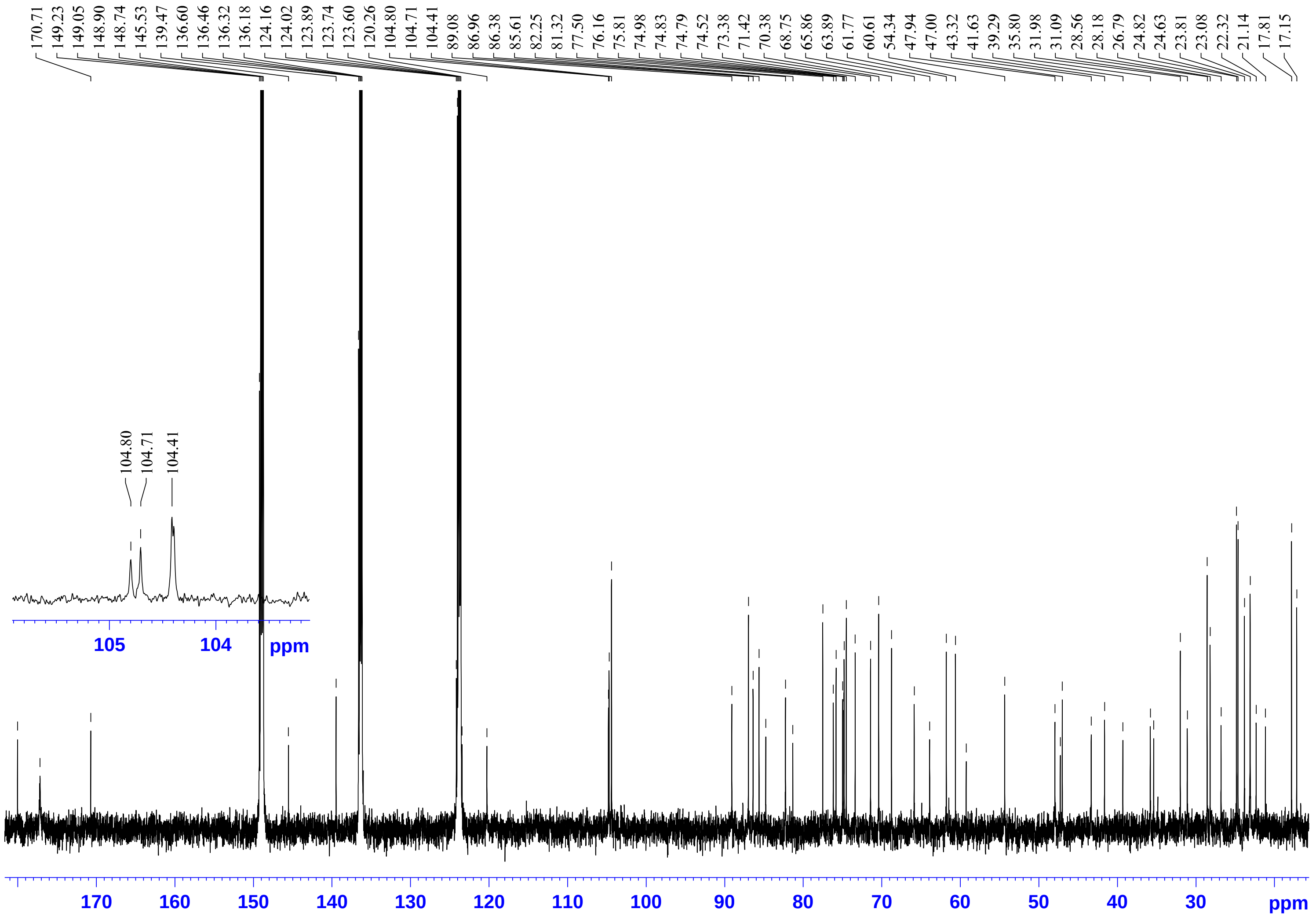

Fig. 1. The ^{13}C NMR (176.03 MHz) spectrum of quadrangularisoside A (**1**) in C_5D_5N/D_2O (4/1)

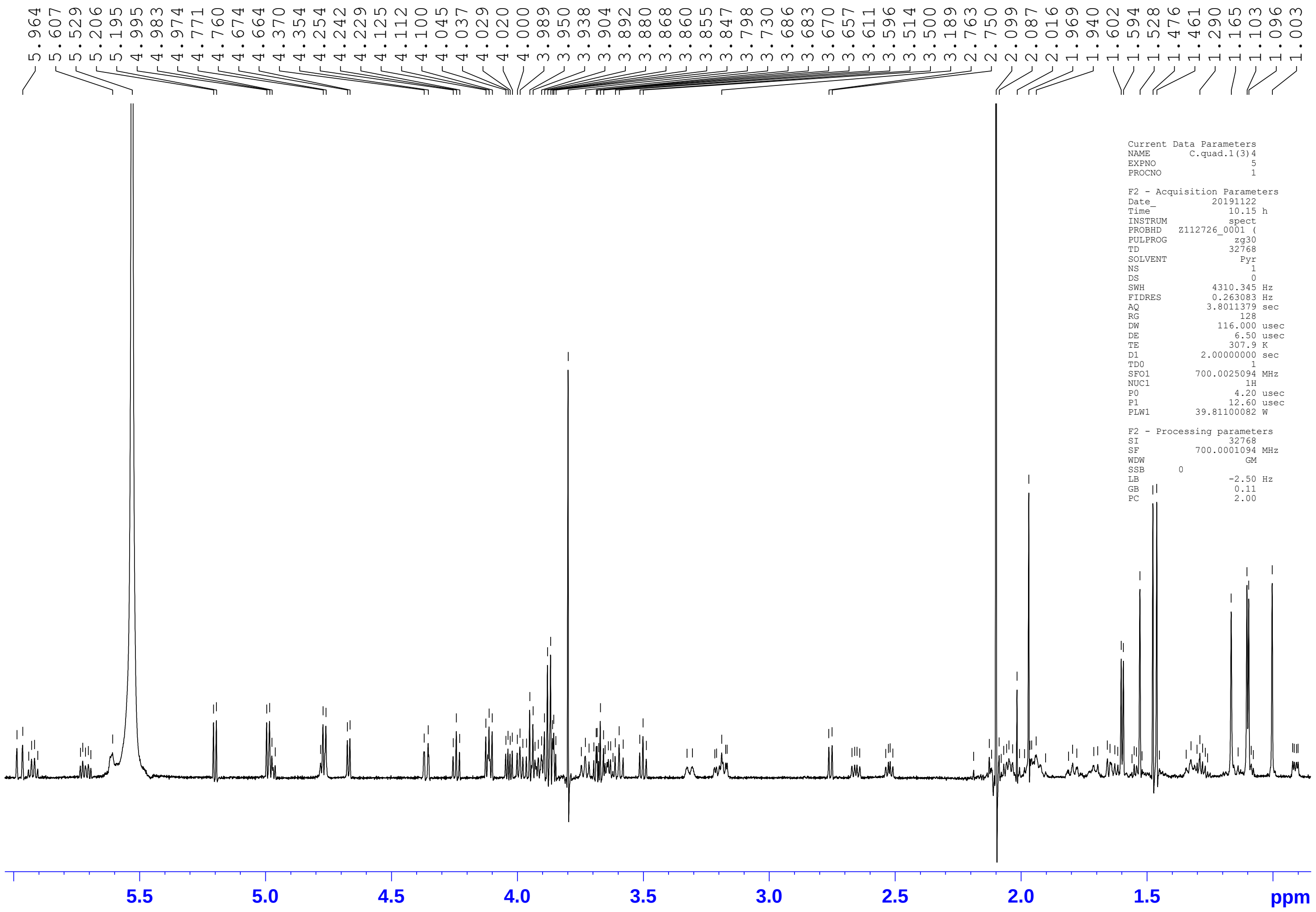

Fig. 2. The ^{1}H NMR (700.00 MHz) spectrum of quadrangularisoside A (**1**) in C_5D_5N/D_2O (4/1)

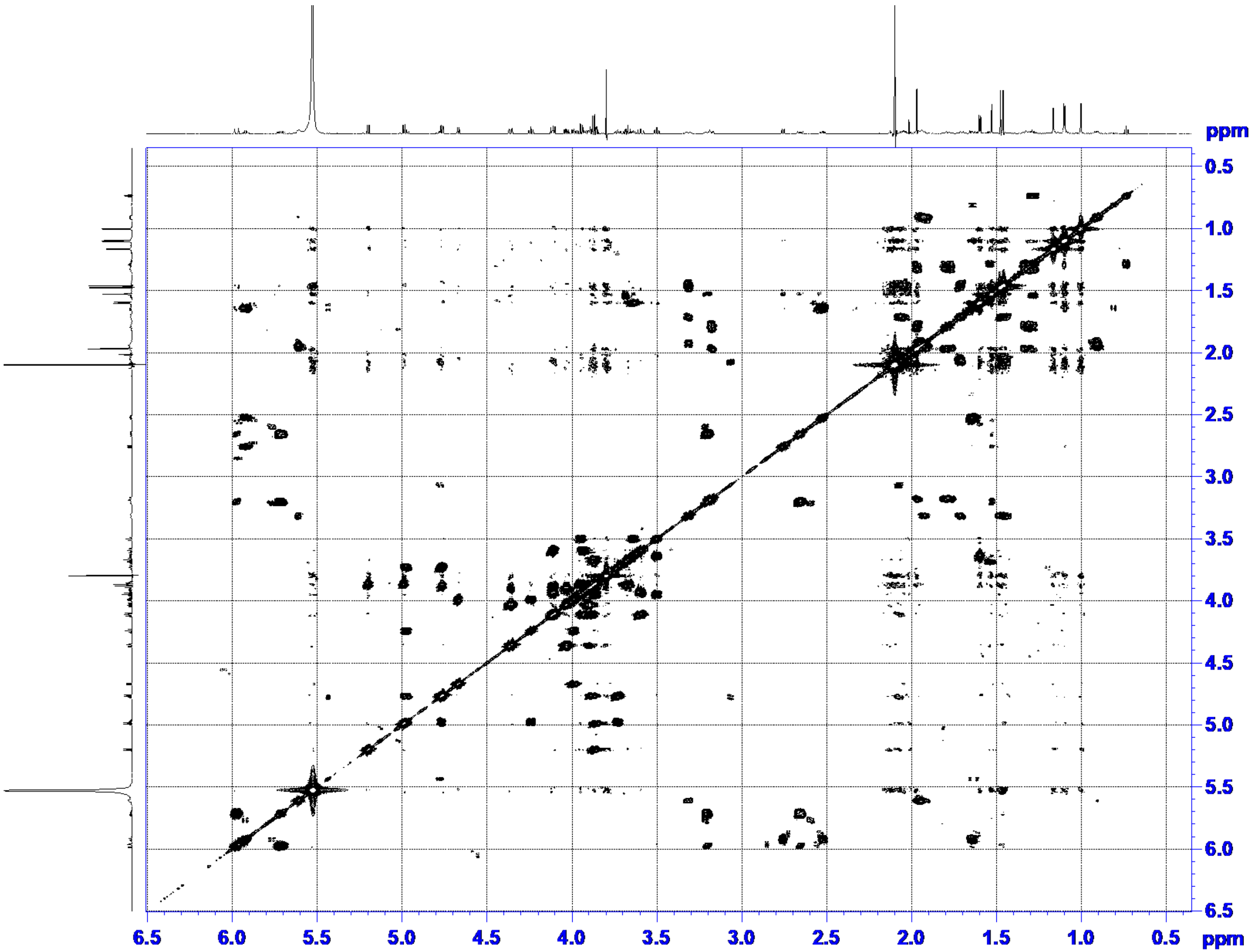

Fig. 3. The COSY (700.00 MHz) spectrum of quadrangularisoside A (**1**) in C_5D_5N/D_2O (4/1)

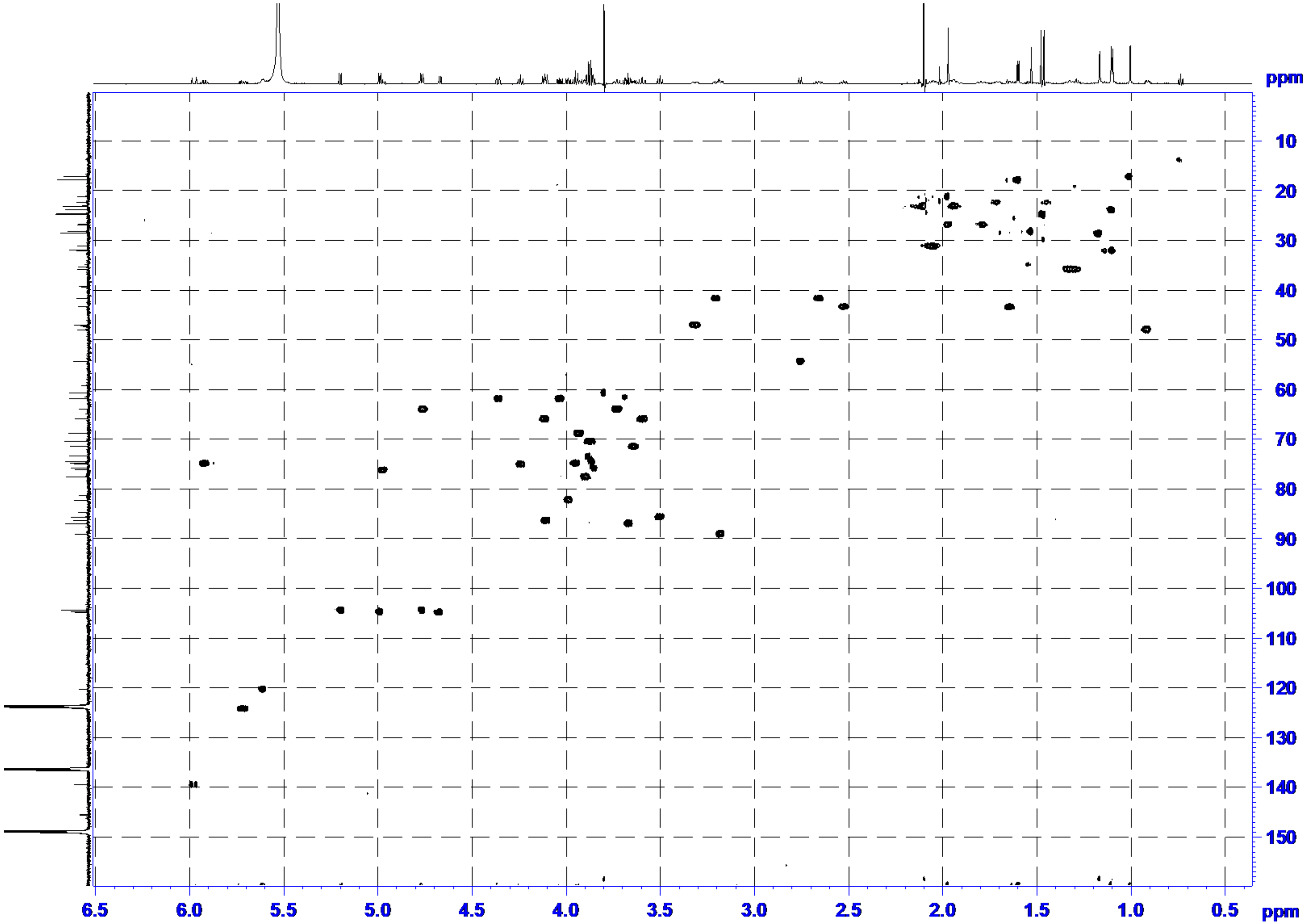

Fig. 4. The HSQC (700.00 MHz) spectrum of quadrangularisoside A (**1**) in C_5D_5N/D_2O (4/1)

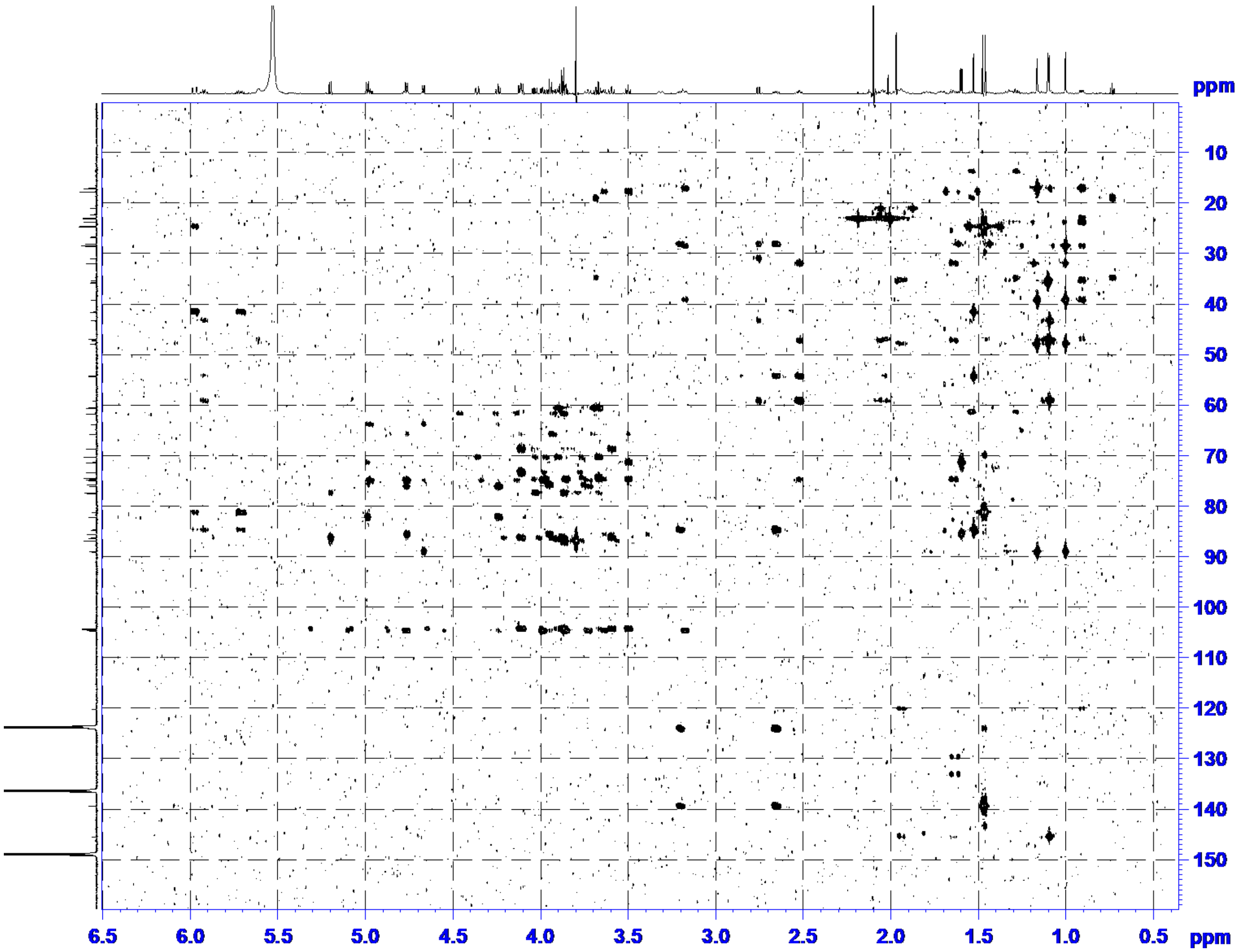

Fig. 5. The HMBC (700.00 MHz) spectrum of quadrangularisoside A (**1**) in C_5D_5N/D_2O (4/1)

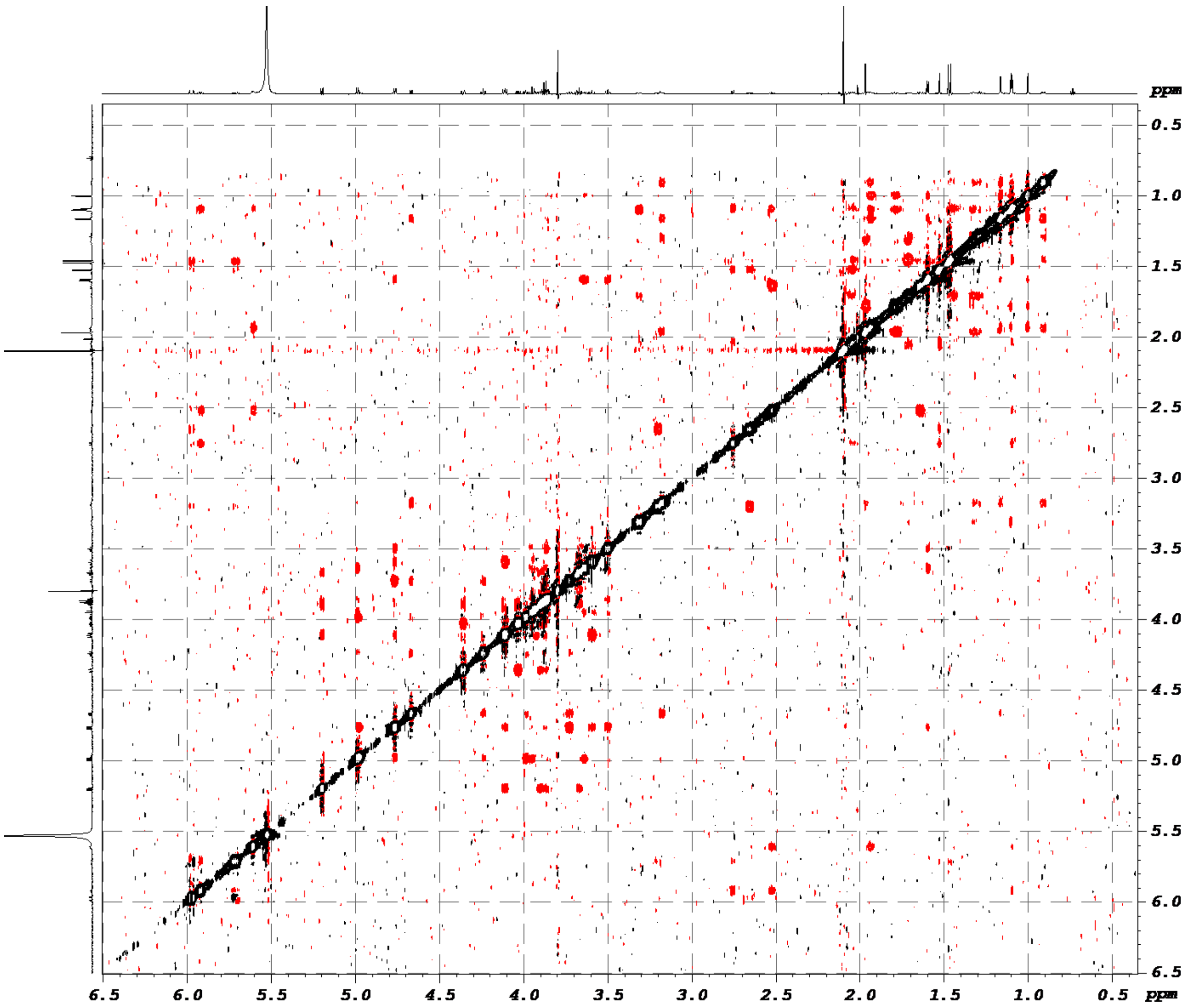

Fig. 6. The ROESY (700.00 MHz) spectrum of quadrangularisoside A (**1**) in C_5D_5N/D_2O (4/1)

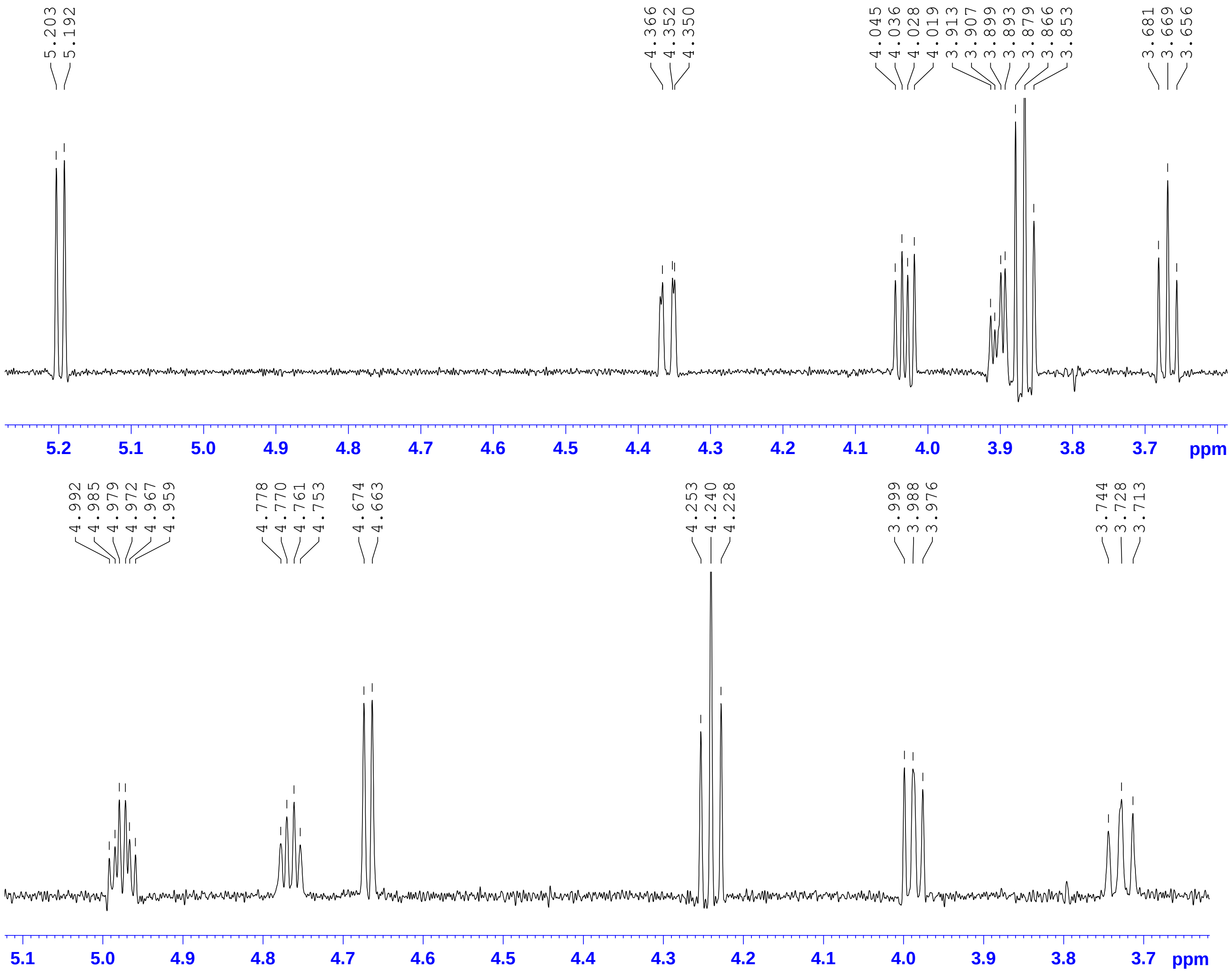

Fig. 7. 1 D TOCSY (700.00 MHz) spectra of quadrangularisoside A (**1**) in C_5D_5N/D_2O (4/1)

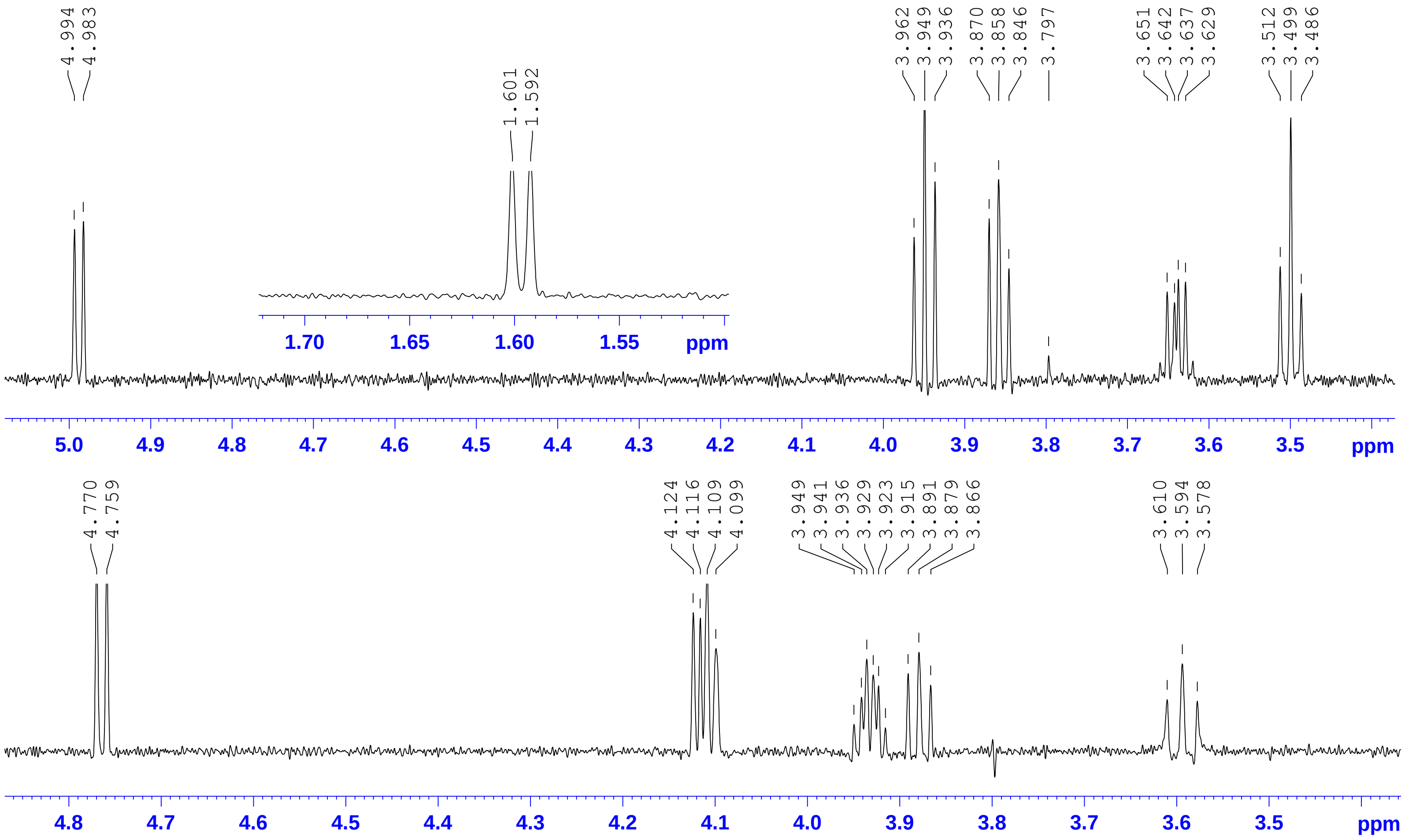

Fig. 8. 1 D TOCSY (700.00 MHz) spectra of quadrangularisoside A (**1**) in C_5D_5N/D_2O (4/1)

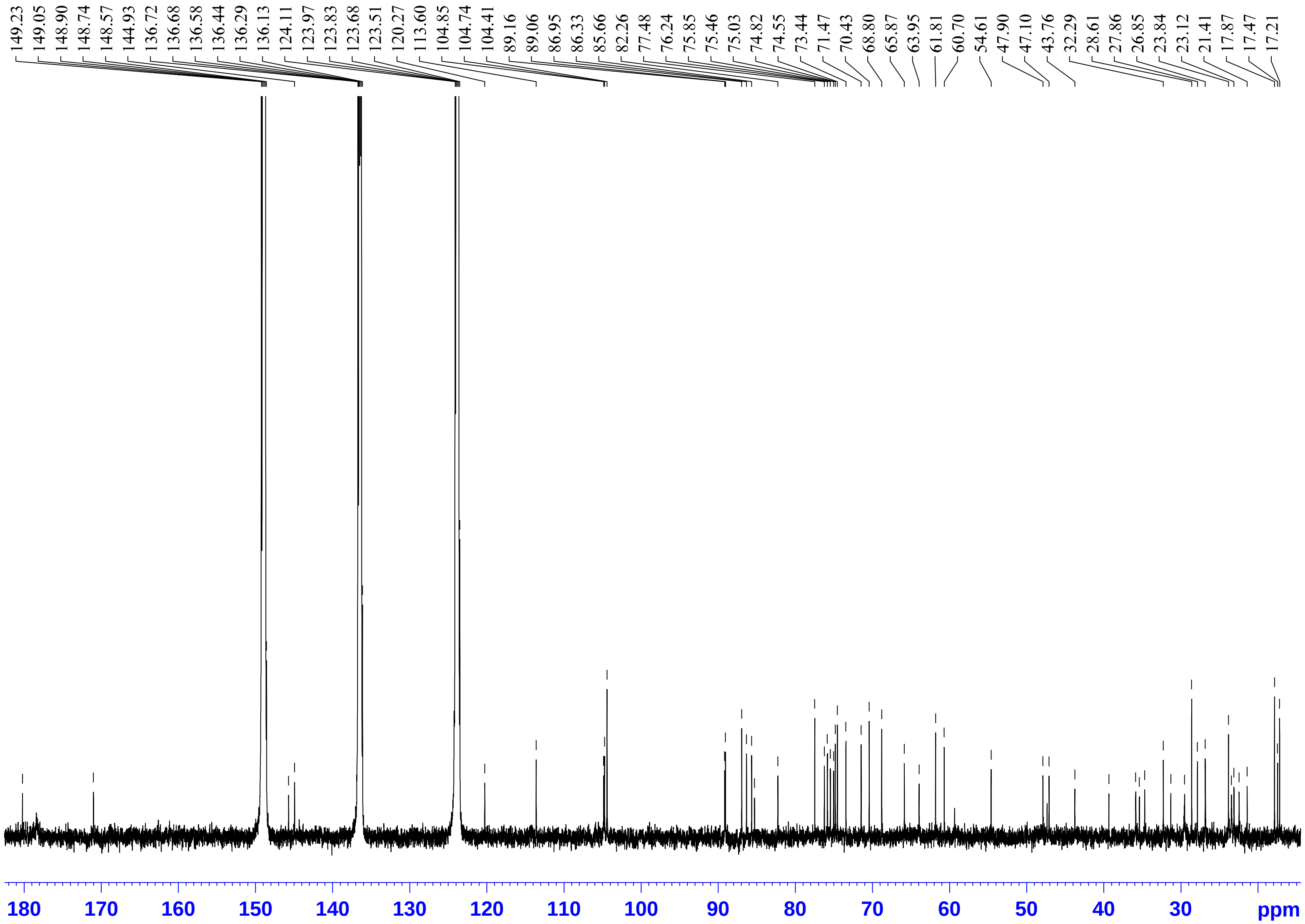

Fig. 9. The ^{13}C NMR (176.03 MHz) spectrum of quadrangularisoside A$_1$ (**2**) in C_5D_5N/D_2O (4/1)

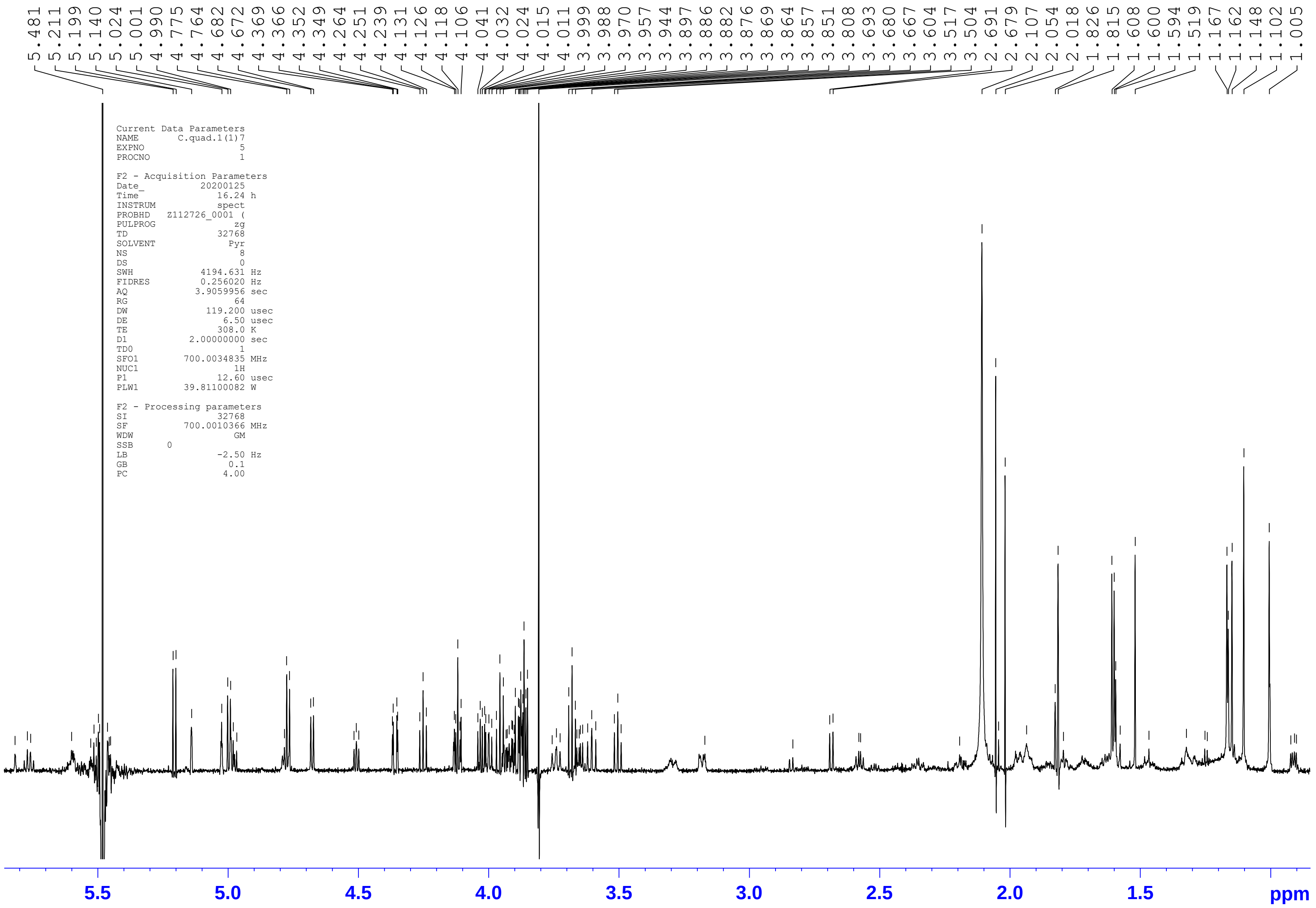

Fig. 10. The ^{1}H NMR (700.00 MHz) spectrum of quadrangularisoside A$_1$ (**2**) in C_5D_5N/D_2O (4/1)

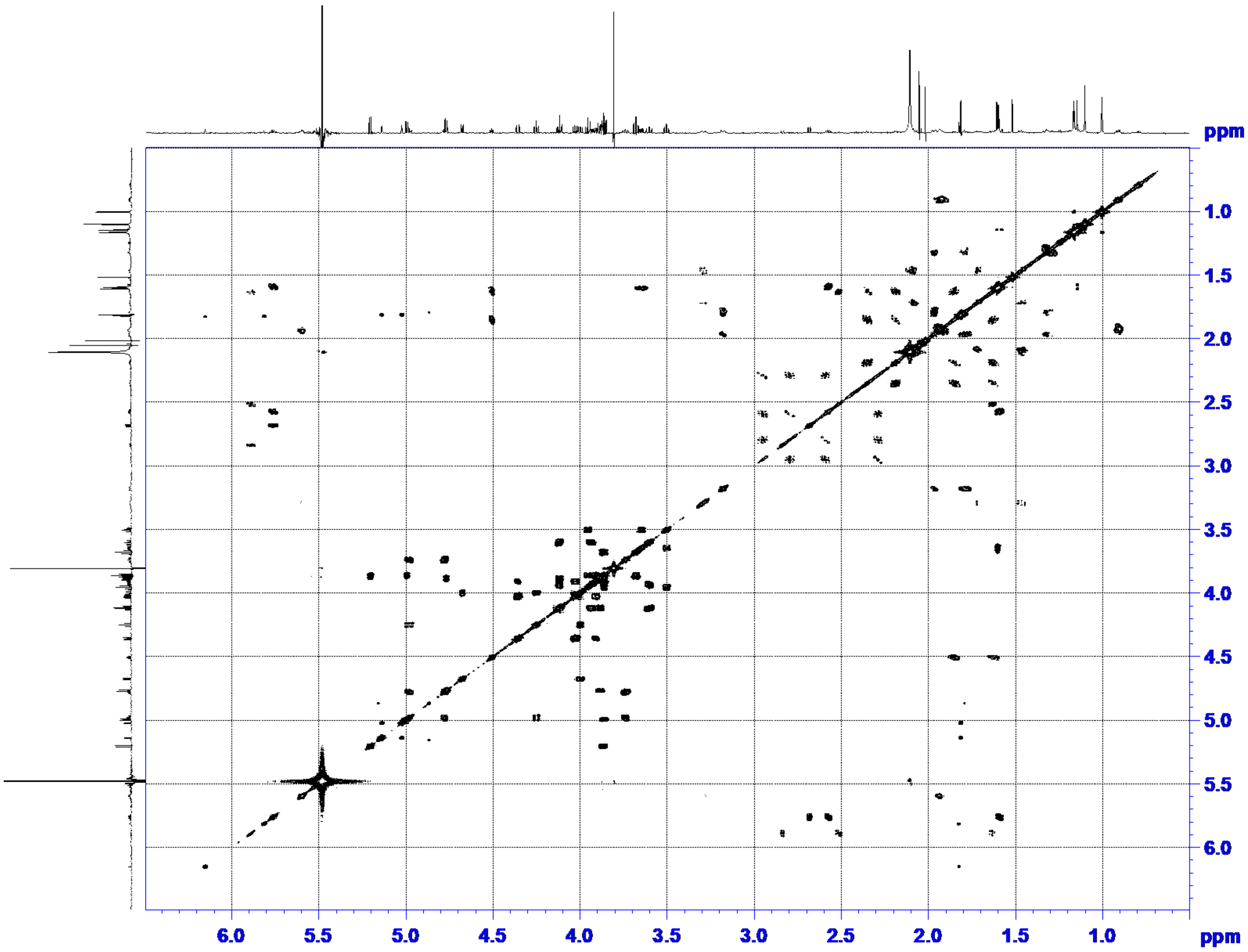

Fig. 11. The COSY (700.00 MHz) spectrum of quadrangularisoside A1 (**2**) in C_5D_5N/D_2O (4/1)

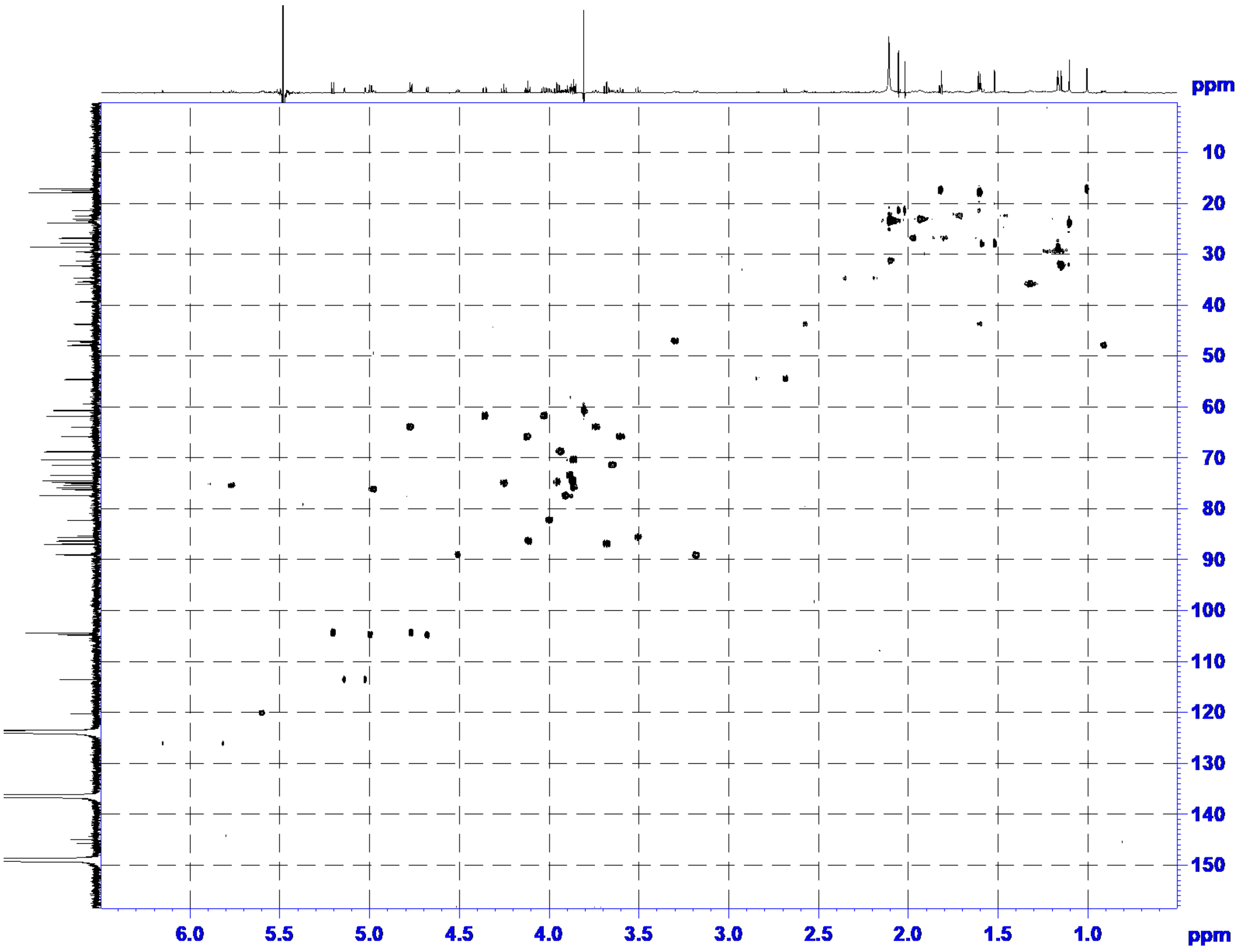

Fig. 12. The HSQC (700.00 MHz) spectrum of quadrangularisoside A1 (**2**) in C_5D_5N/D_2O (4/1)

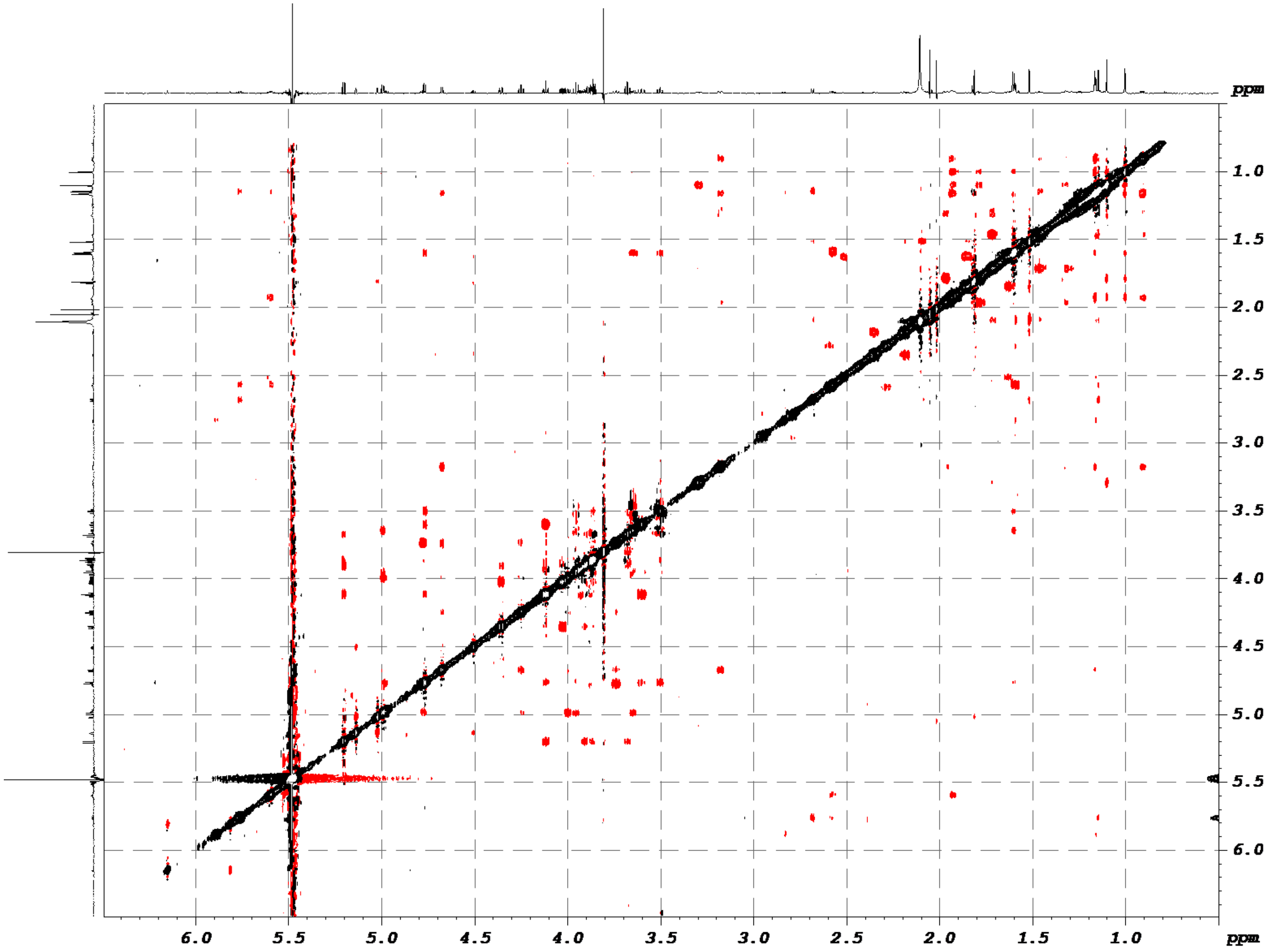

Fig. 13. The ROESY (700.00 MHz) spectrum of quadrangularisoside A_1 (**2**) in C_5D_5N/D_2O (4/1)

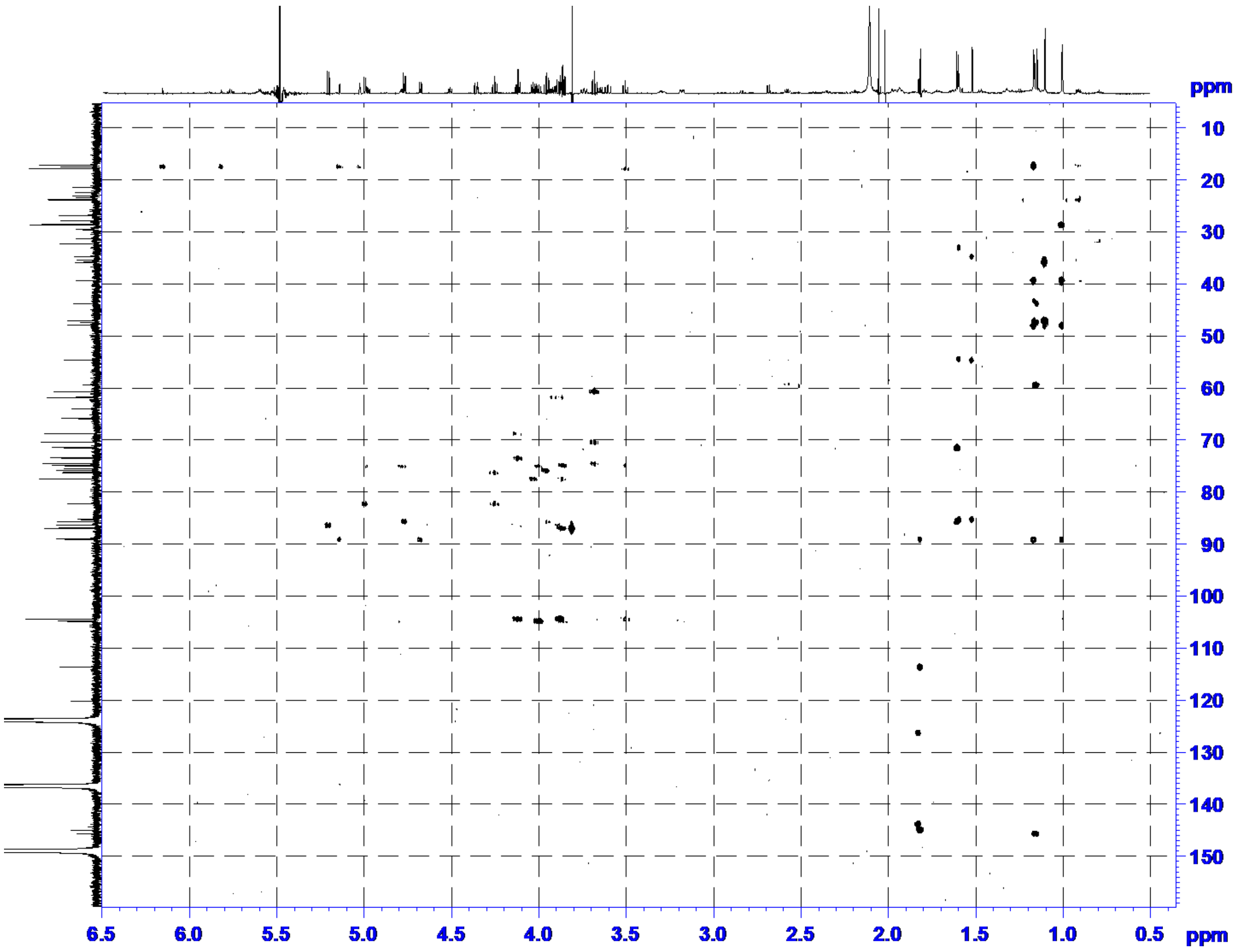

Fig. 14. The HMBC (700.00 MHz) spectrum of quadrangularisoside A1 (**2**) in C_5D_5N/D_2O (4/1)

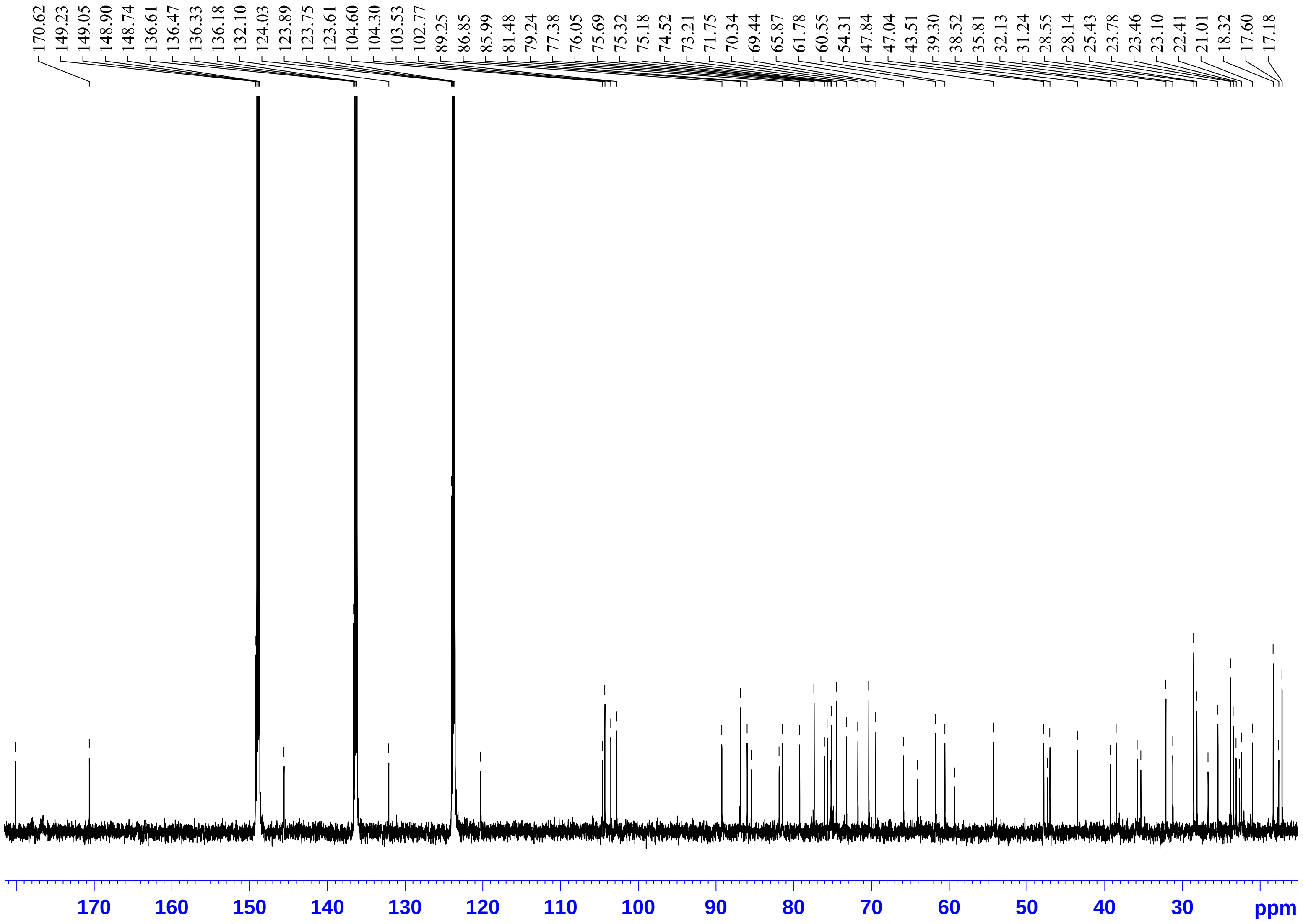

Fig. 15. The ^{13}C NMR (176.03 MHz) spectrum of quadrangularisoside B (**3**) in C_5D_5N/D_2O (4/1)

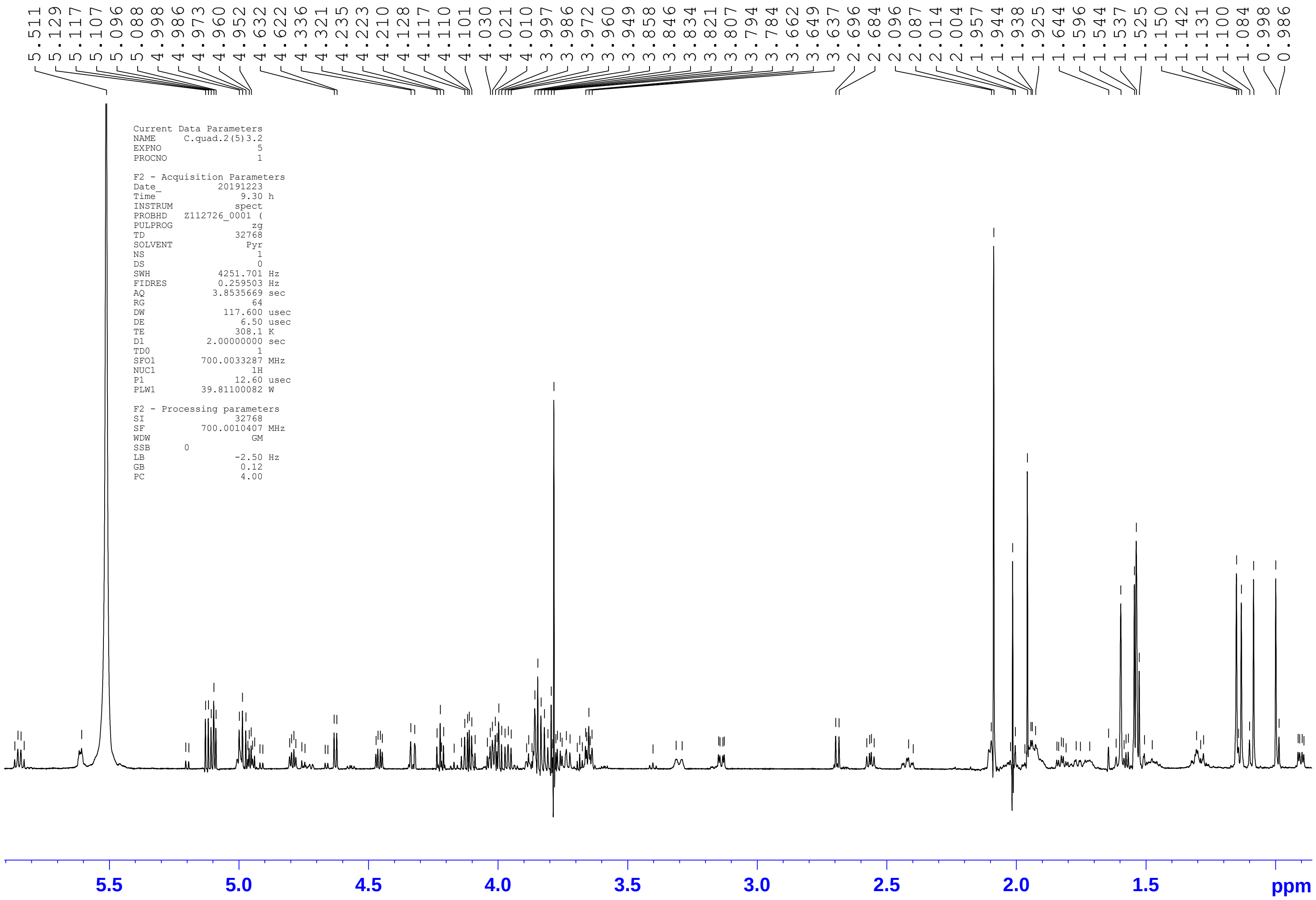

Fig. 16. The ^{1}H NMR (700.00 MHz) spectrum of quadrangularisoside B (**3**) in C_5D_5N/D_2O (4/1)

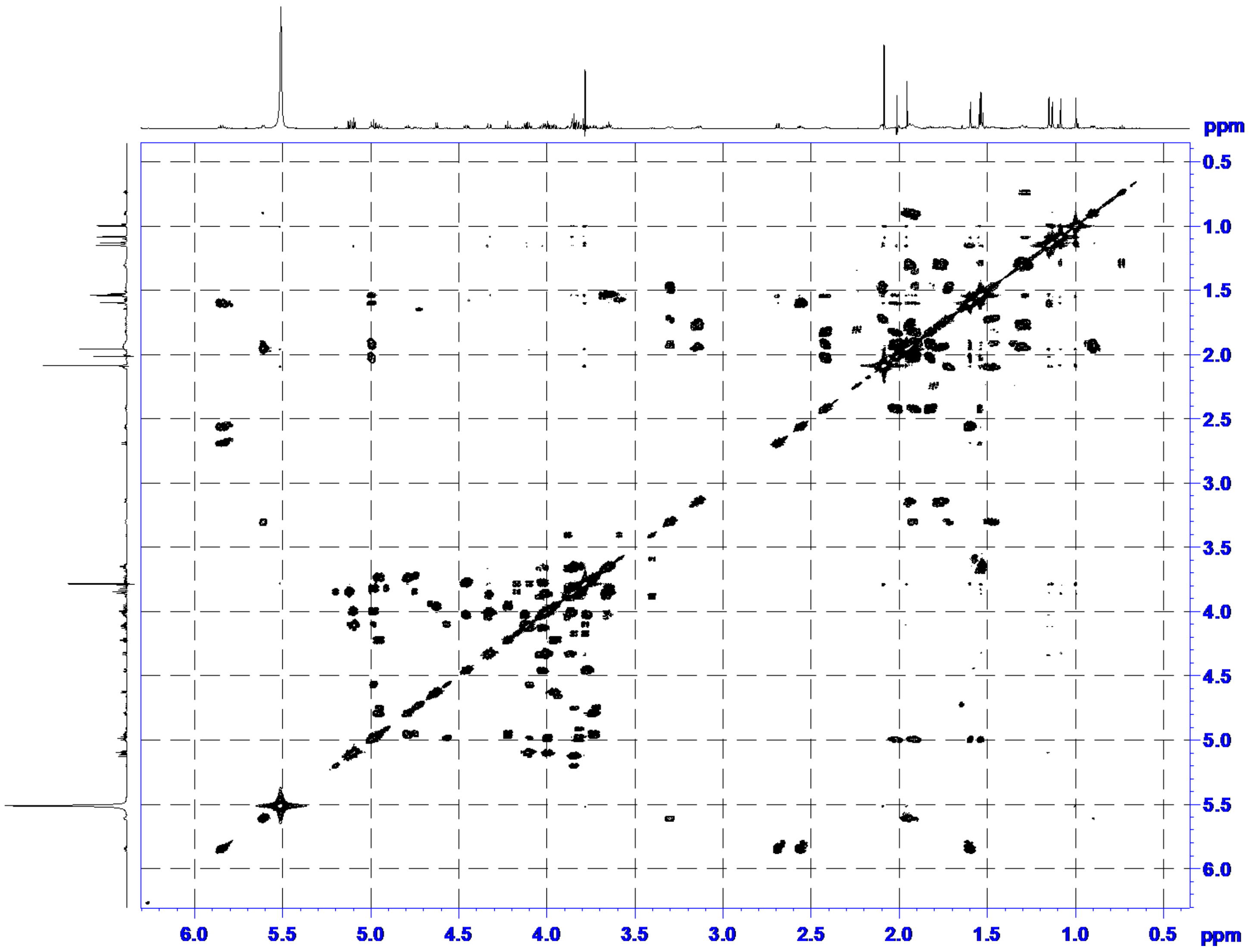

Fig. 17. The COSY (700.00 MHz) spectrum of quadrangularisoside B (**3**) in C_5D_5N/D_2O (4/1)

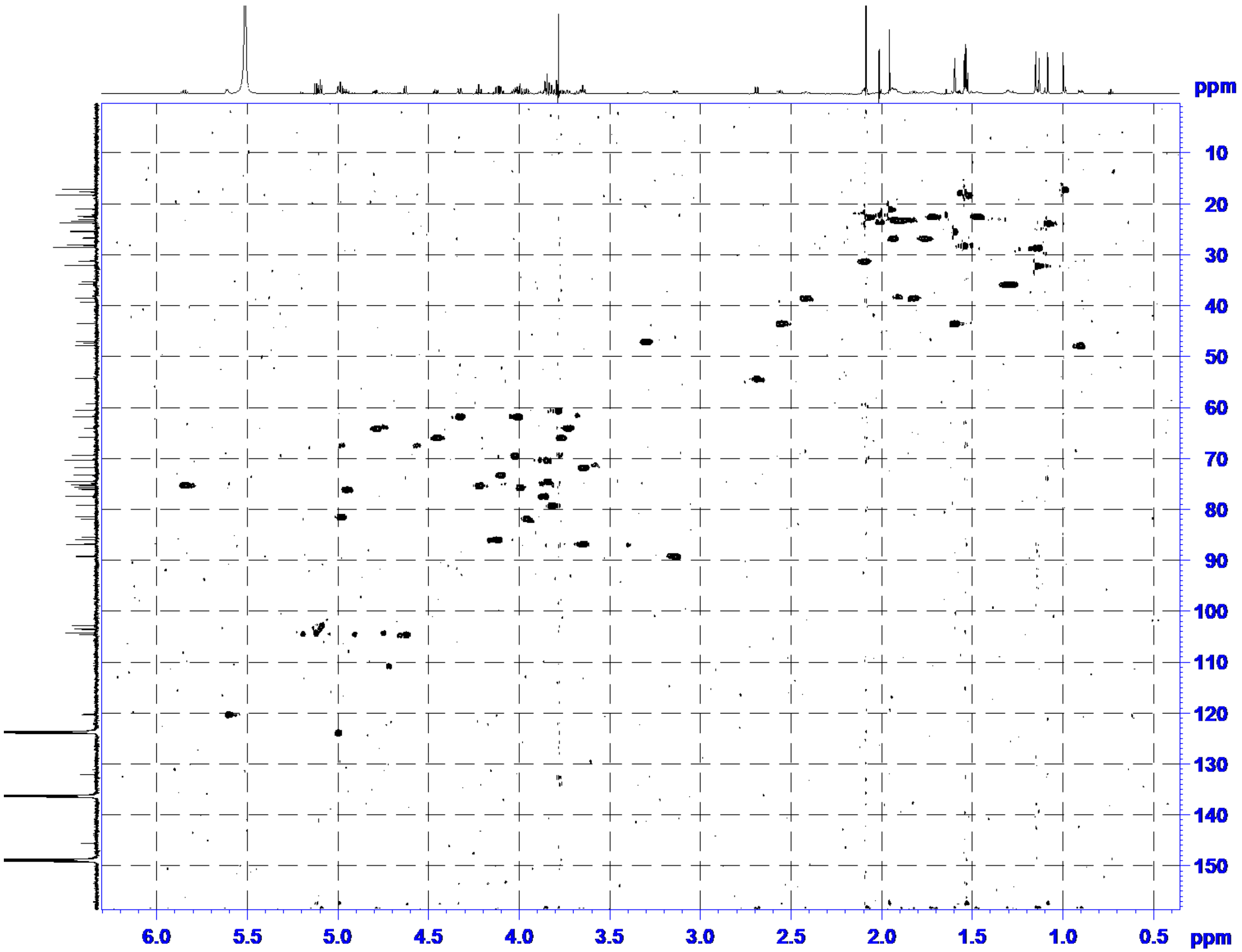

Fig. 18. The HSQC (700.00 MHz) spectrum of quadrangularisoside B (**3**) in C_5D_5N/D_2O (4/1)

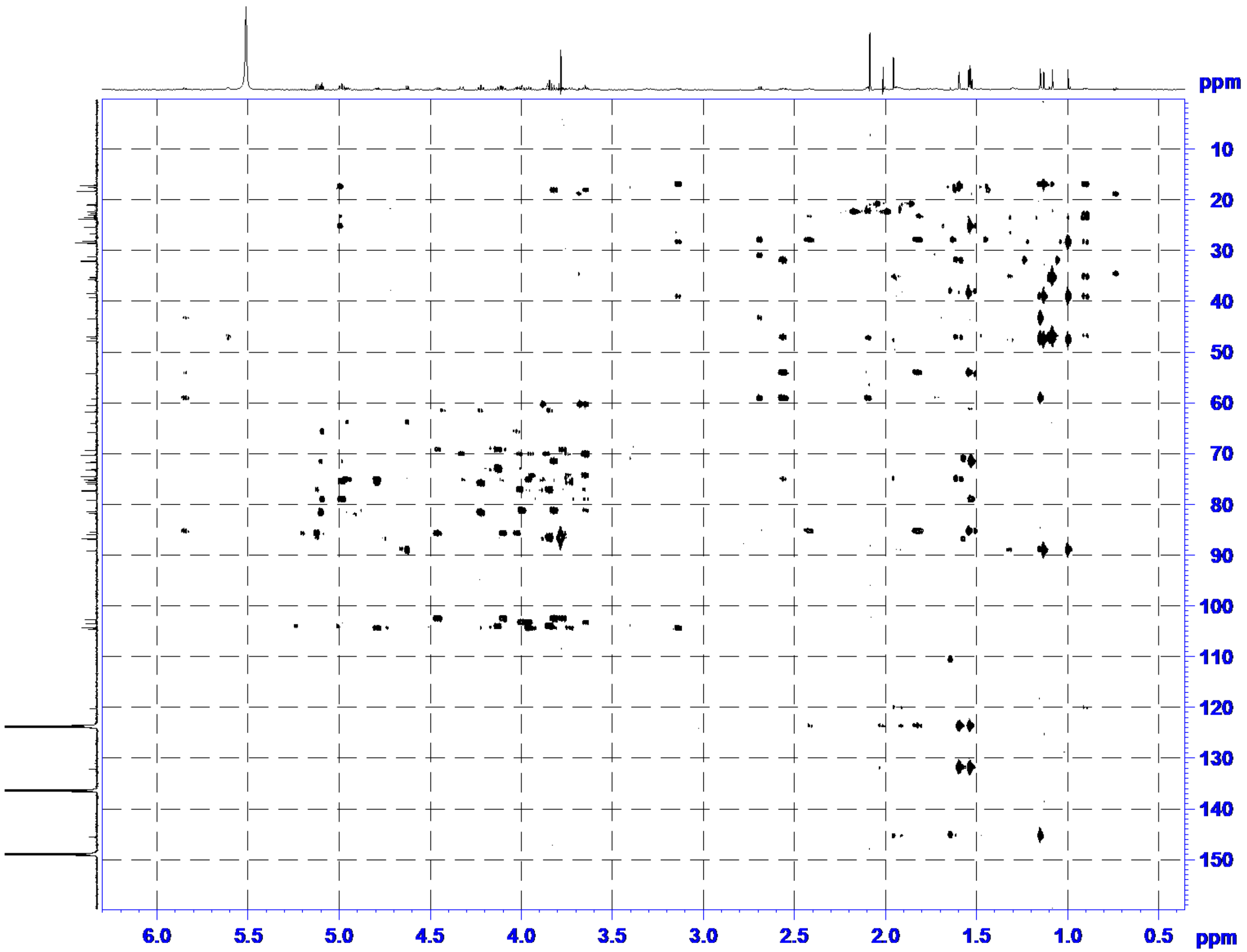

Fig. 19. The HMBC (700.00 MHz) spectrum of quadrangularisoside B (**3**) in C_5D_5N/D_2O (4/1)

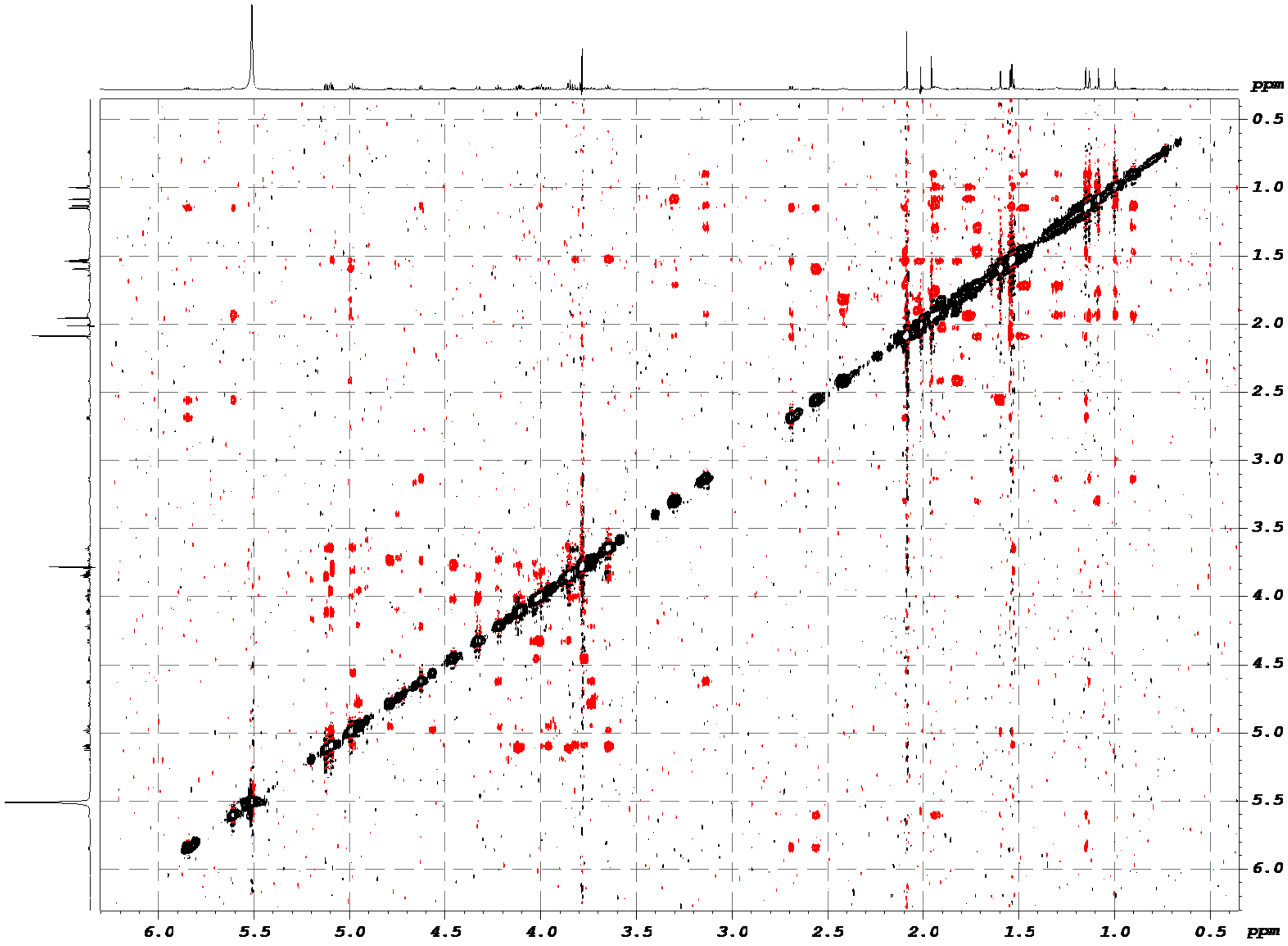

Fig. 20. The ROESY (700.00 MHz) spectrum of quadrangularisoside B (**3**) in C_5D_5N/D_2O (4/1)

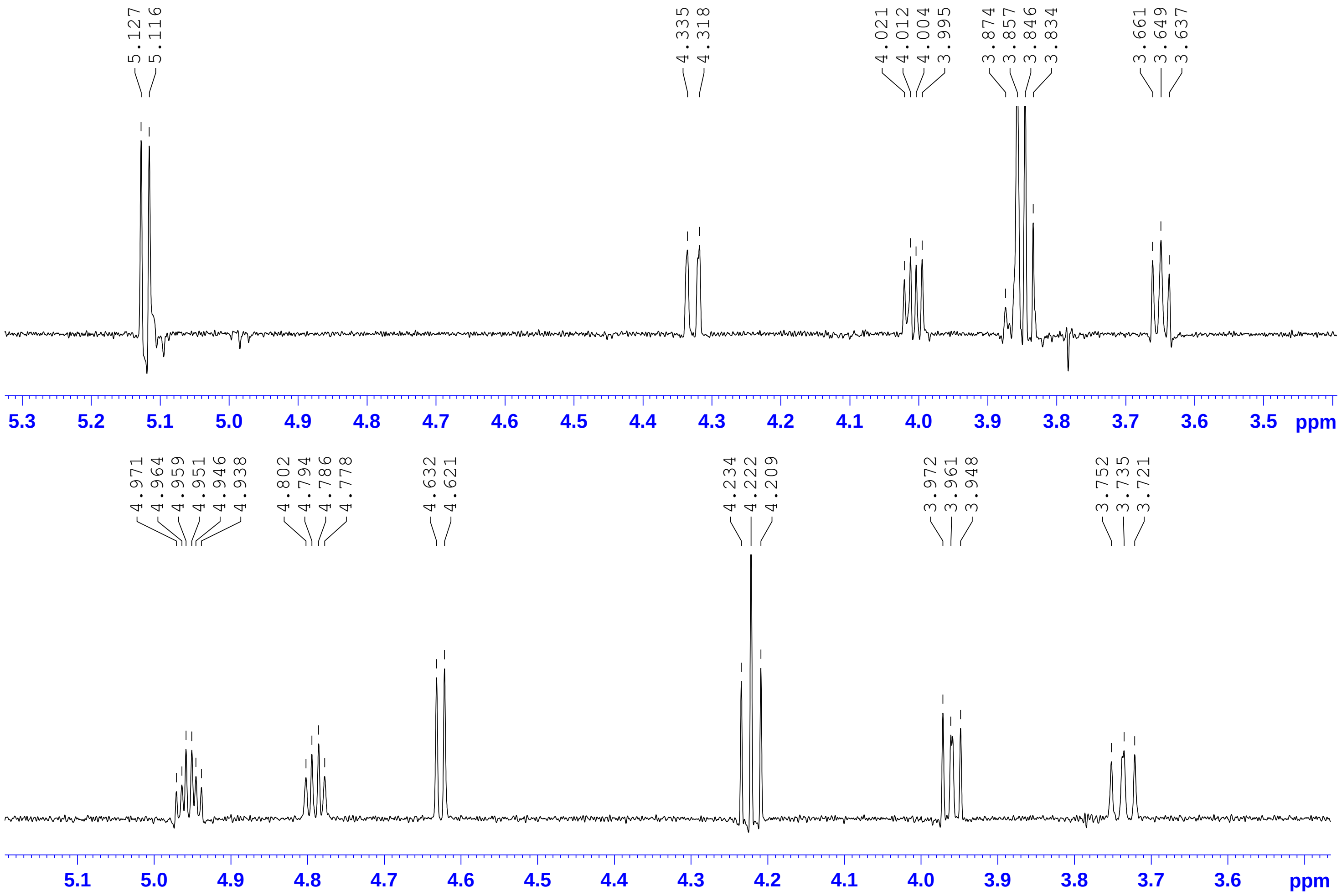

Fig. 21. 1D TOCSY (700.00 MHz) spectra of quadrangularisoside B (**3**) in C_5D_5N/D_2O (4/1)

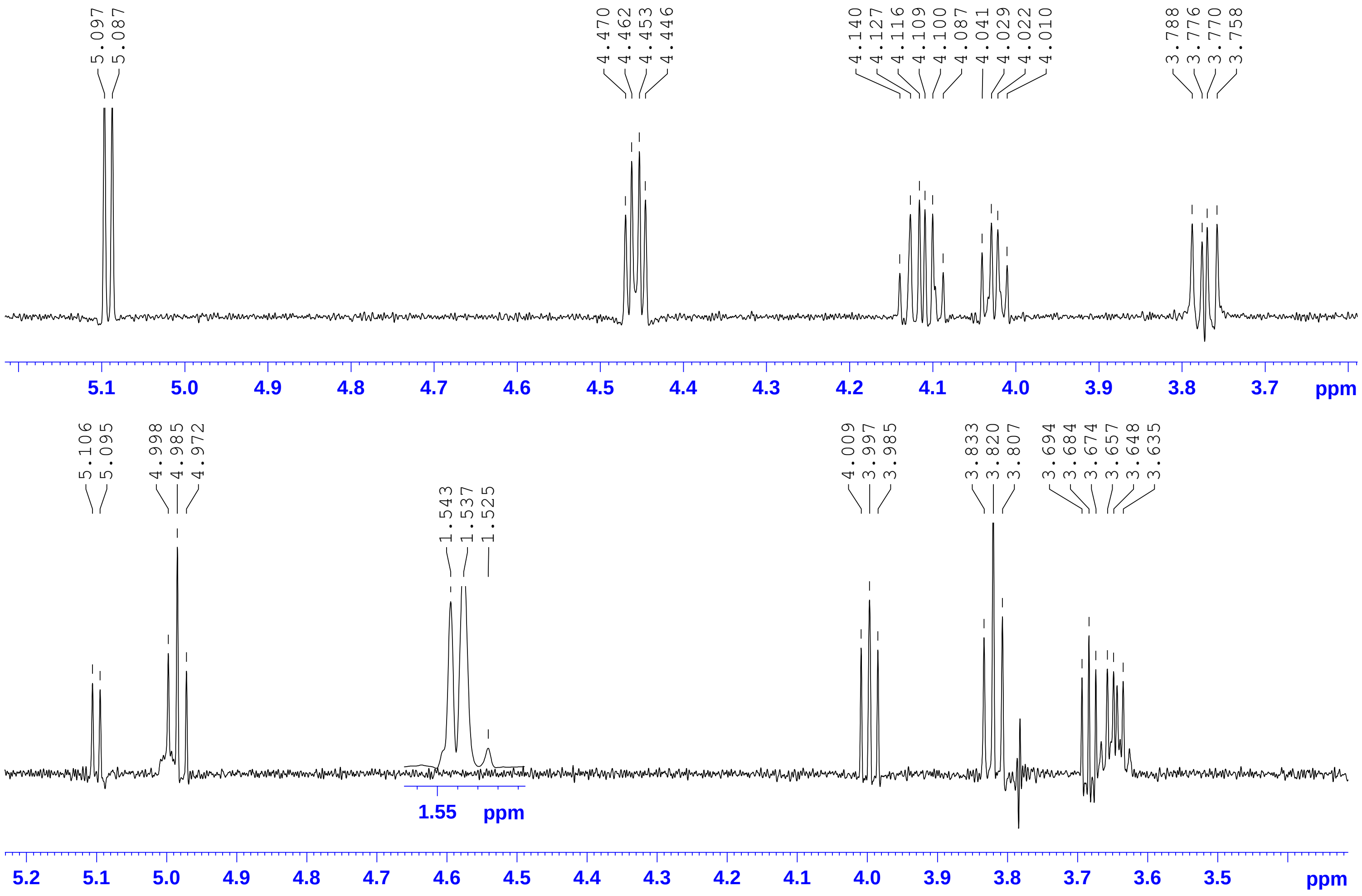

Fig. 22. 1D TOCSY (700.00 MHz) spectra of quadrangularisoside B (**3**) in C_5D_5N/D_2O (4/1)

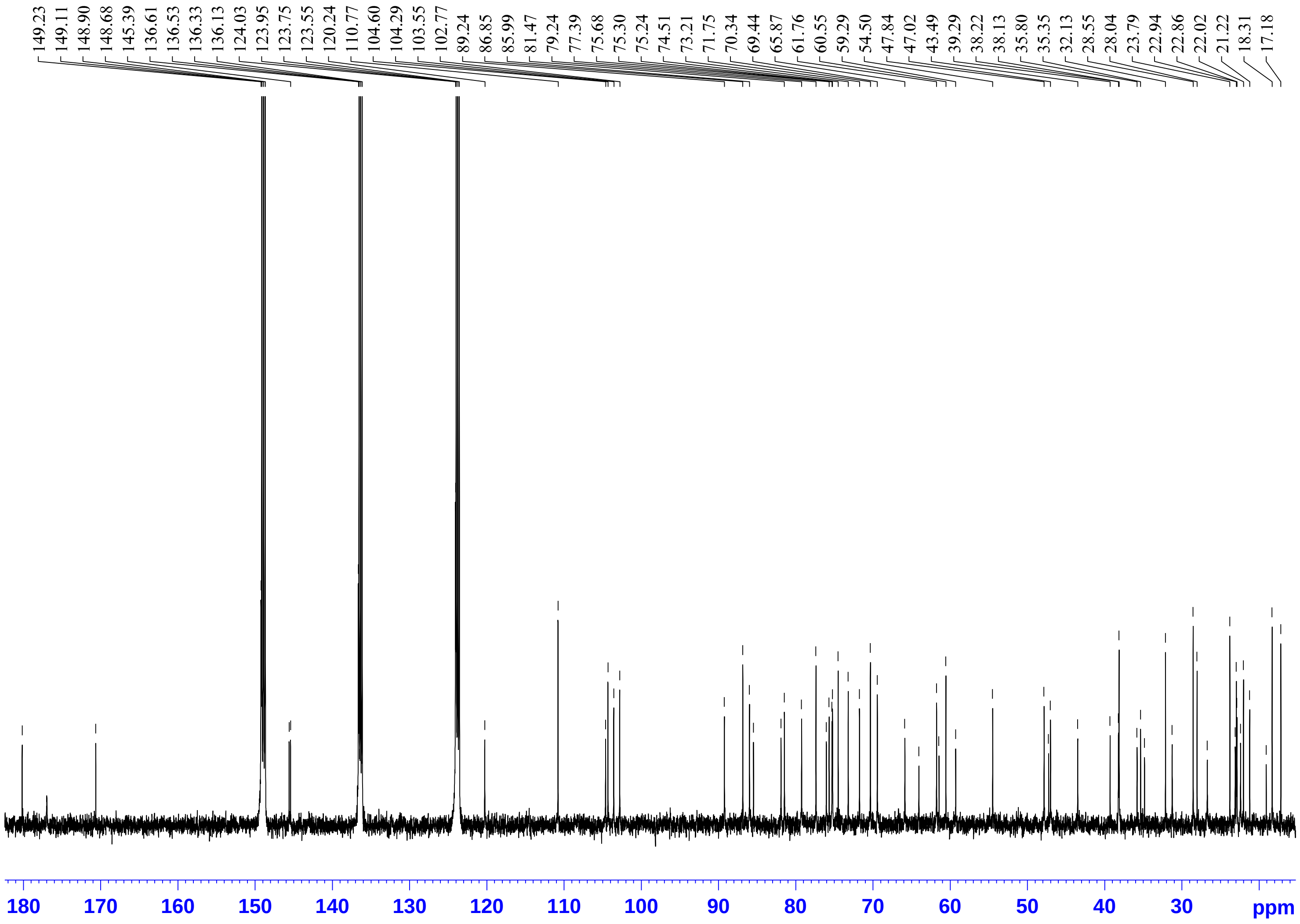

Fig. 23. The ^{13}C NMR (176.03 MHz) spectrum of quadrangularisoside B_1 (**4**) in C_5D_5N/D_2O (4/1)

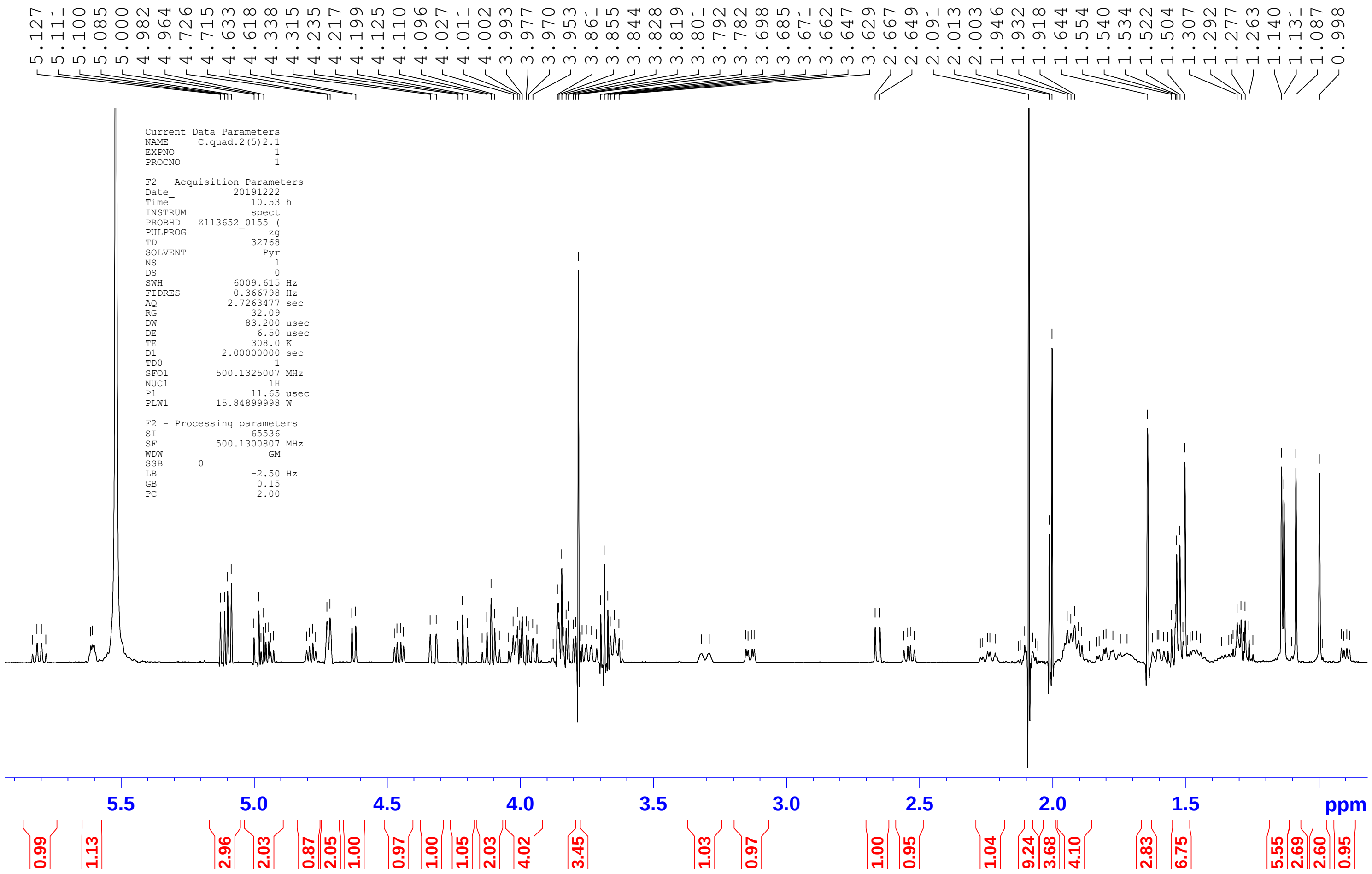

Fig. 24. The ¹H NMR (700.00 MHz) spectrum of quadrangularisoside B_1 (**4**) in C_5D_5N/D_2O (4/1)

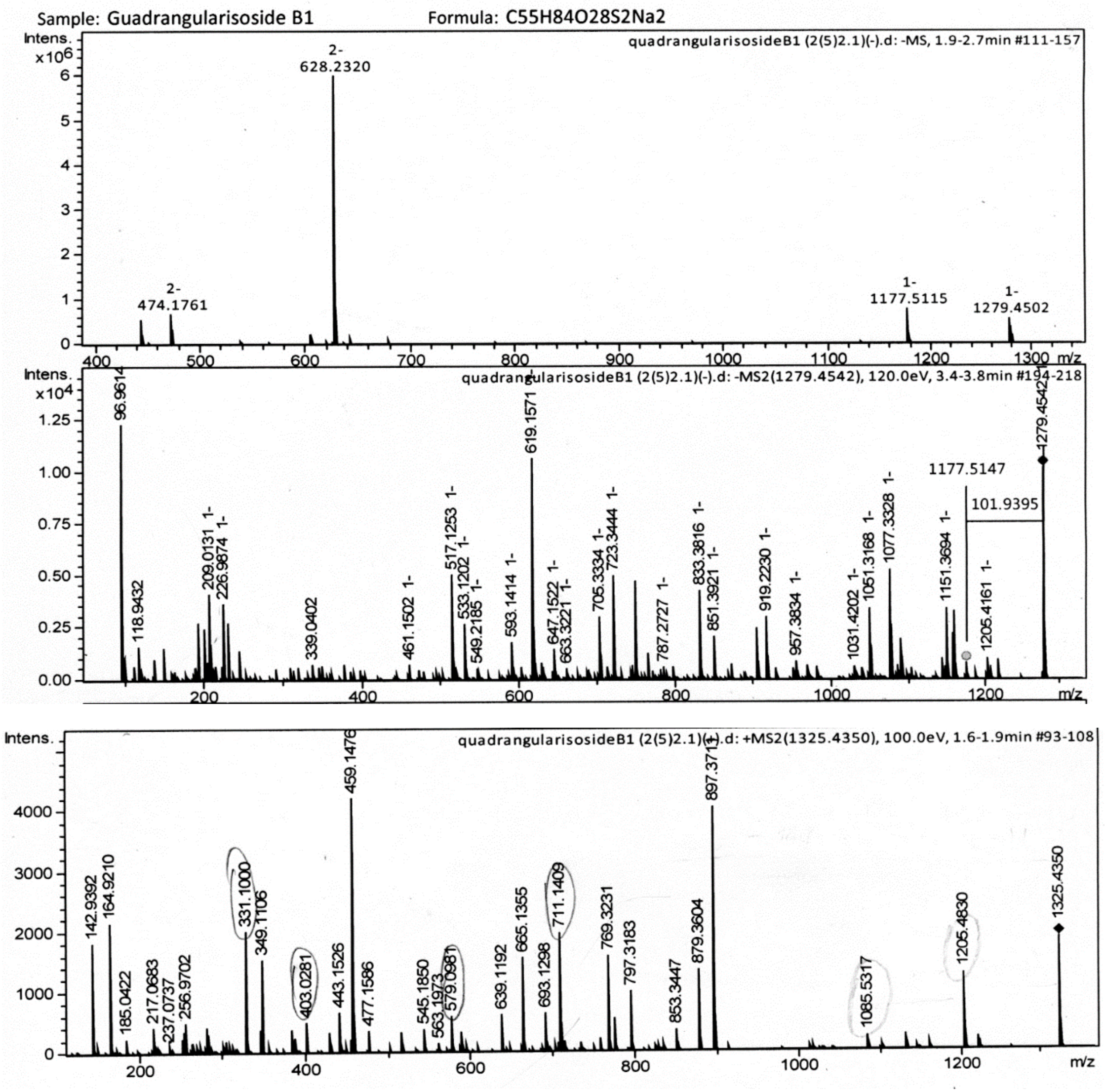

Fig. 25. HR-ESI-MS and ESI-MS/MS spectra of quadrangularisoside B$_1$ (**4**)

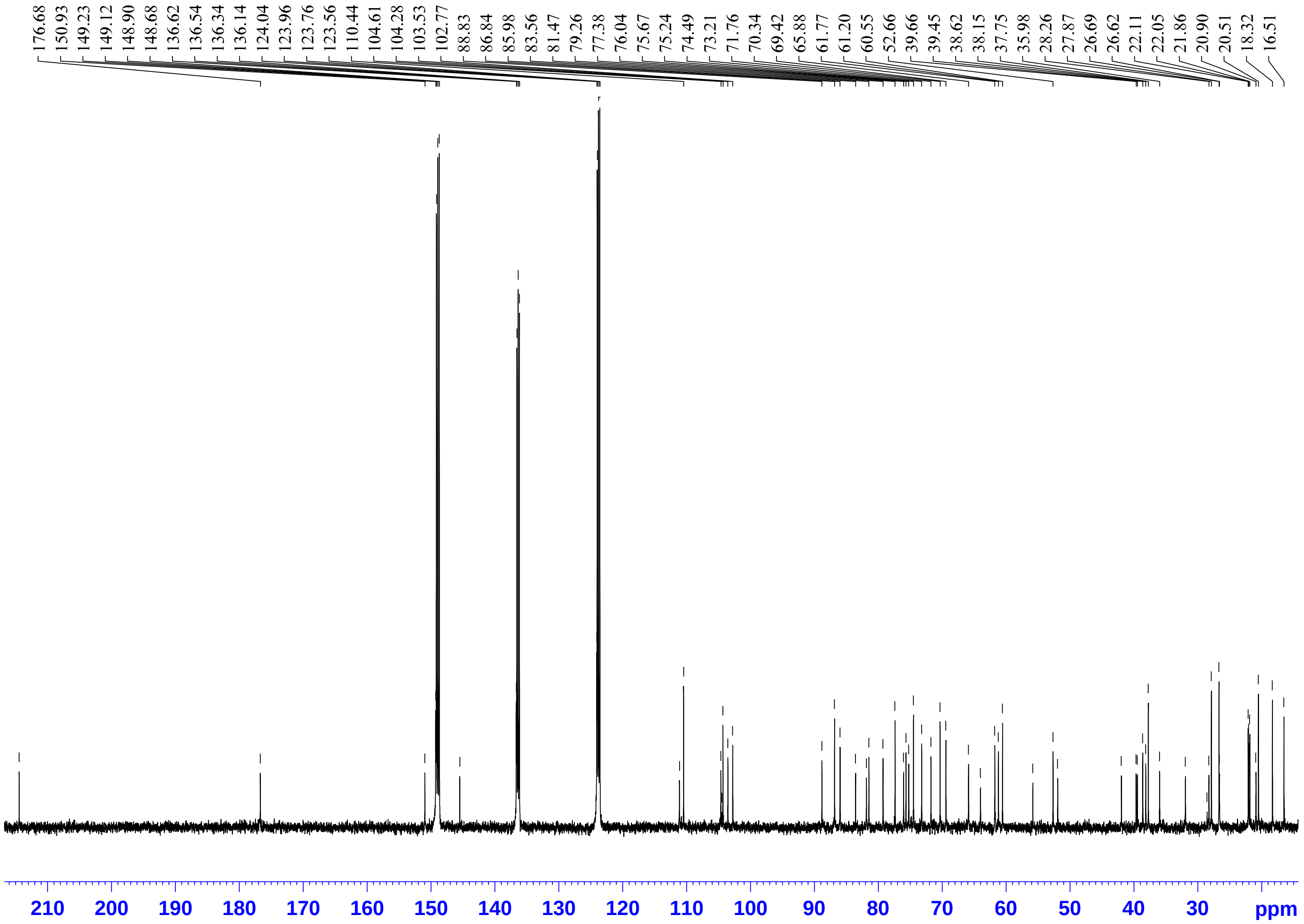

Fig. 26. The ^{13}C NMR (176.03 MHz) spectrum of quadrangularisoside B_2 (**5**) in C_5D_5N/D_2O (4/1)

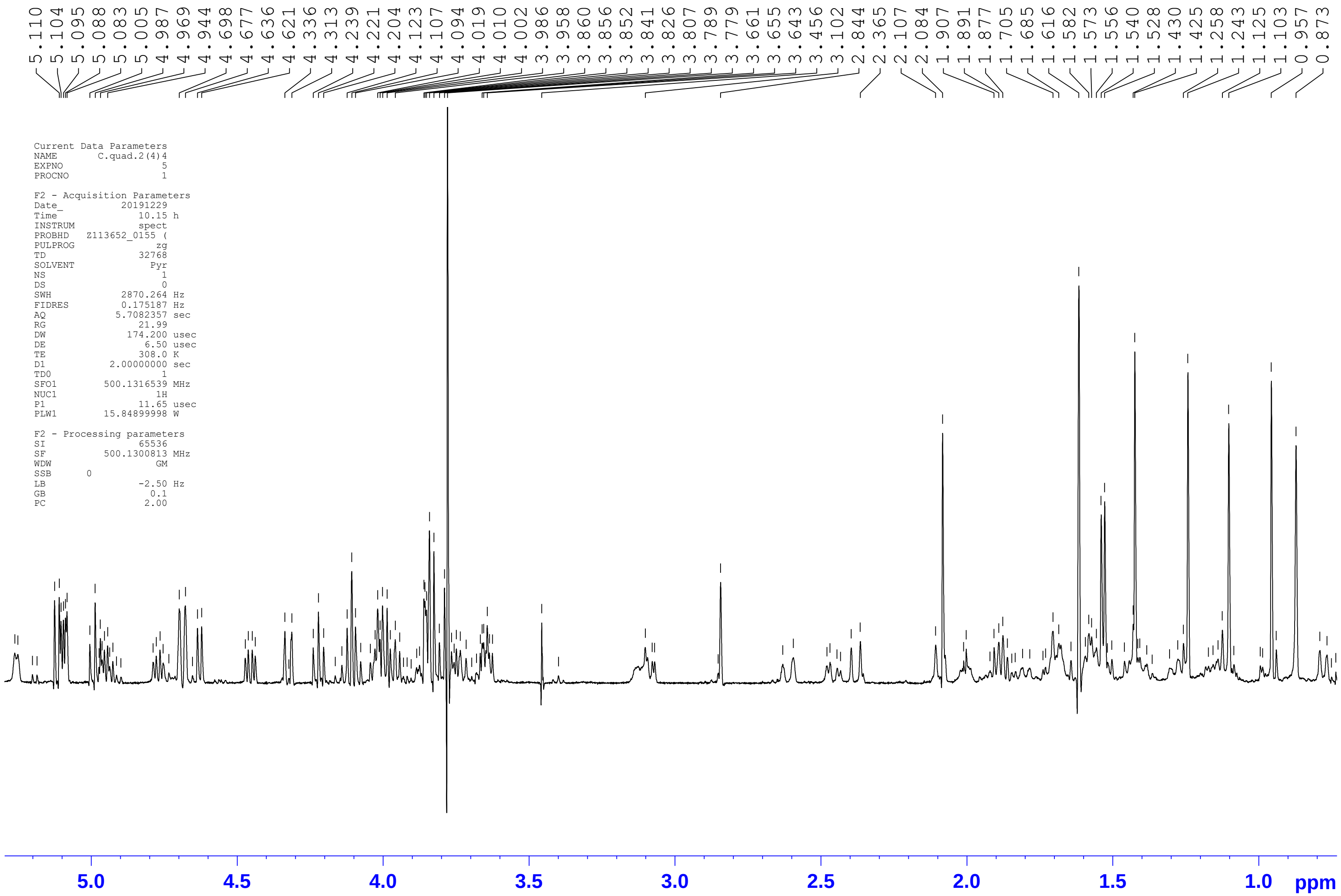

Fig. 27. The ^{1}H NMR (700.00 MHz) spectrum of quadrangularisoside B$_2$ (**5**) in C_5D_5N/D_2O (4/1)

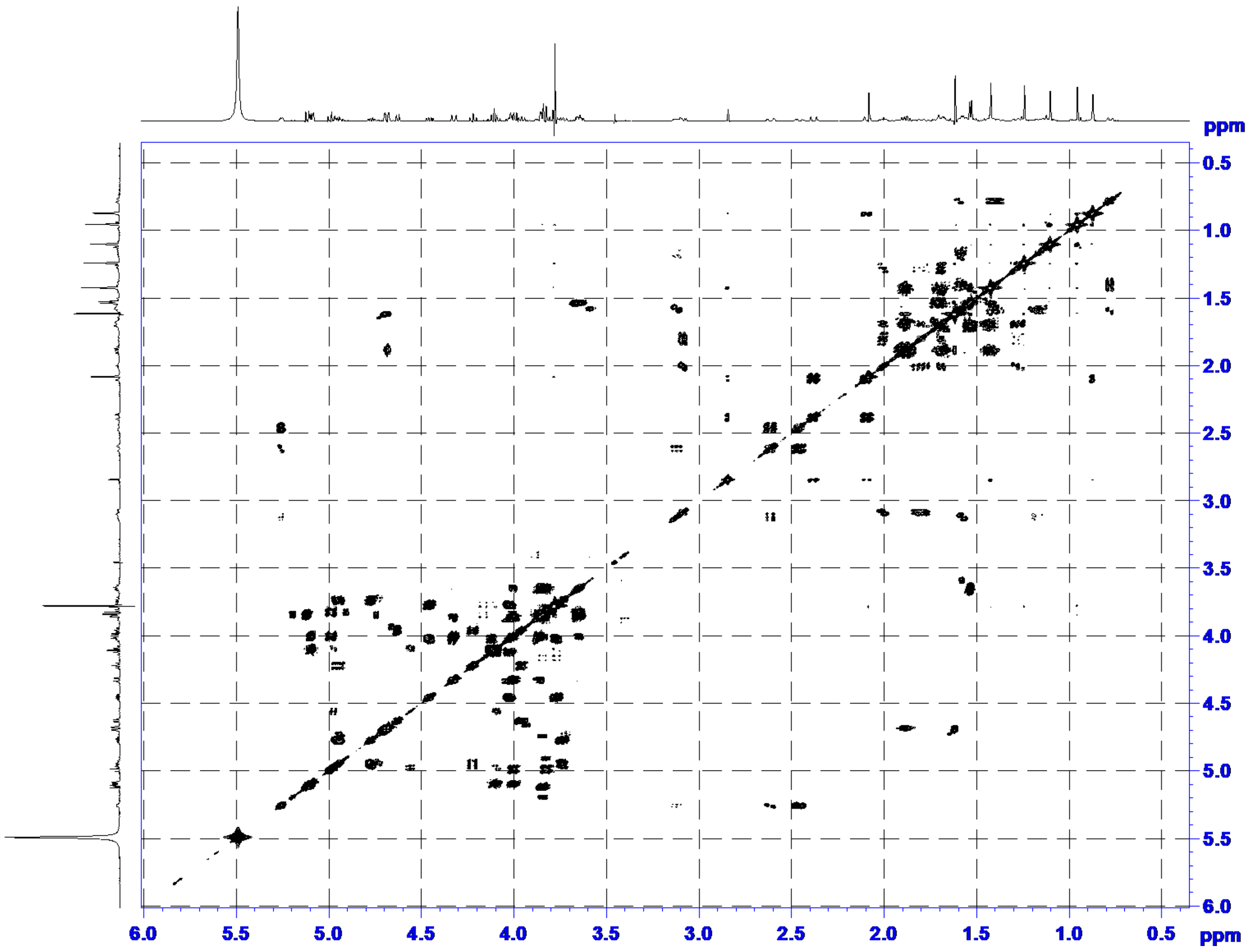

Fig. 28. The COSY (700.00 MHz) spectrum of quadrangularisoside B_2 (**5**) in C_5D_5N/D_2O (4/1)

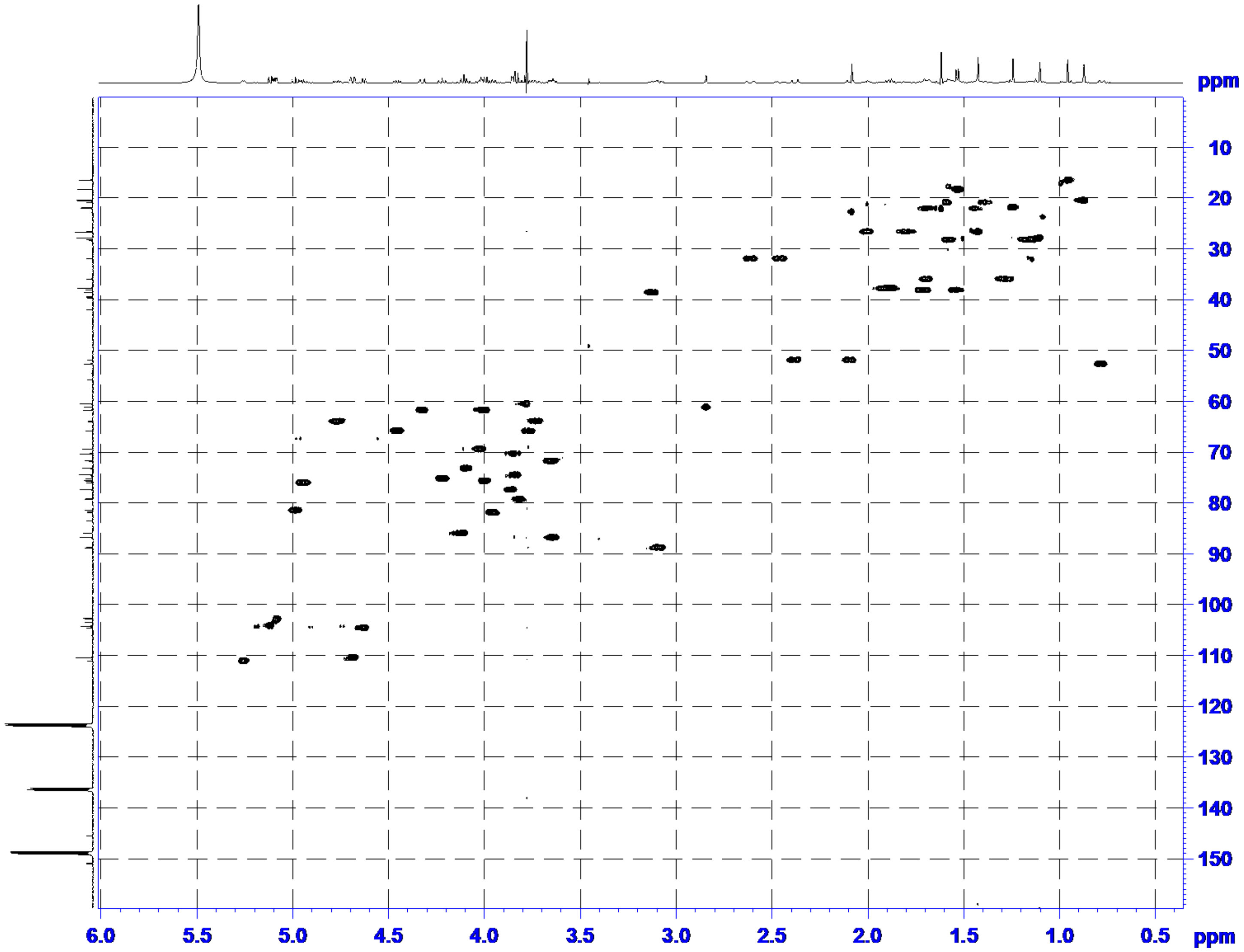

Fig. 29. The HSQC (700.00 MHz) spectrum of quadrangularisoside B2 (**5**) in C_5D_5N/D_2O (4/1)

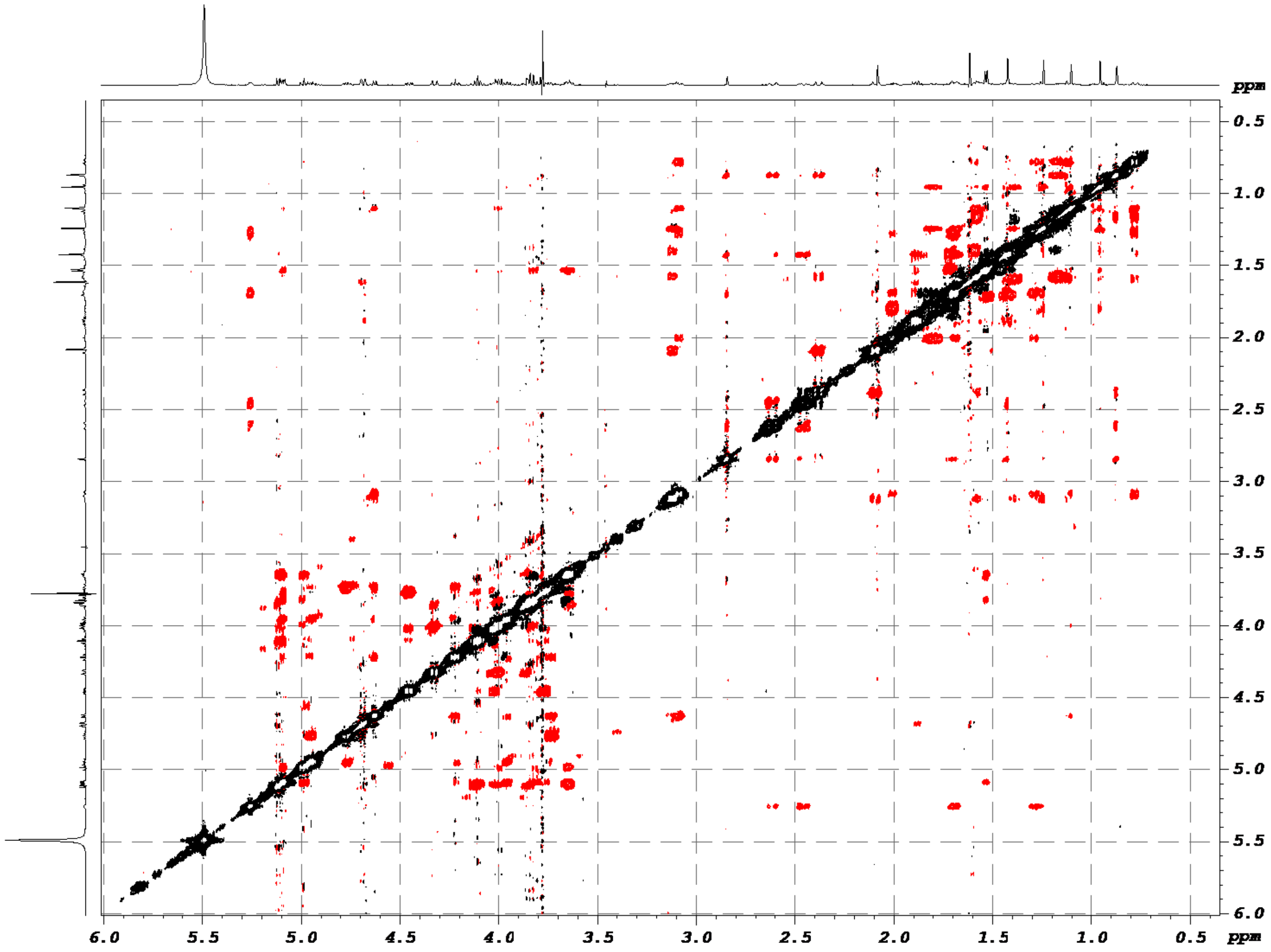

Fig. 30. The ROESY (700.00 MHz) spectrum of quadrangularisoside B$_2$ (**5**) in C_5D_5N/D_2O (4/1)

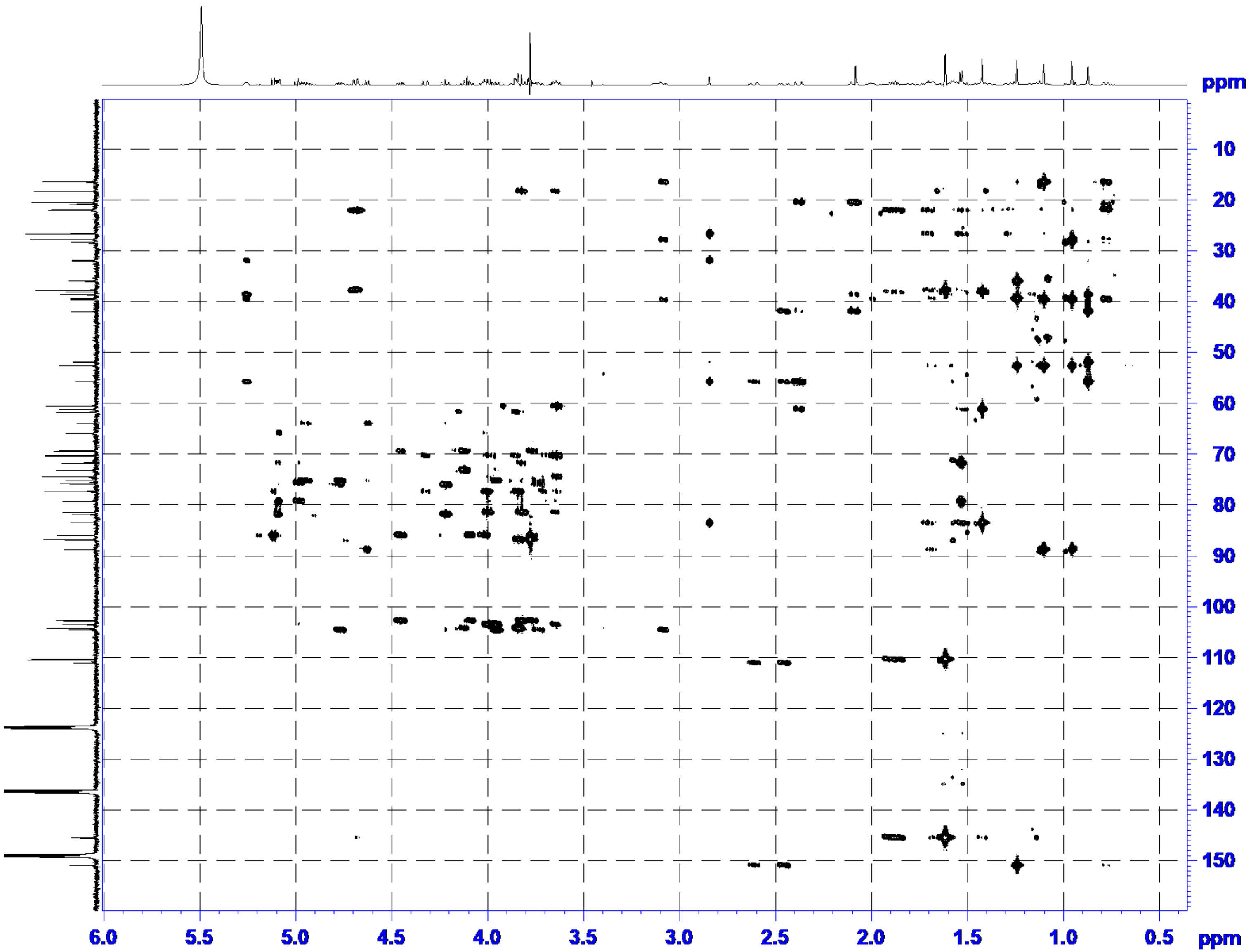

Fig. 31. The HMBC (700.00 MHz) spectrum of quadrangularisoside B2 (**5**) in C_5D_5N/D_2O (4/1)

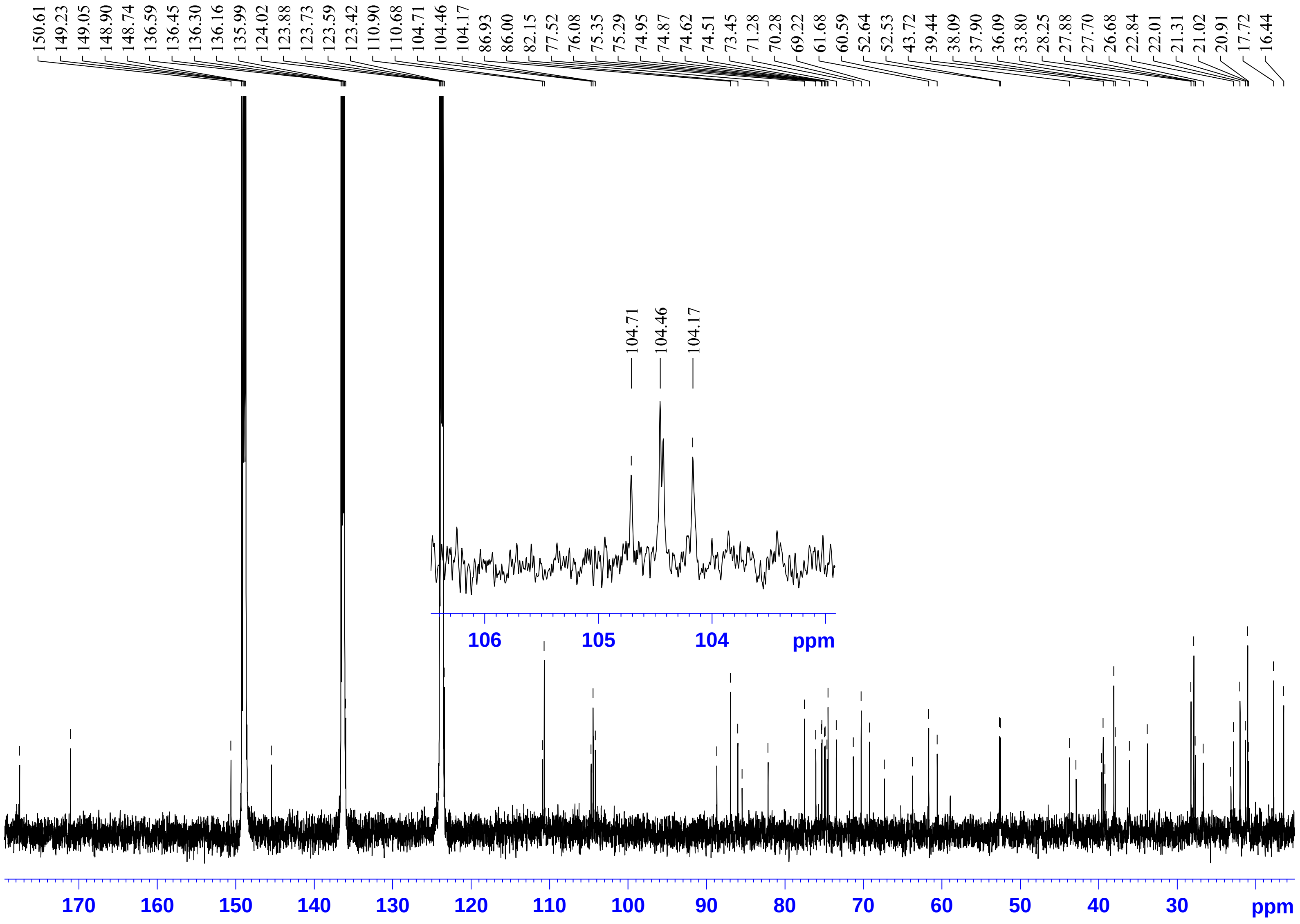

Fig. 32. The ^{13}C NMR (176.03 MHz) spectrum of quadrangularisoside C (**6**) in C_5D_5N/D_2O (4/1)

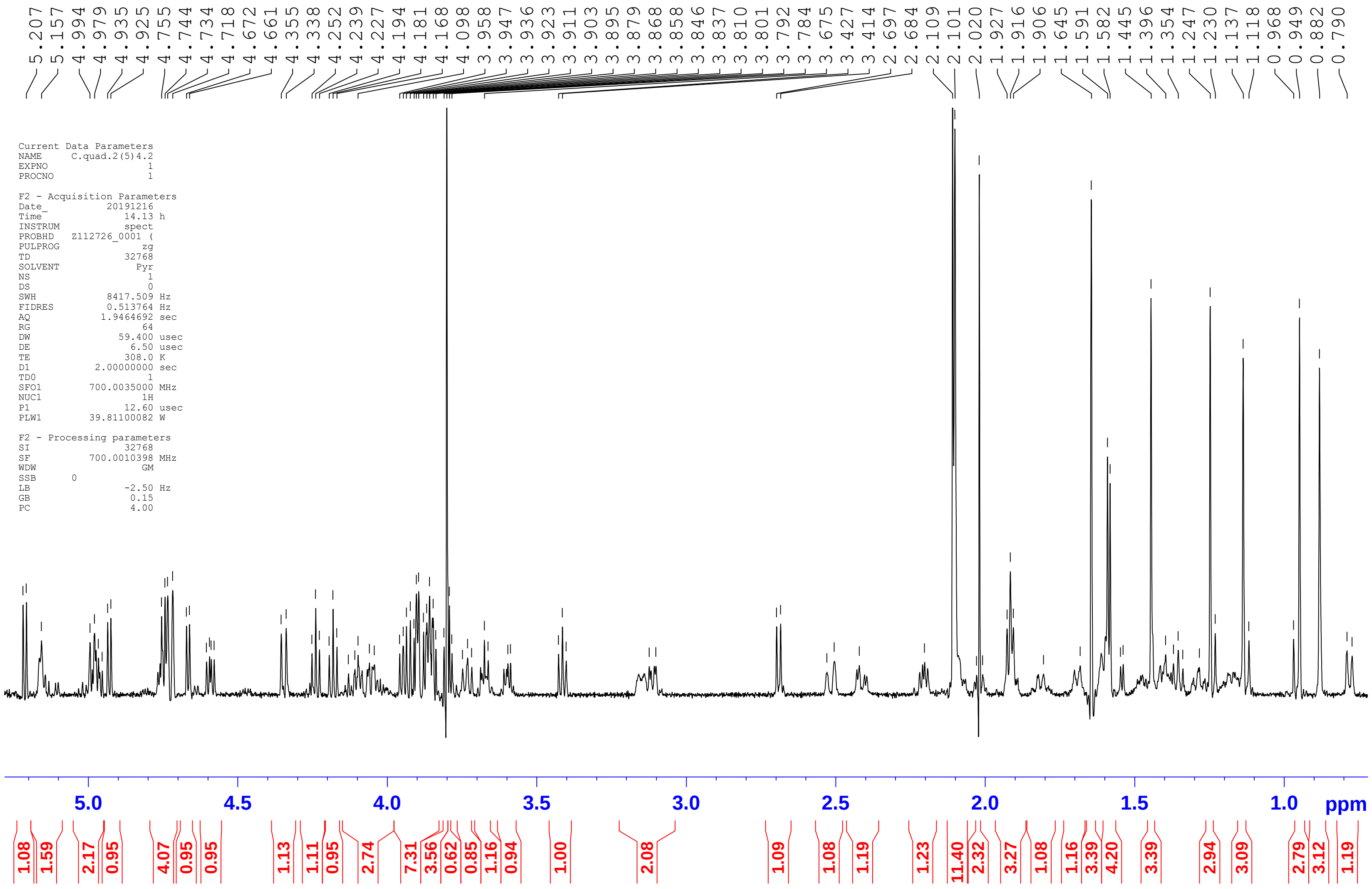

Fig. 33. The ^{1}H NMR (700.00 MHz) spectrum of quadrangularisoside C (**6**) in C_5D_5N/D_2O (4/1)

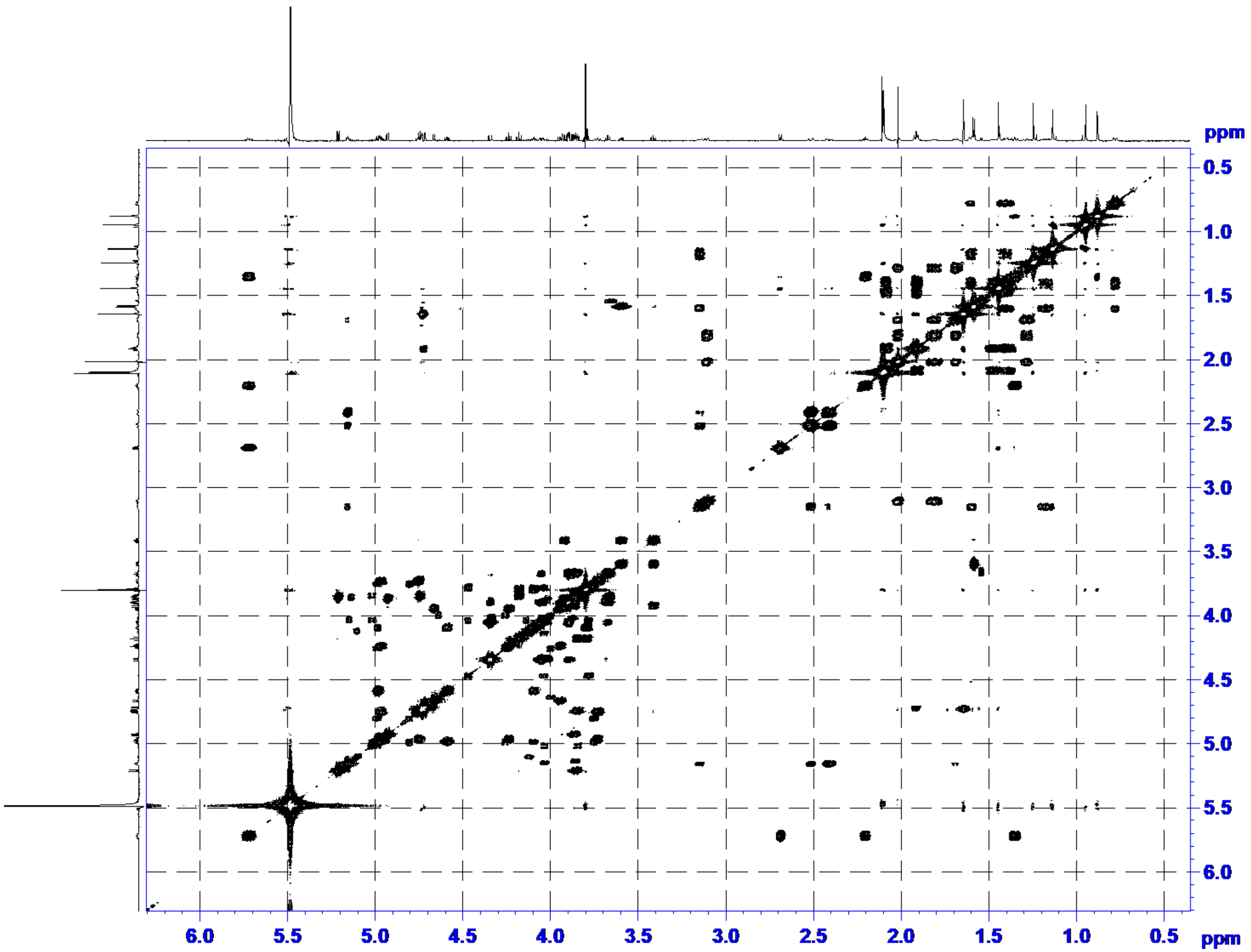

Fig. 34. The COSY (700.00 MHz) spectrum of quadrangularisoside C (**6**) in C_5D_5N/D_2O (4/1)

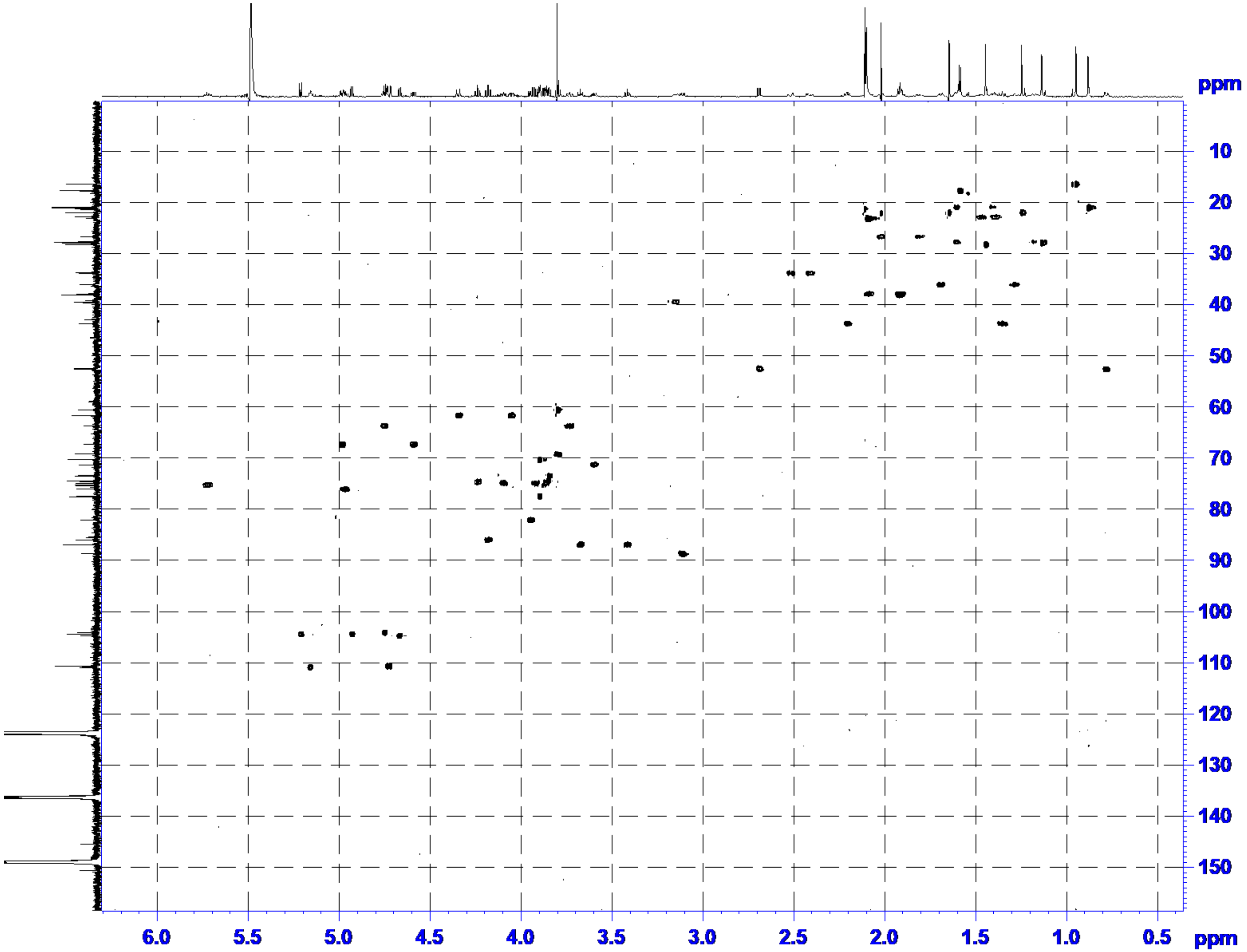

Fig. 35. The HSQC (700.00 MHz) spectrum of quadrangularisoside C (**6**) in C_5D_5N/D_2O (4/1)

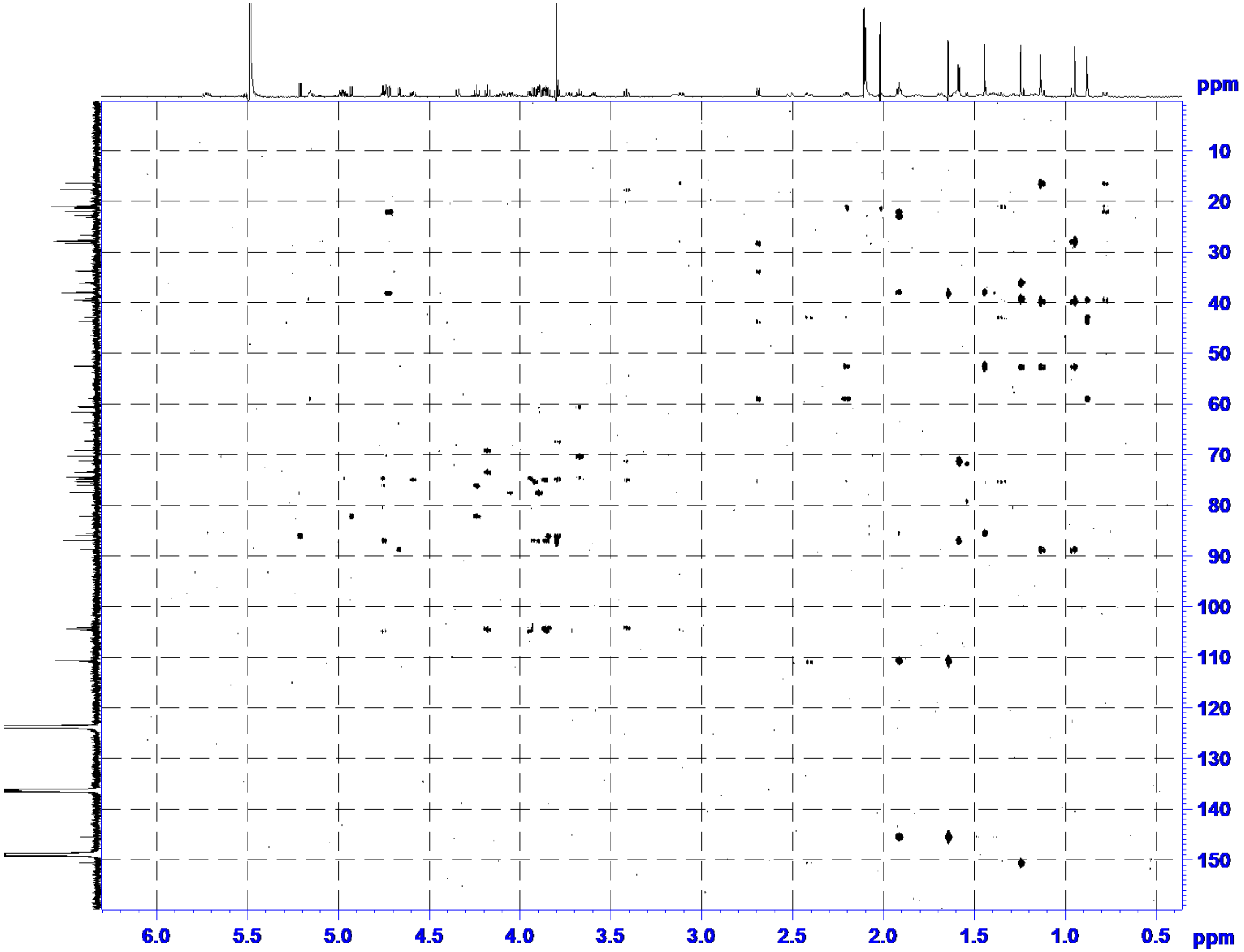

Fig. 36. The HMBC (700.00 MHz) spectrum of quadrangularisoside C (**6**) in C_5D_5N/D_2O (4/1)

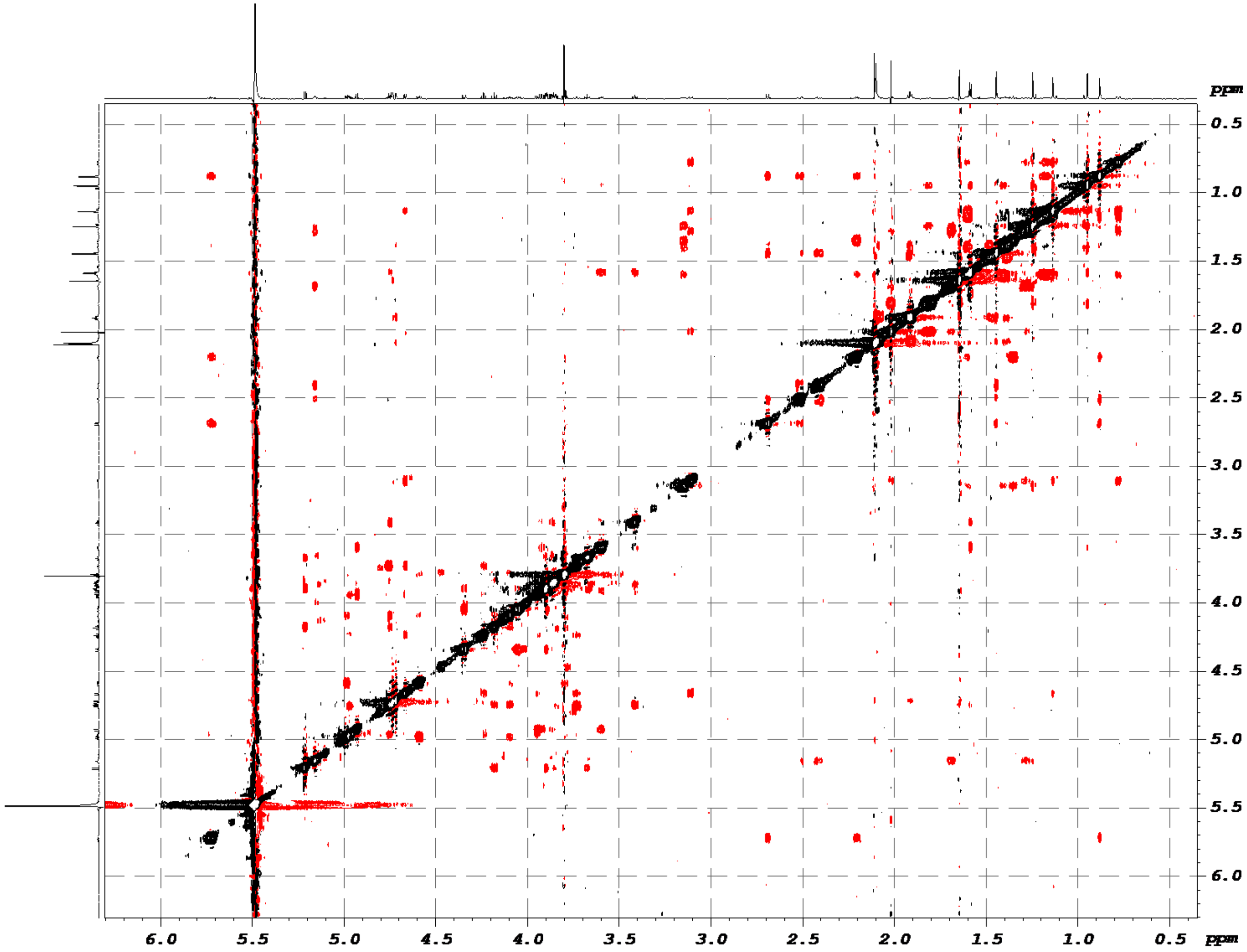

Fig. 37. The ROESY (700.00 MHz) spectrum of quadrangularisoside C (**6**) in C_5D_5N/D_2O (4/1)

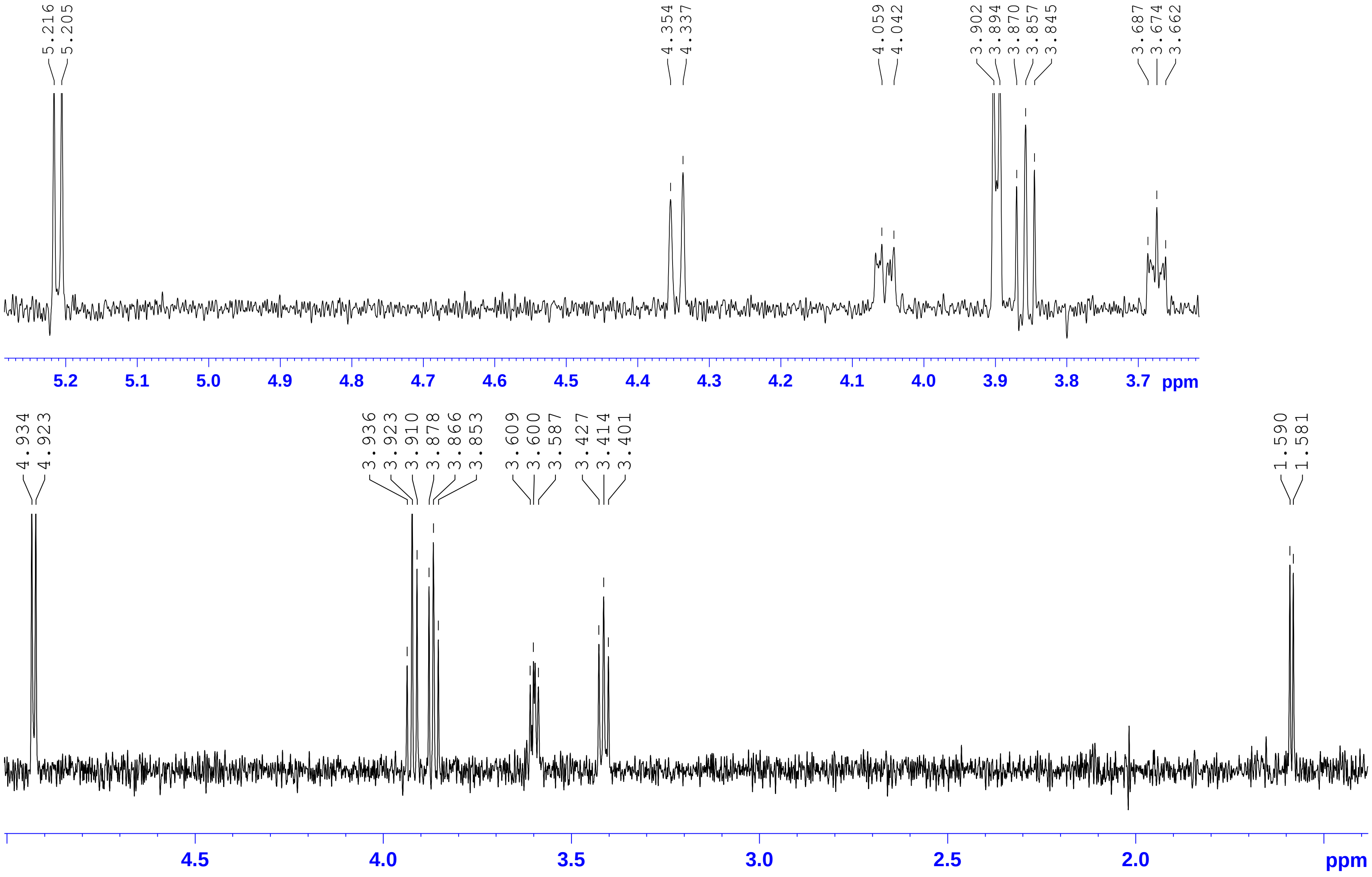

Fig. 38. 1D TOCSY (700.00 MHz) spectra of quadrangularisoside C (**6**) in C_5D_5N/D_2O (4/1)

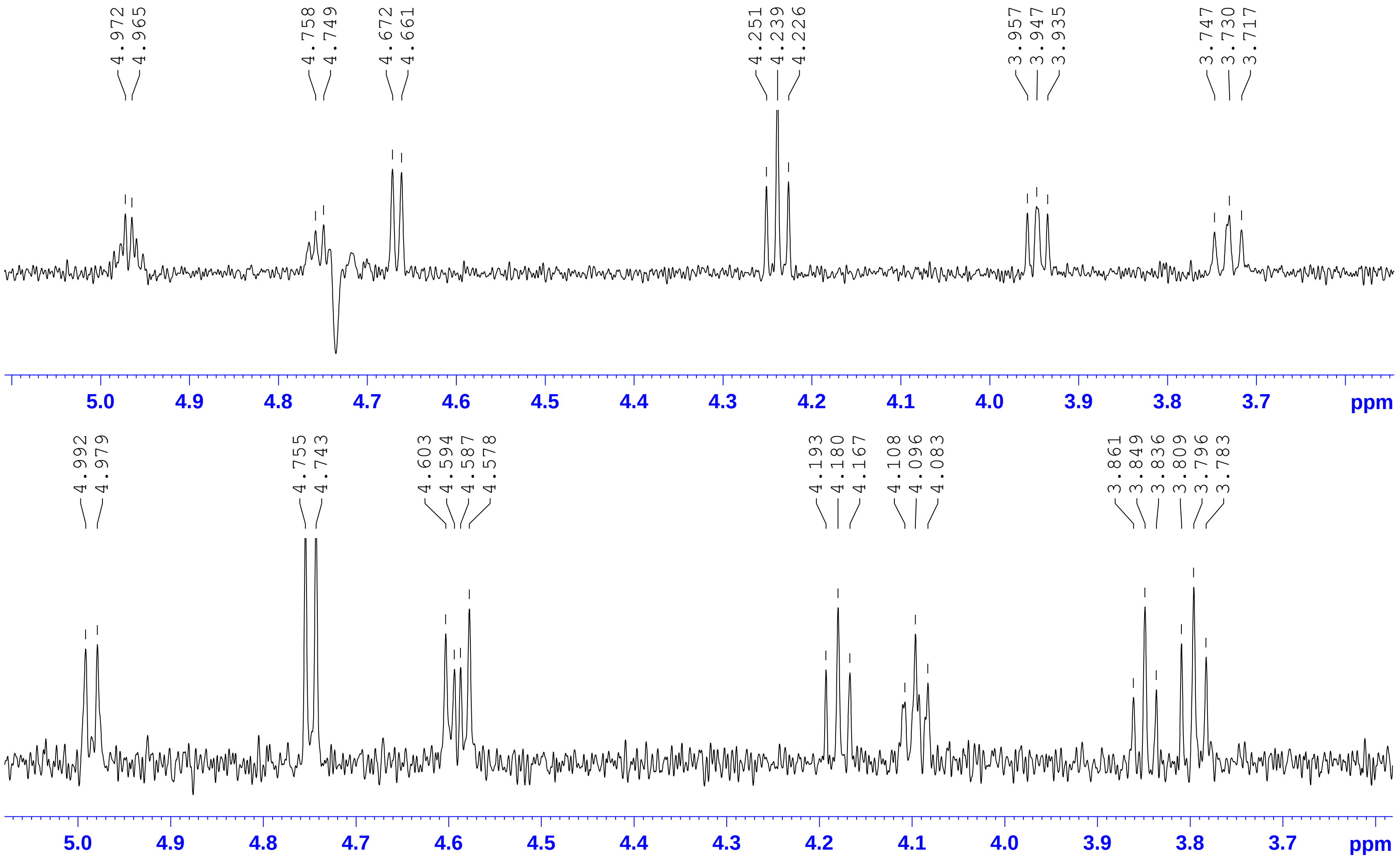

Fig. 39. 1D TOCSY (700.00 MHz) spectra of quadrangularisoside C (**6**) in C_5D_5N/D_2O (4/1)

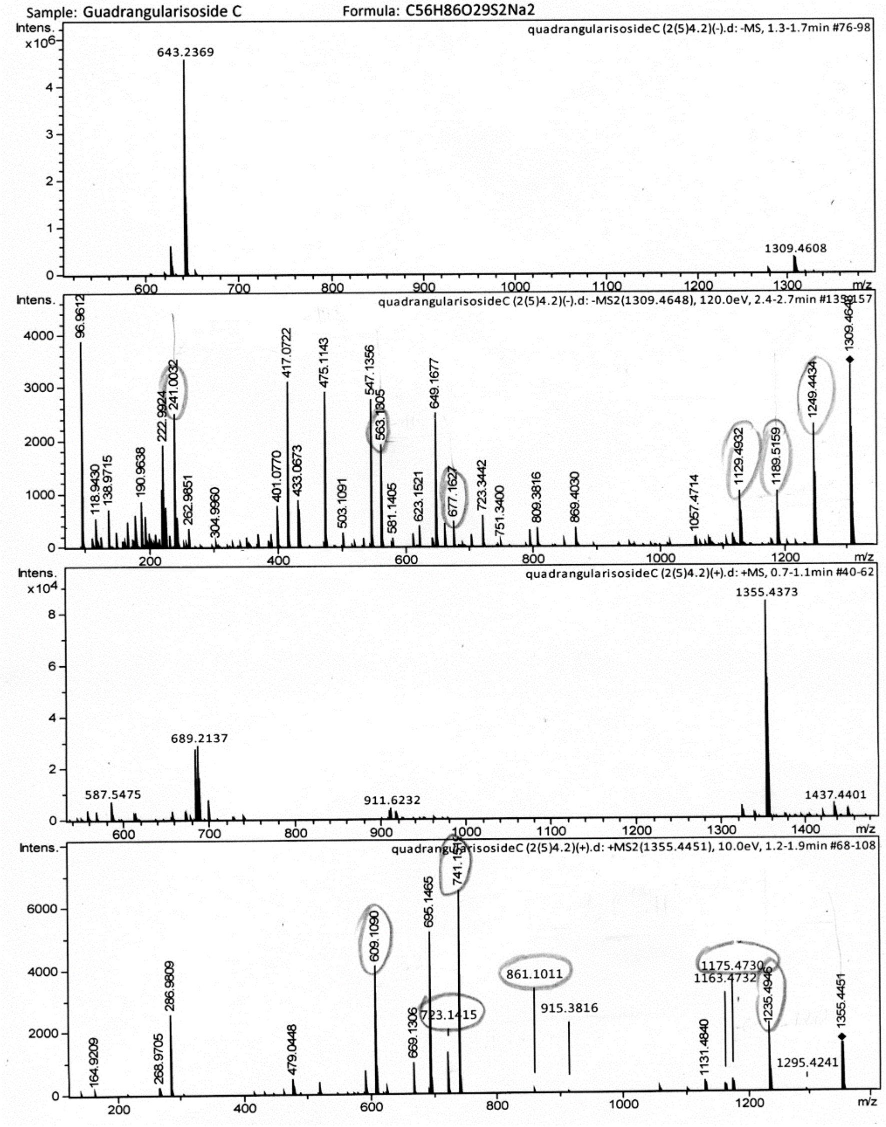

Fig. 40. HR-ESI-MS and ESI-MS/MS spectra of quadrangularisoside C (**6**)

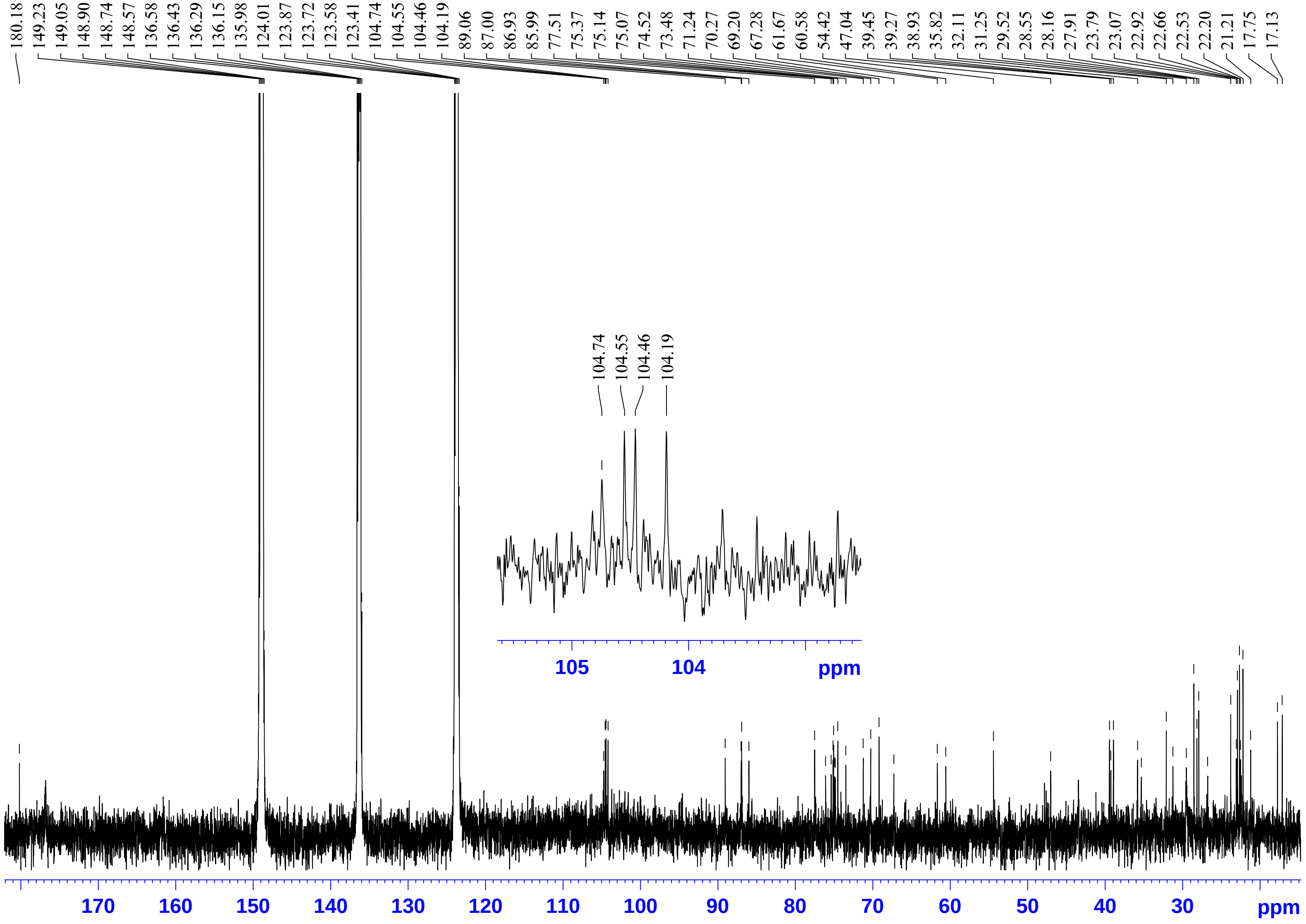

Fig. 41. The ^{13}C NMR (176.03 MHz) spectrum of quadrangularisoside C_1 (**7**) in C_5D_5N/D_2O (4/1)

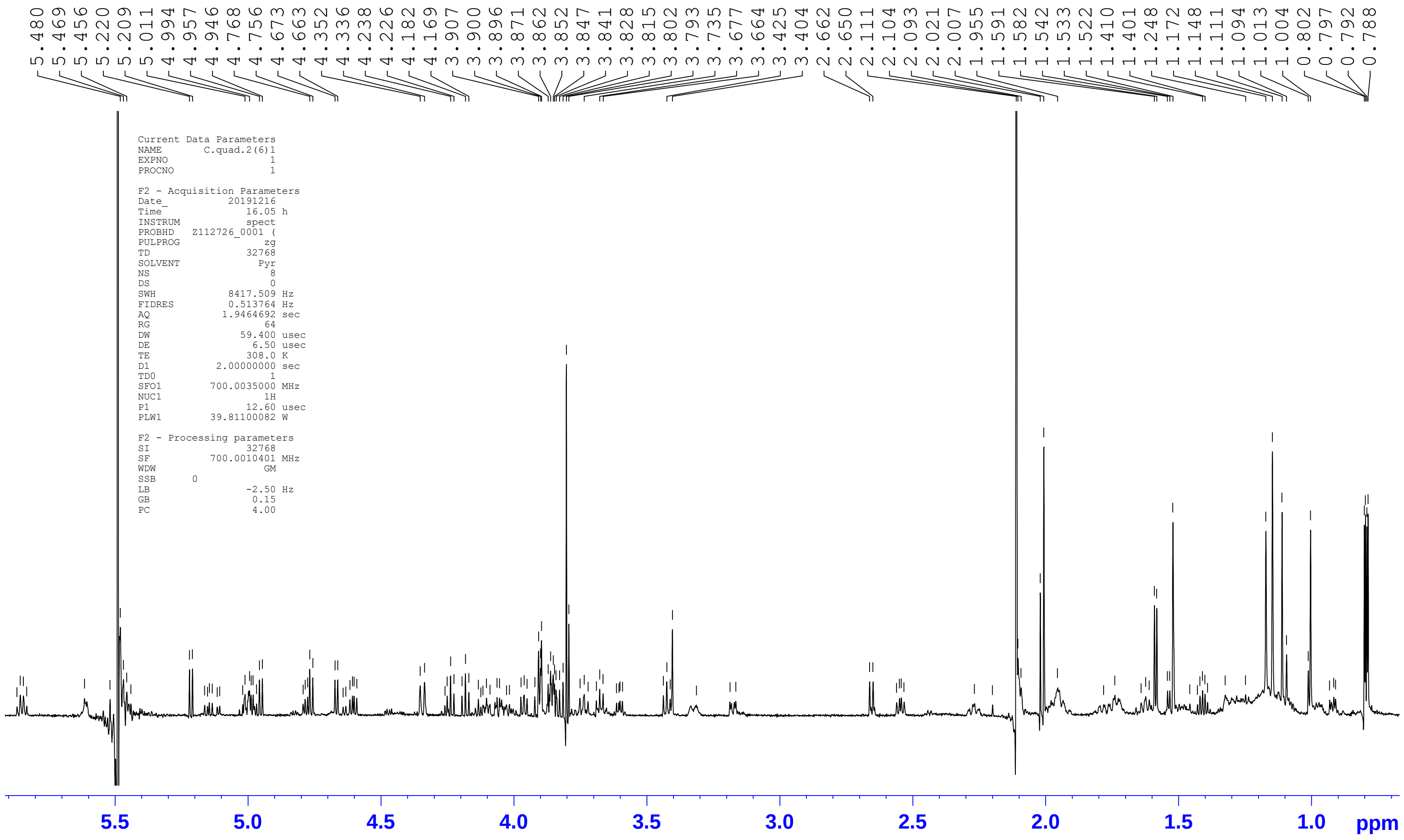

Fig. 42. The ^{1}H NMR (700.00 MHz) spectrum of quadrangularisoside C_1 (**7**) in C_5D_5N/D_2O (4/1)

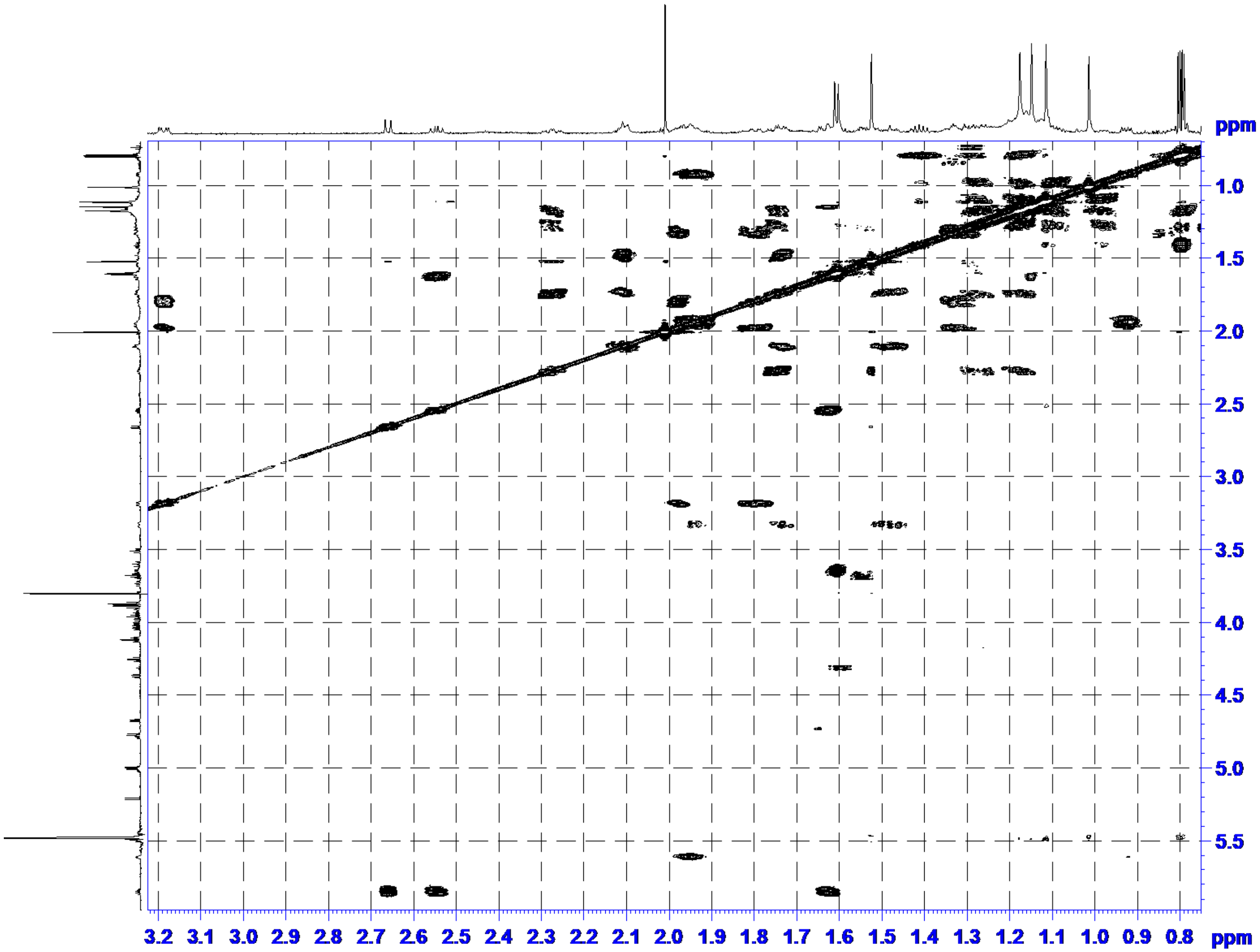

Fig. 43. The COSY (700.00 MHz) spectrum of the aglycone part of quadrangularisoside C_1 (**7**) in C_5D_5N/D_2O (4/1)

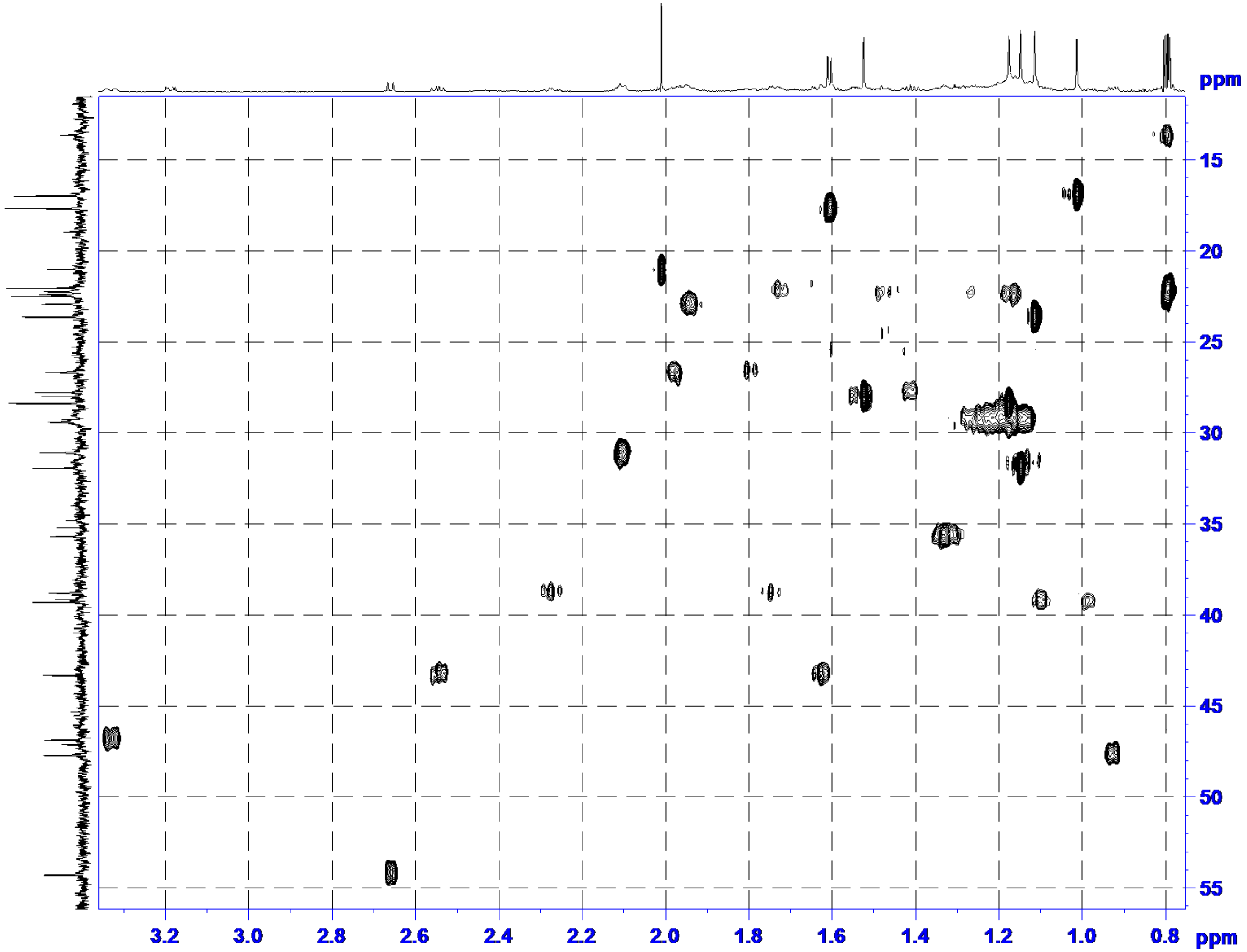

Fig. 44. The HSQC (700.00 MHz) spectrum of the aglycone part of quadrangularisoside C_1 (**7**) in C_5D_5N/D_2O (4/1)

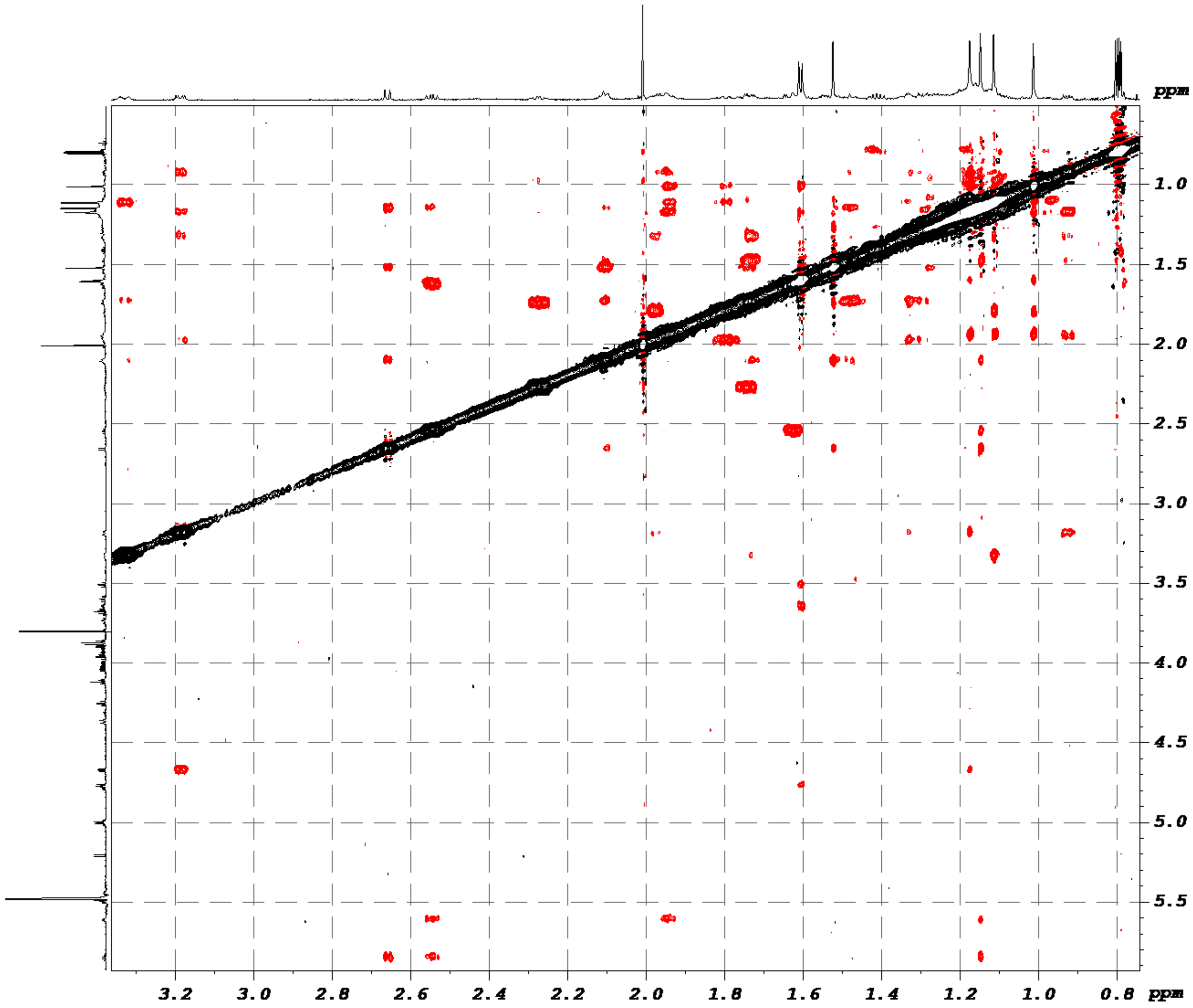

Fig. 45. The ROESY (700.00 MHz) spectrum of the aglycone part of quadrangularisoside C_1 (**7**) in C_5D_5N/D_2O (4/1)

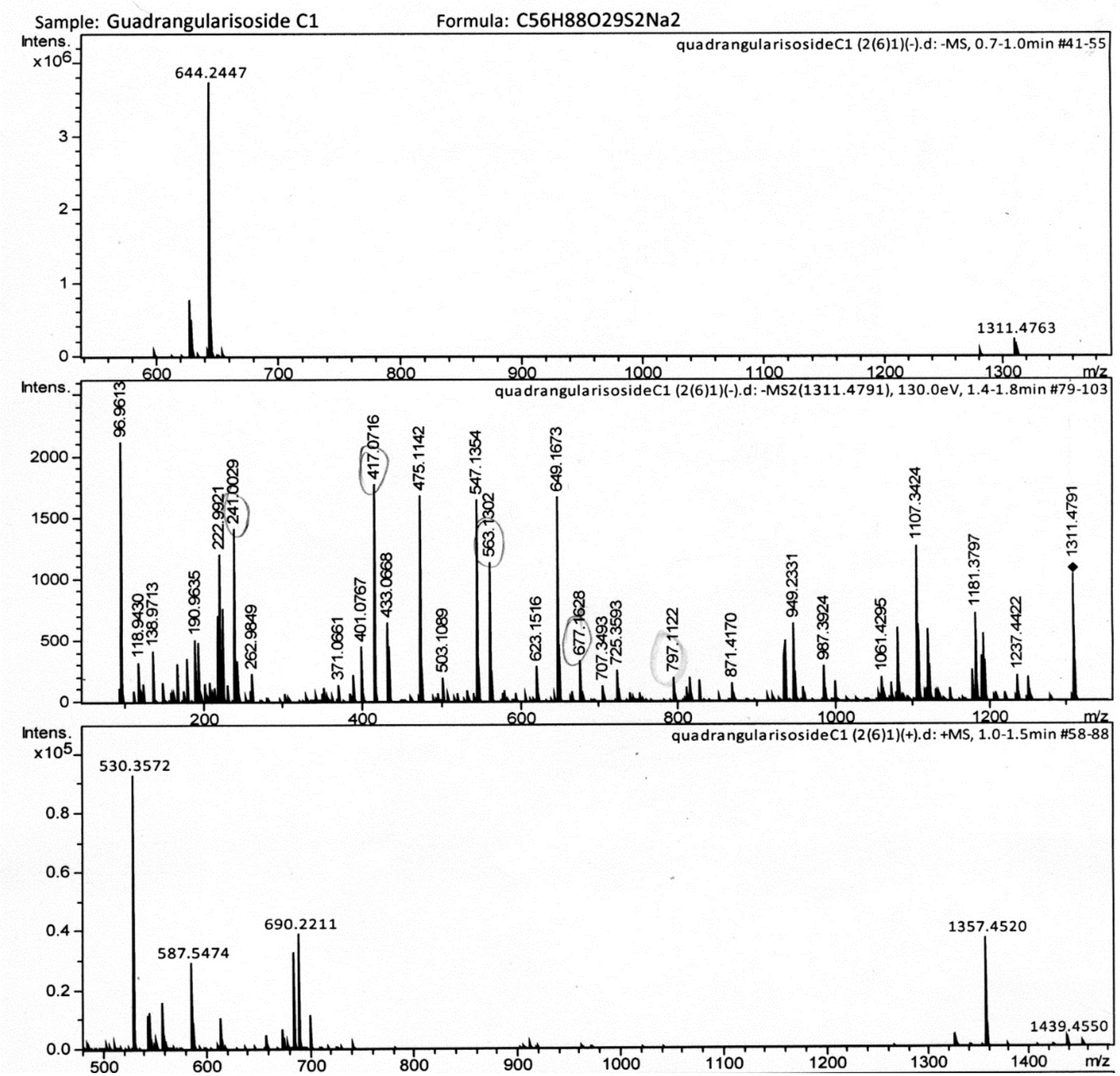

Fig. 46. HR-ESI-MS and ESI-MS/MS spectra of quadrangularisoside C_1 (**7**)

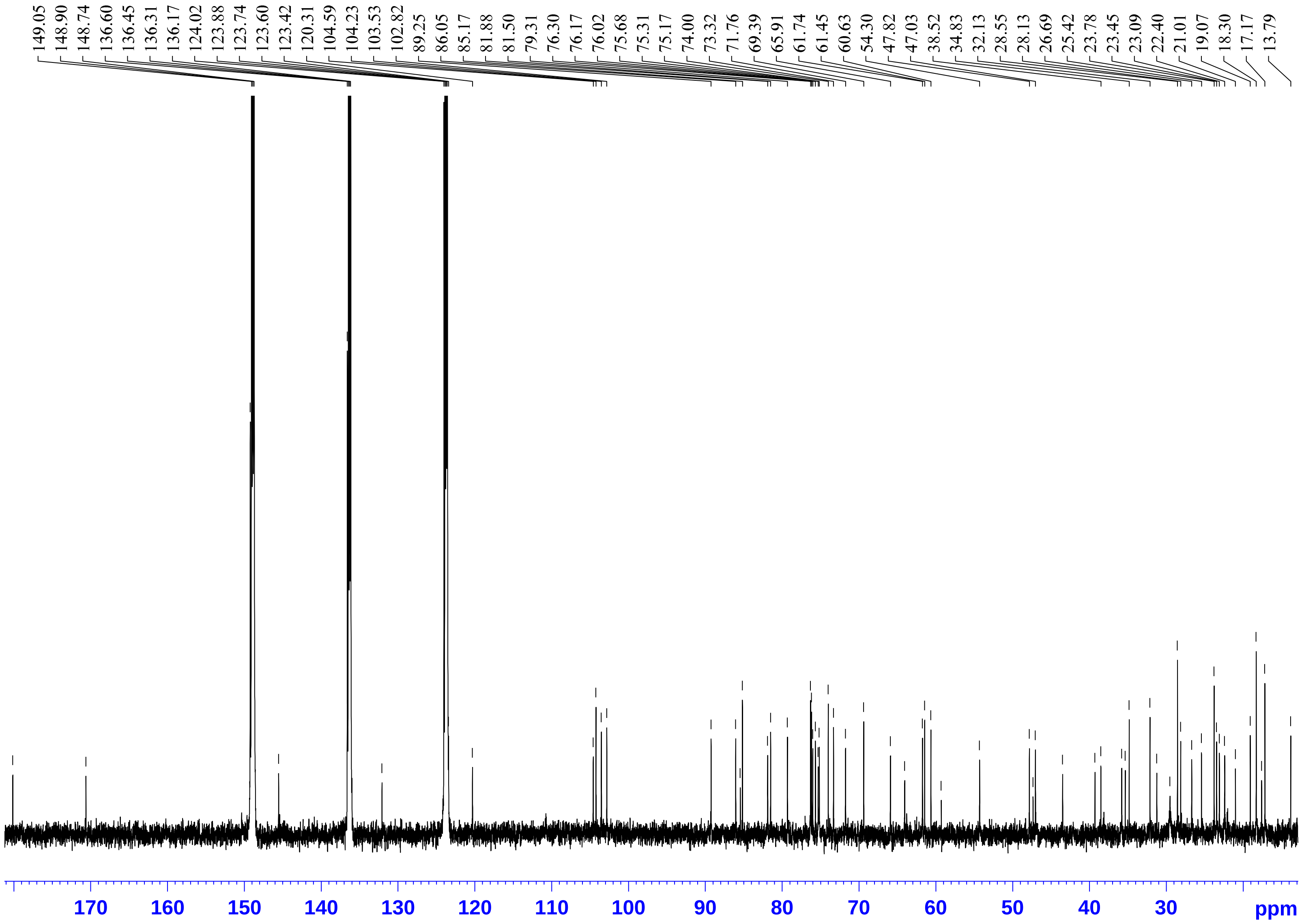

Fig. 47. The ^{13}C NMR (176.03 MHz) spectrum of quadrangularisoside D (**8**) in C_5D_5N/D_2O (4/1)

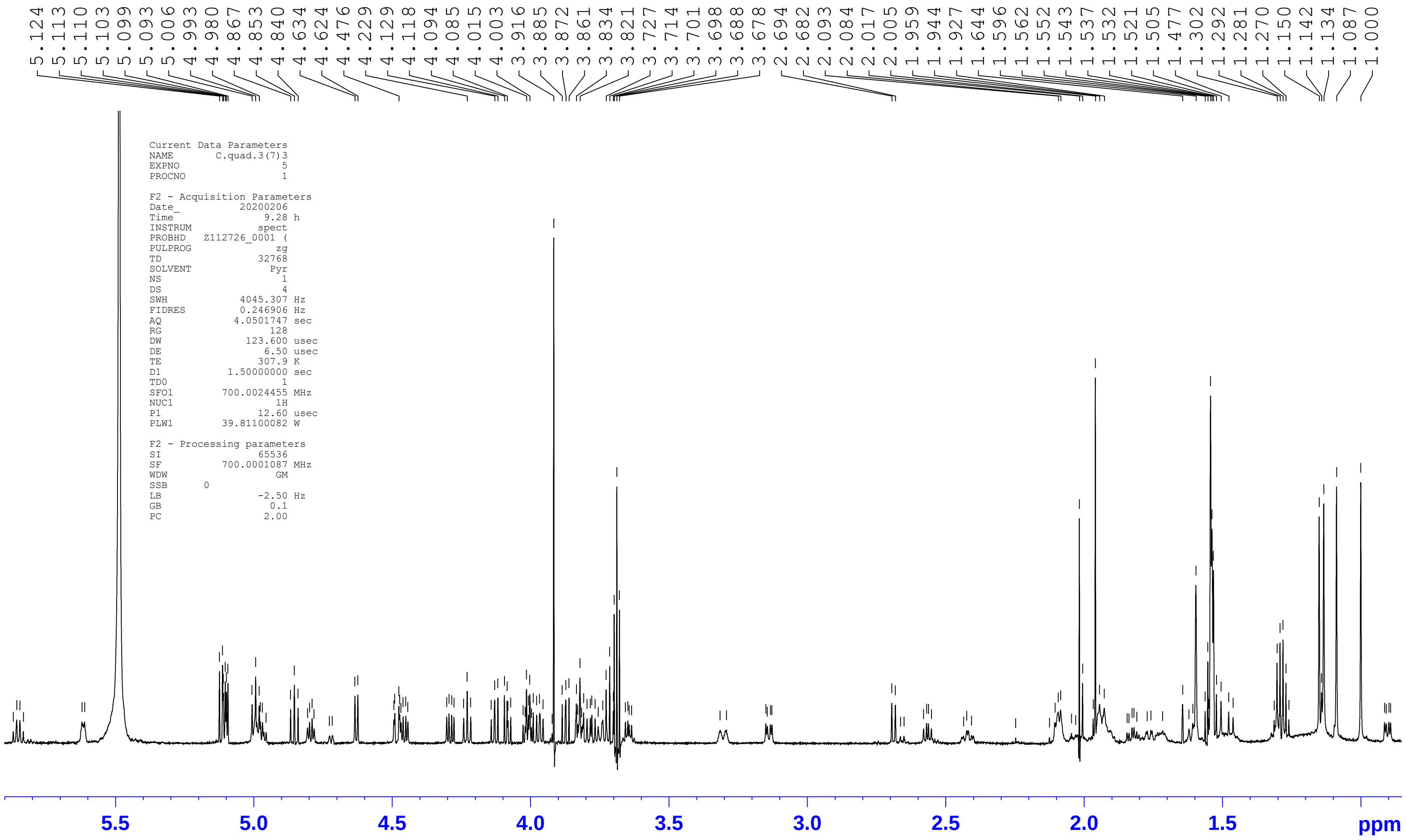

Fig. 48. The ^{1}H NMR (700.00 MHz) spectrum of quadrangularisoside D (**8**) in C_5D_5N/D_2O (4/1)

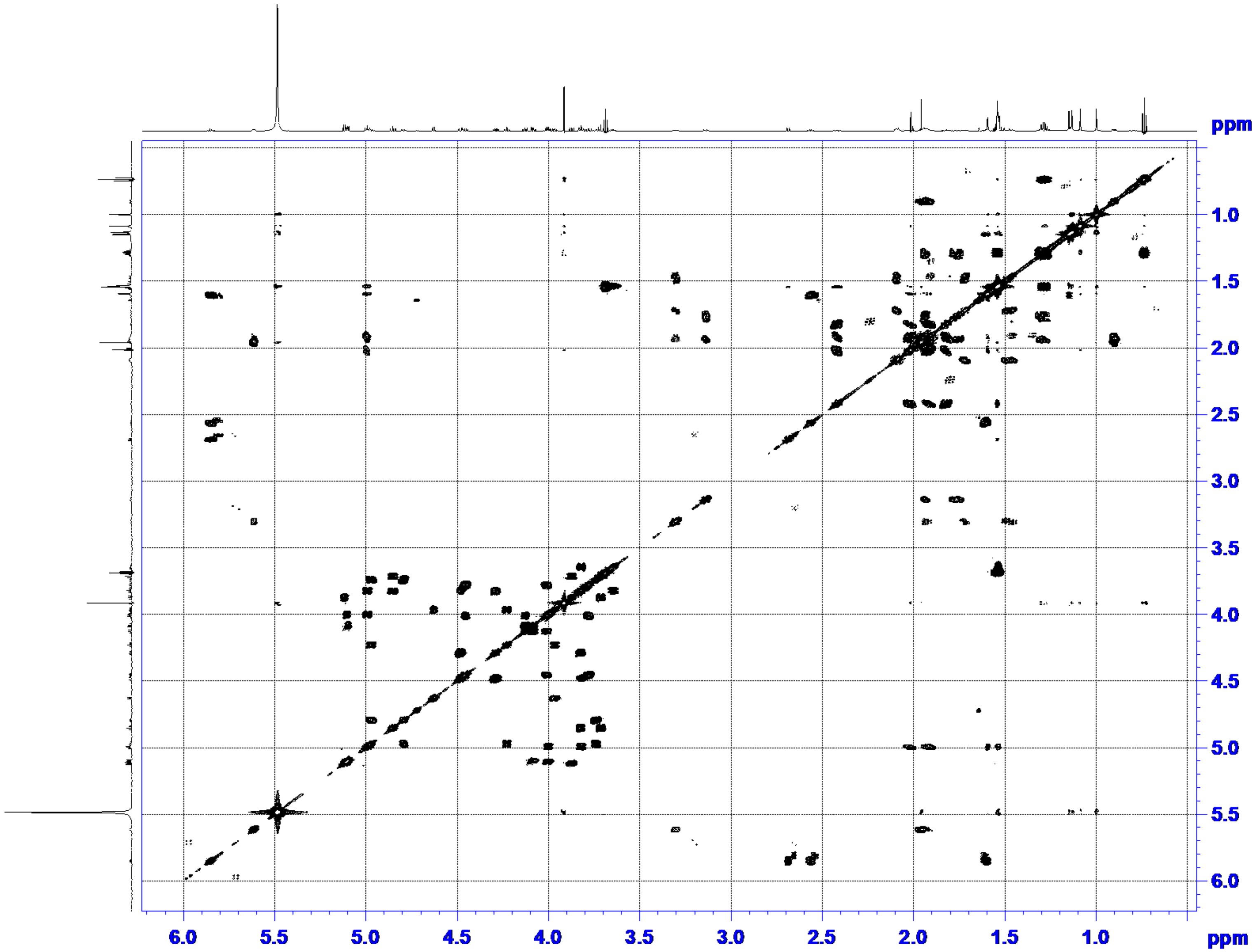

Fig. 49. The COSY (700.00 MHz) spectrum of quadrangularisoside D (**8**) in C_5D_5N/D_2O (4/1)

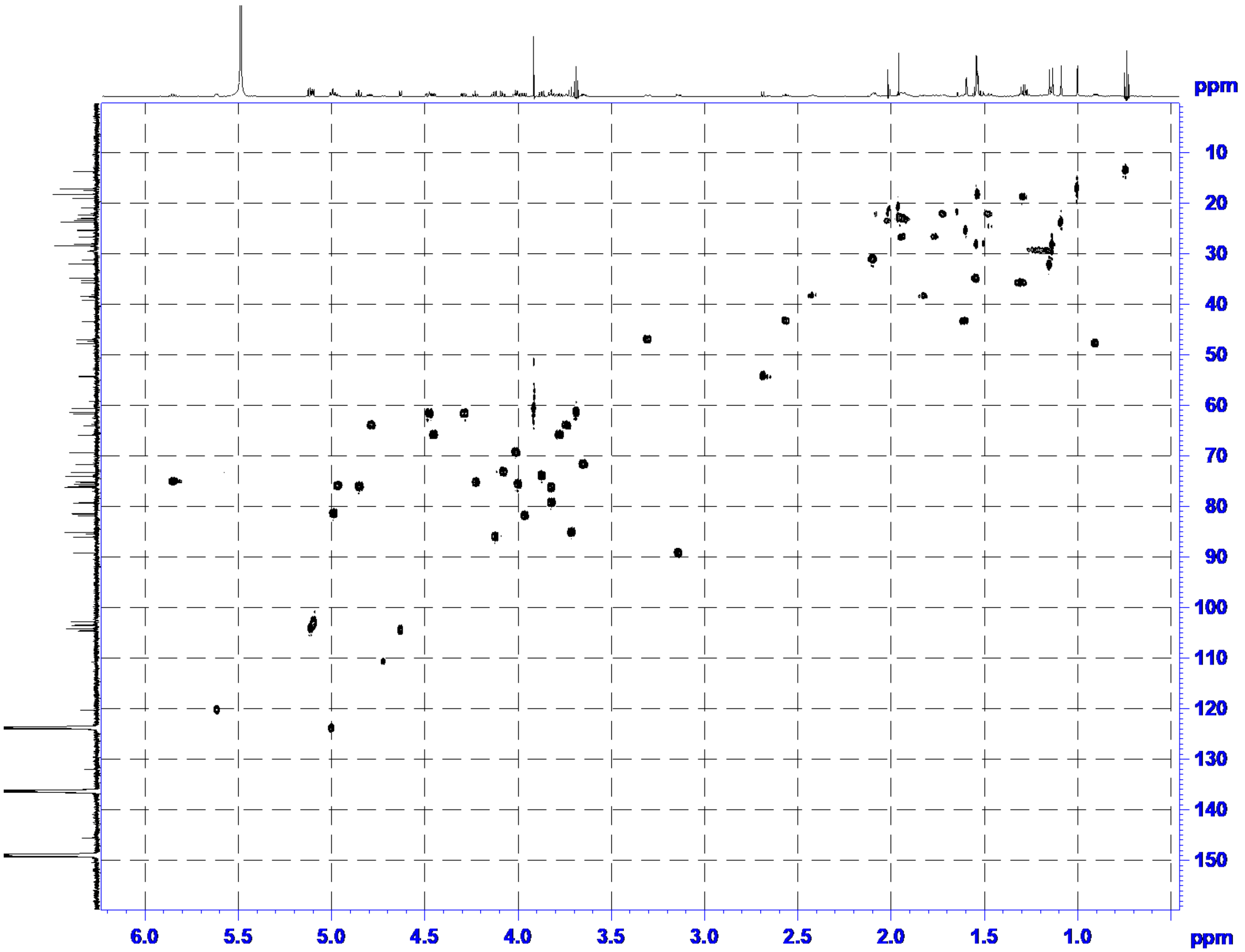

Fig. 50. The HSQC (700.00 MHz) spectrum of quadrangularisoside D (**8**) in C_5D_5N/D_2O (4/1)

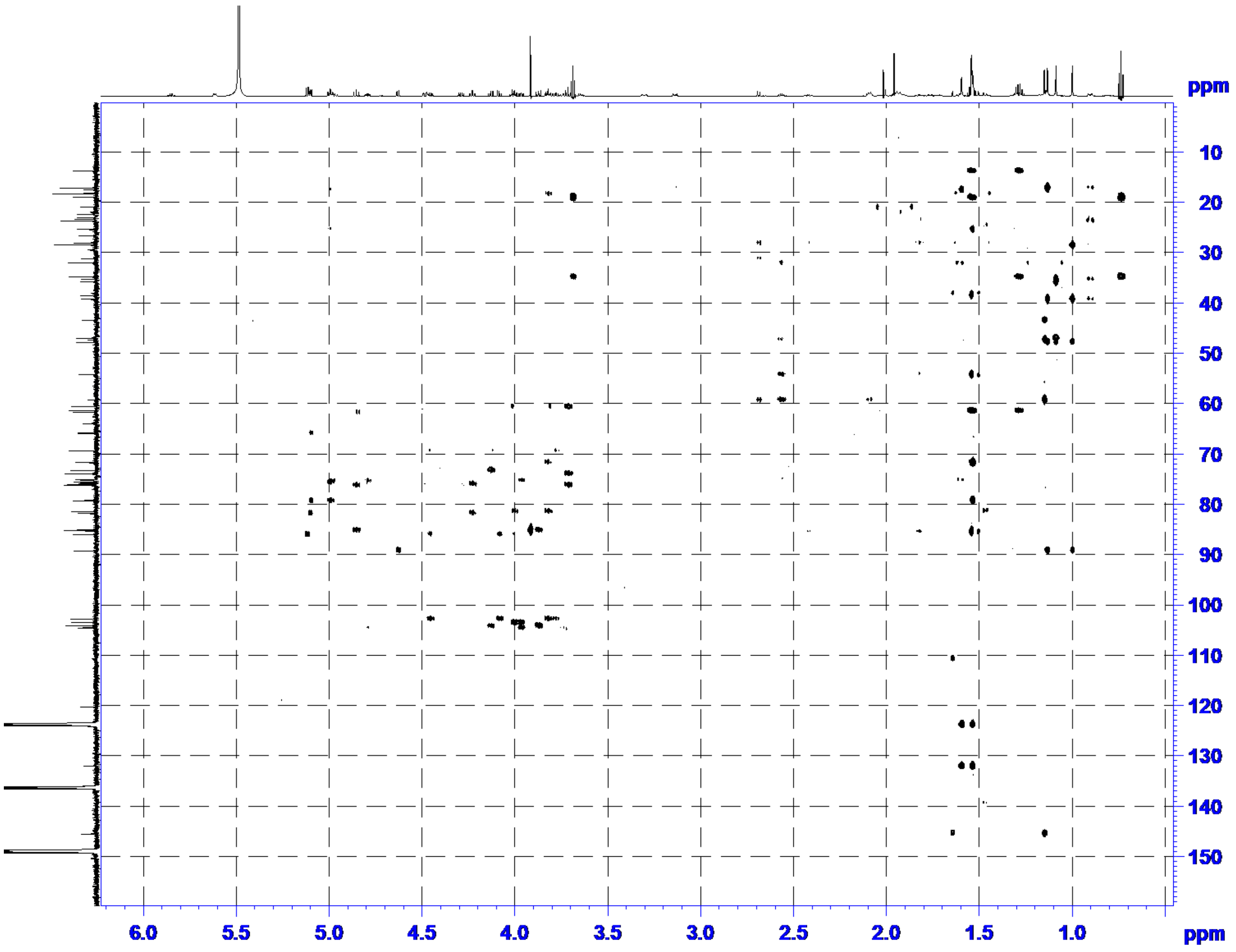

Fig. 51. The HMBC (700.00 MHz) spectrum of quadrangularisoside D (**8**) in C_5D_5N/D_2O (4/1)

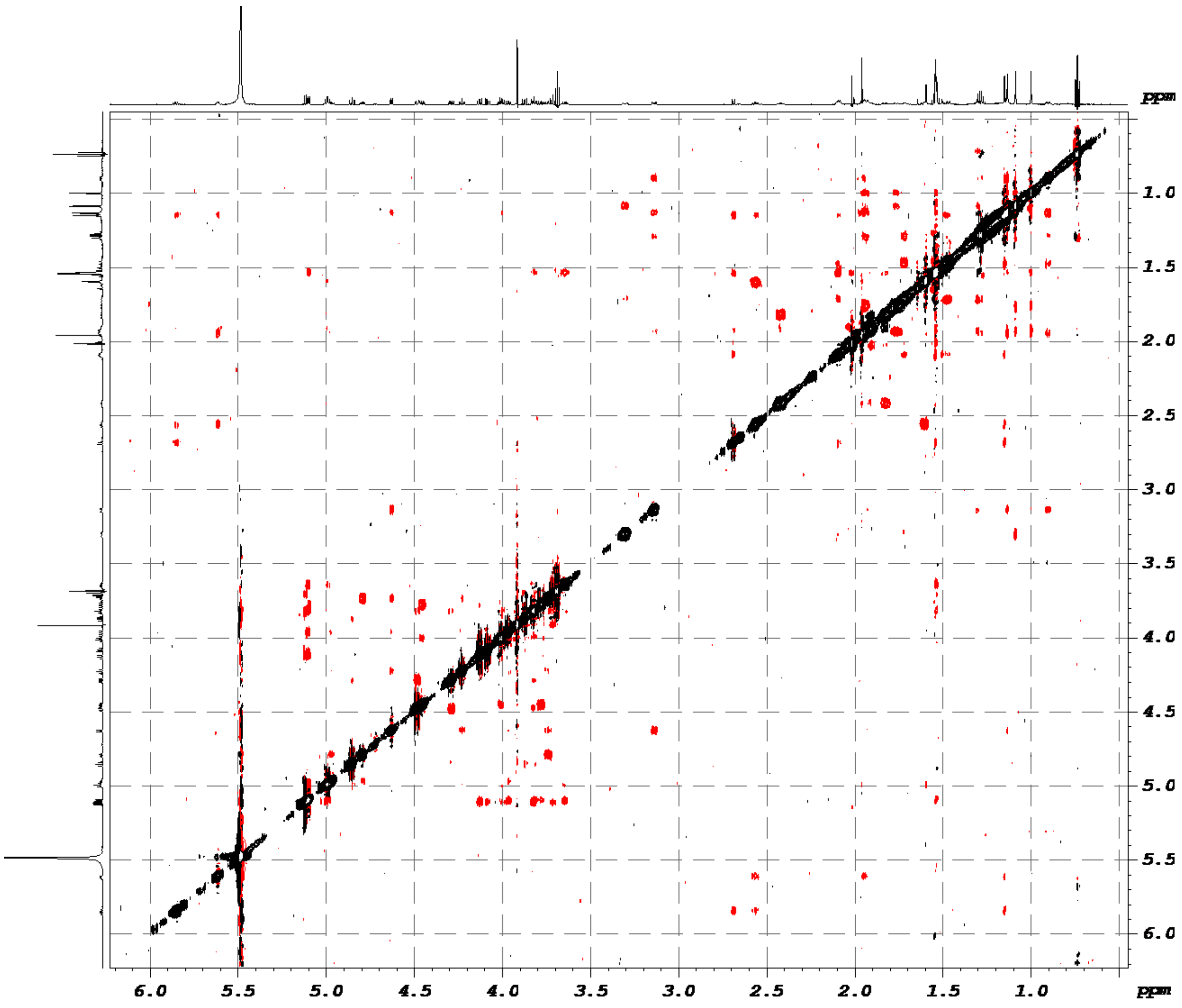

Fig. 52. The ROESY (700.00 MHz) spectrum of quadrangularisoside D (**8**) in C_5D_5N/D_2O (4/1)

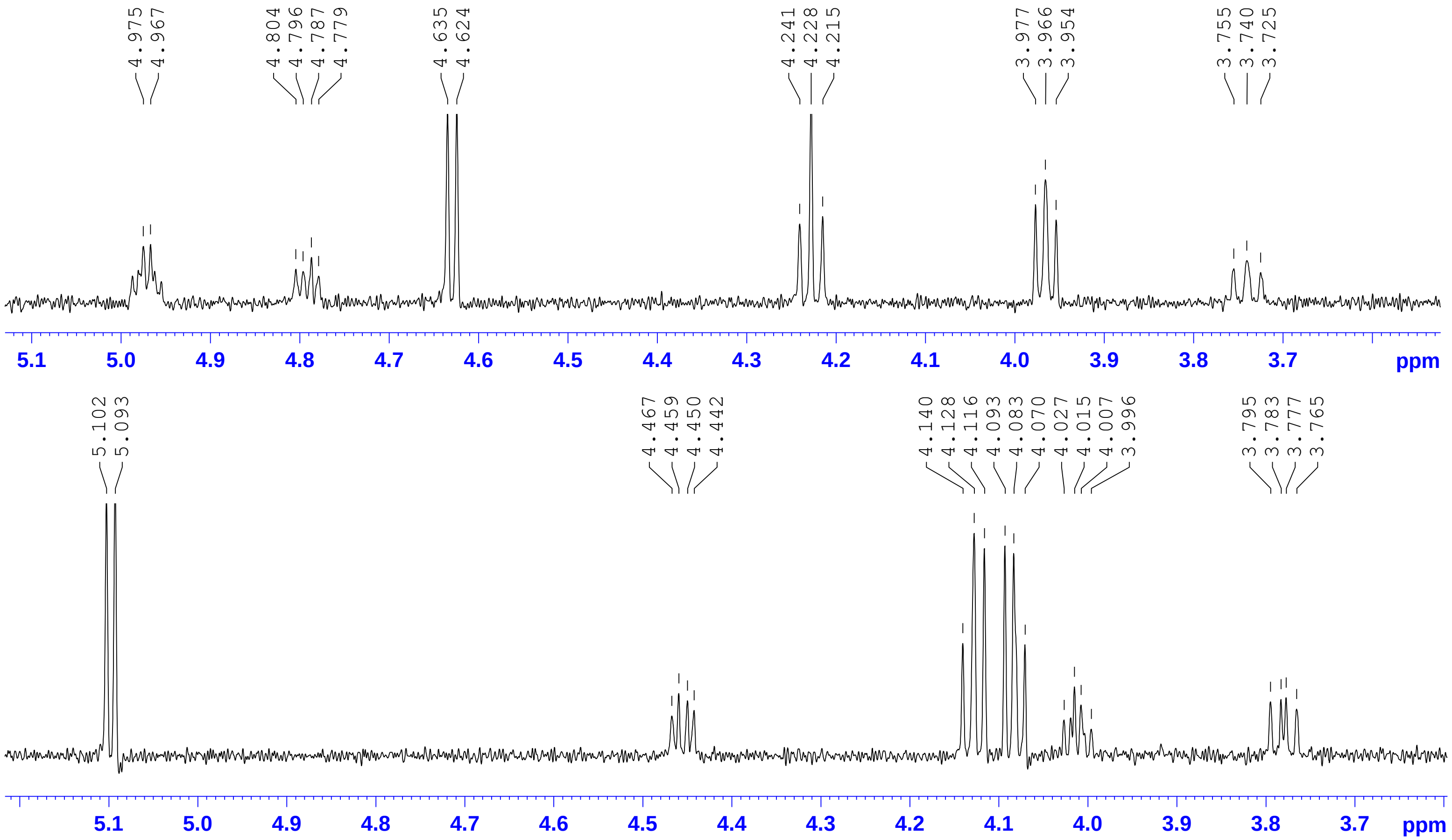

Fig. 53. 1D TOCSY (700.00 MHz) spectra of quadrangularisoside D (**8**) in C_5D_5N/D_2O (4/1)

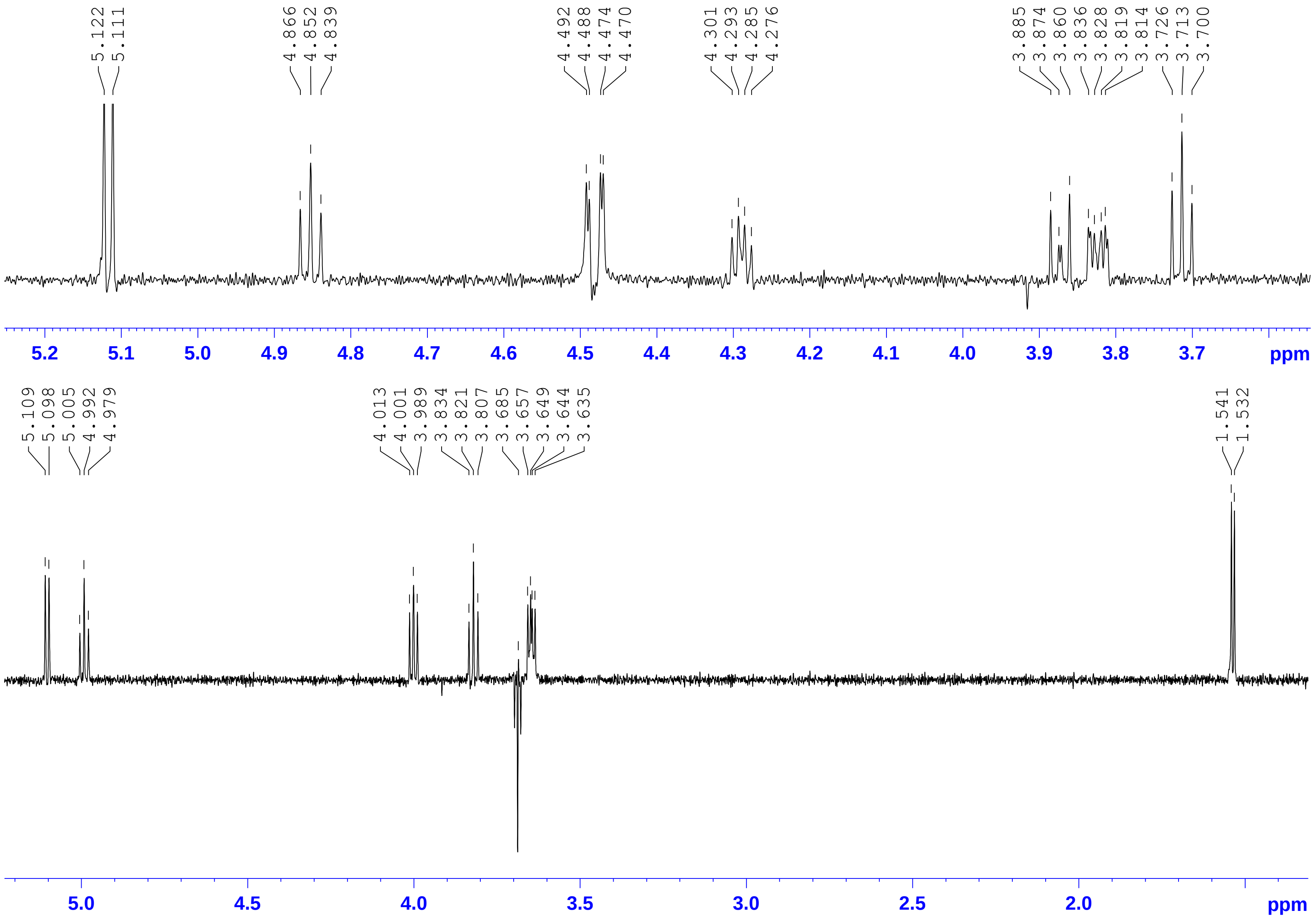

Fig. 54. 1D TOCSY (700.00 MHz) spectra of quadrangularisoside D (**8**) in C_5D_5N/D_2O (4/1)

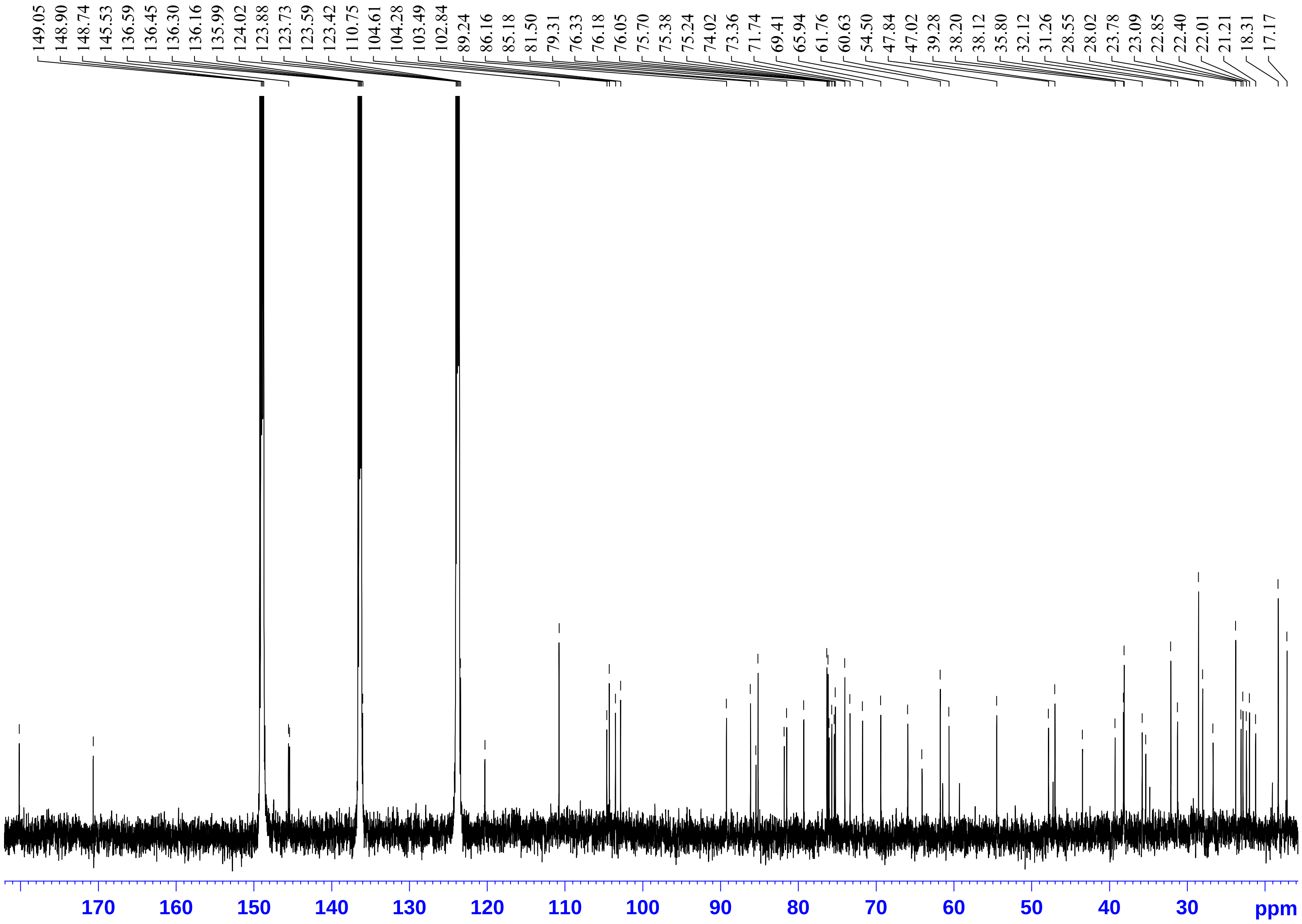

Fig. 55. The ^{13}C NMR (176.03 MHz) spectrum of quadrangularisoside D1 (**9**) in C_5D_5N/D_2O (4/1)

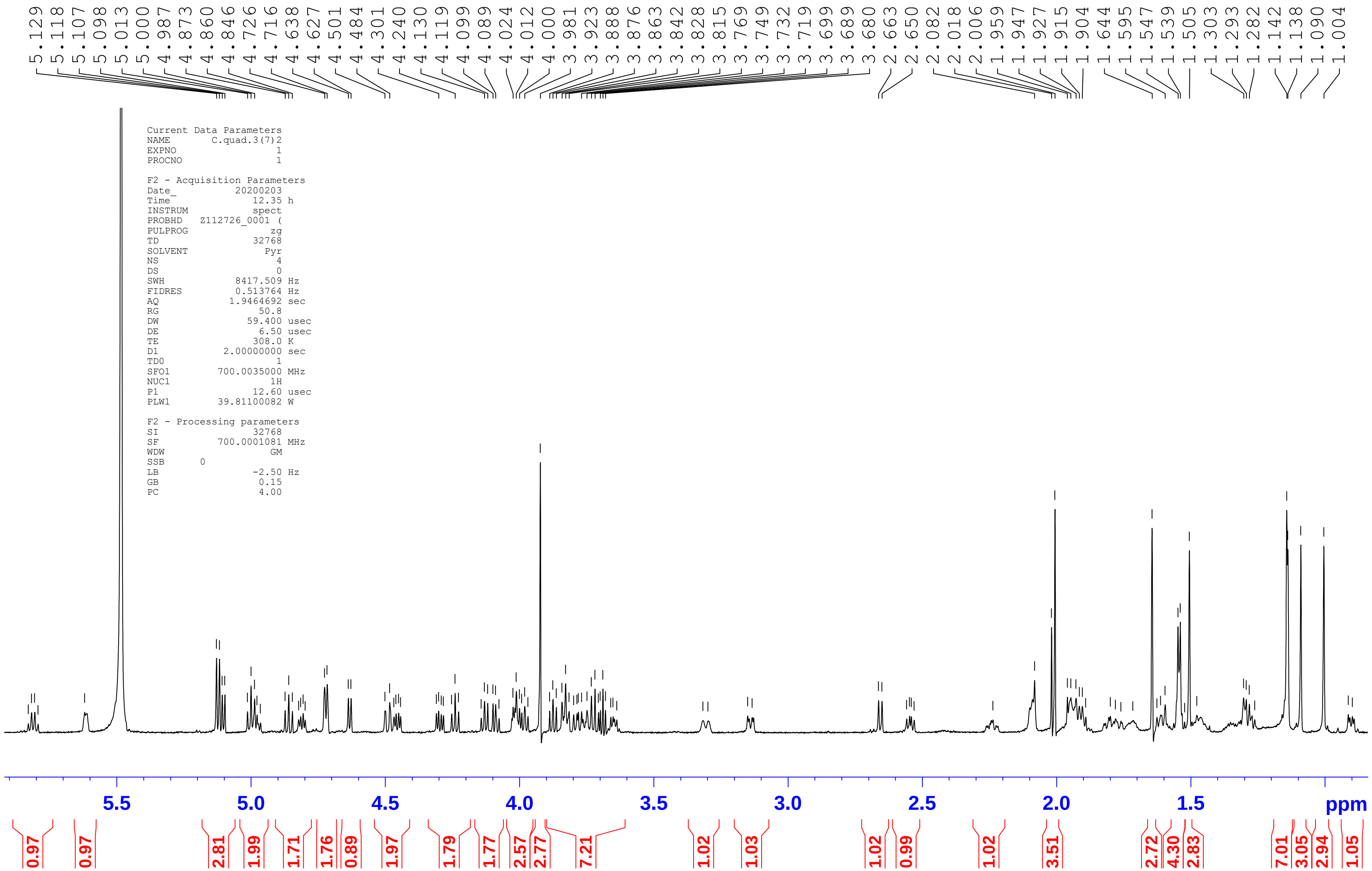

Fig. 56. The ^{1}H NMR (700.00 MHz) spectrum of quadrangularisoside D_1 (**9**) in C_5D_5N/D_2O (4/1)

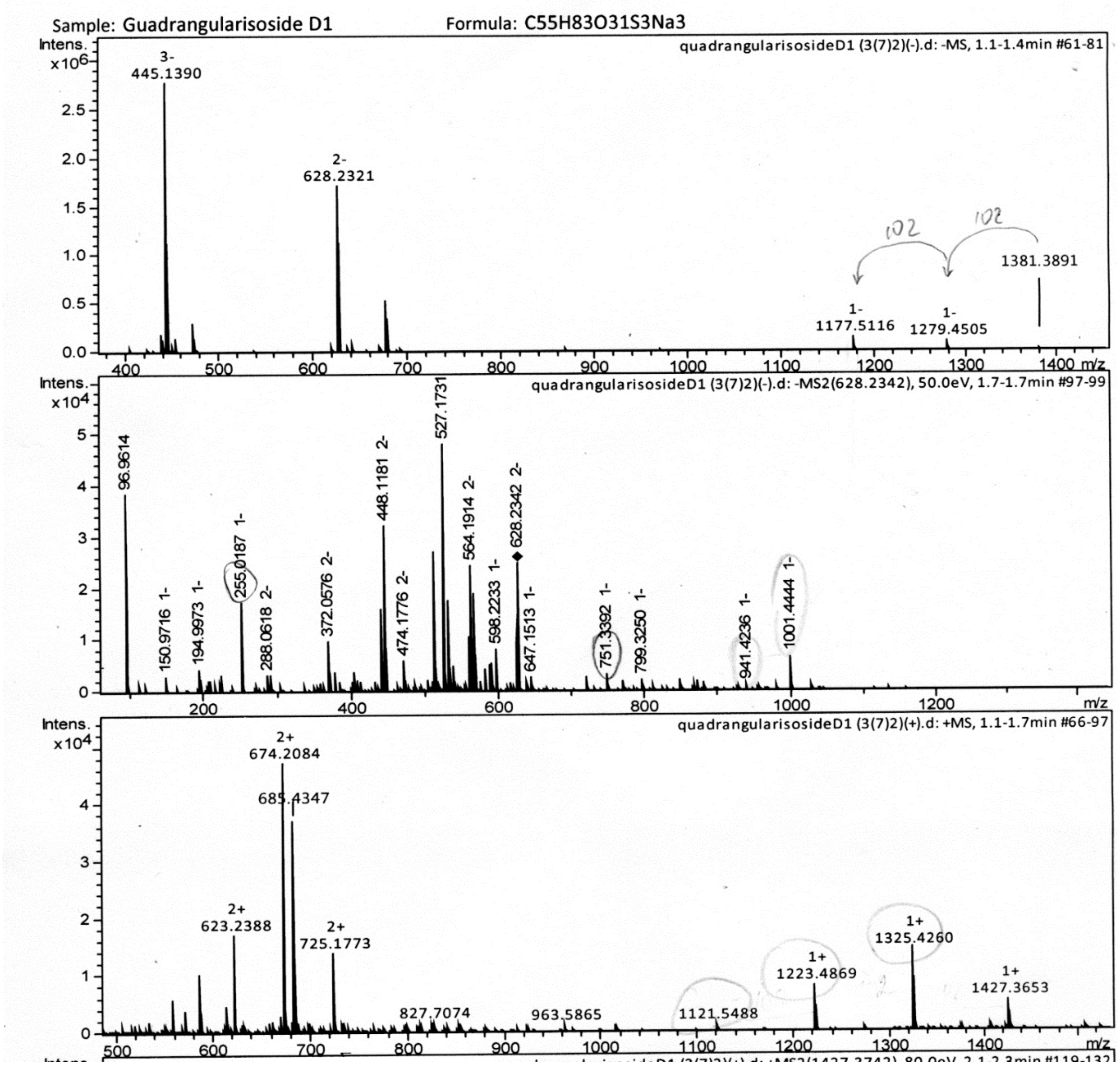

Fig. 57. HR-ESI-MS and ESI-MS/MS spectra of quadrangularisoside D_1 (**9**)

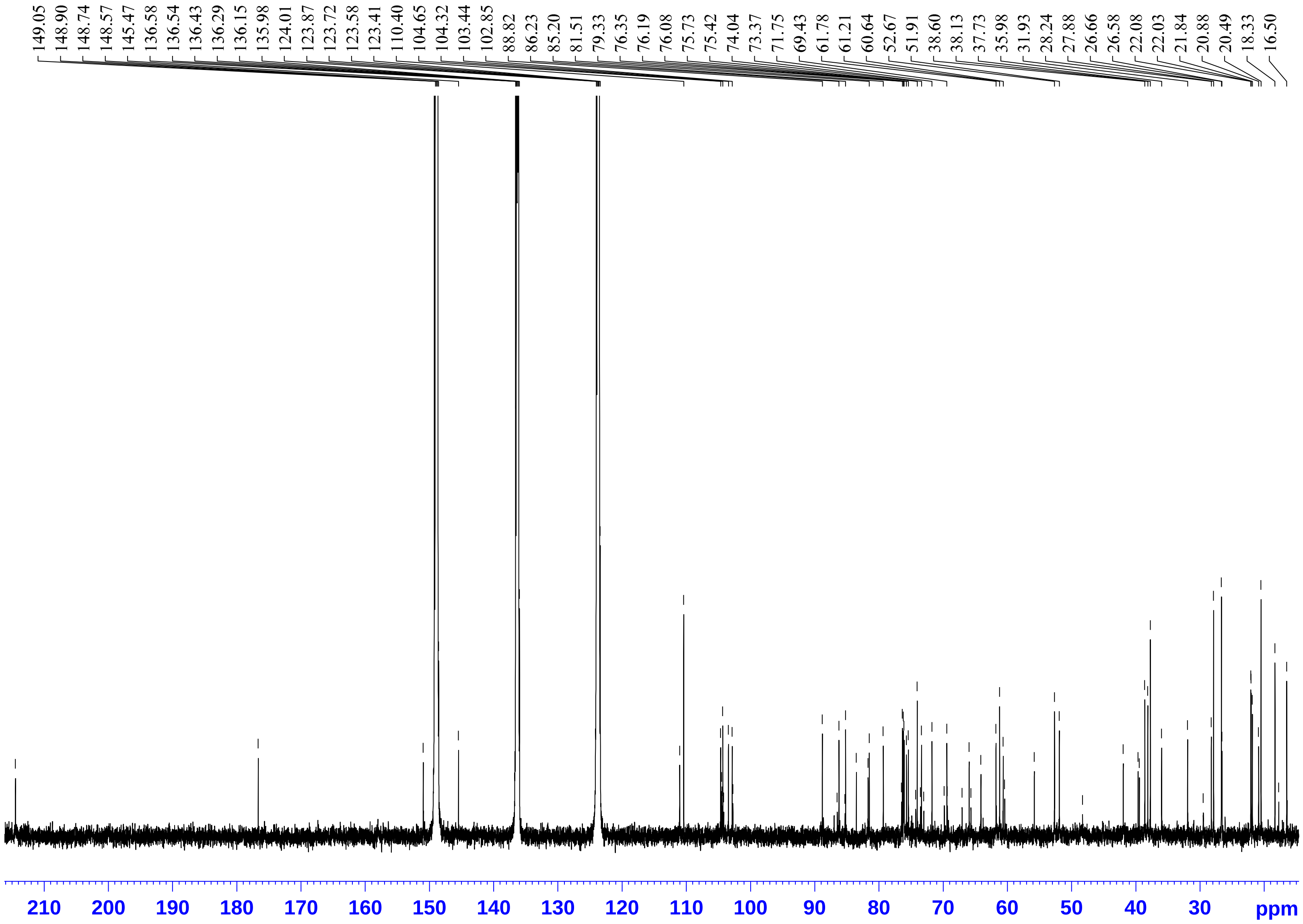

Fig. 58. The ^{13}C NMR (176.03 MHz) spectrum of quadrangularisoside D_2 (**10**) in C_5D_5N/D_2O (4/1)

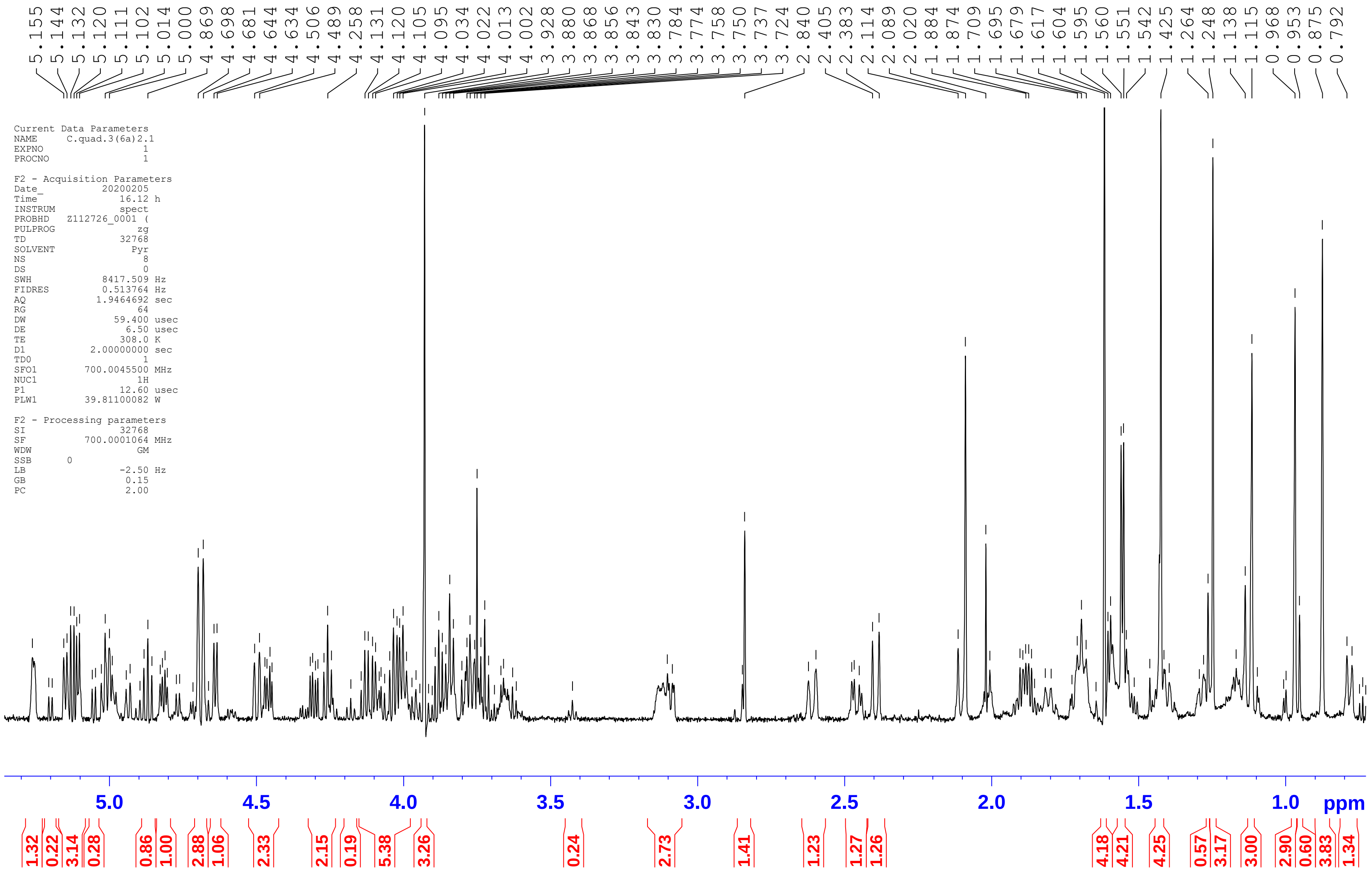

Fig. 59. The ^{1}H NMR (700.00 MHz) spectrum of quadrangularisoside D_2 (**10**) in C_5D_5N/D_2O (4/1)

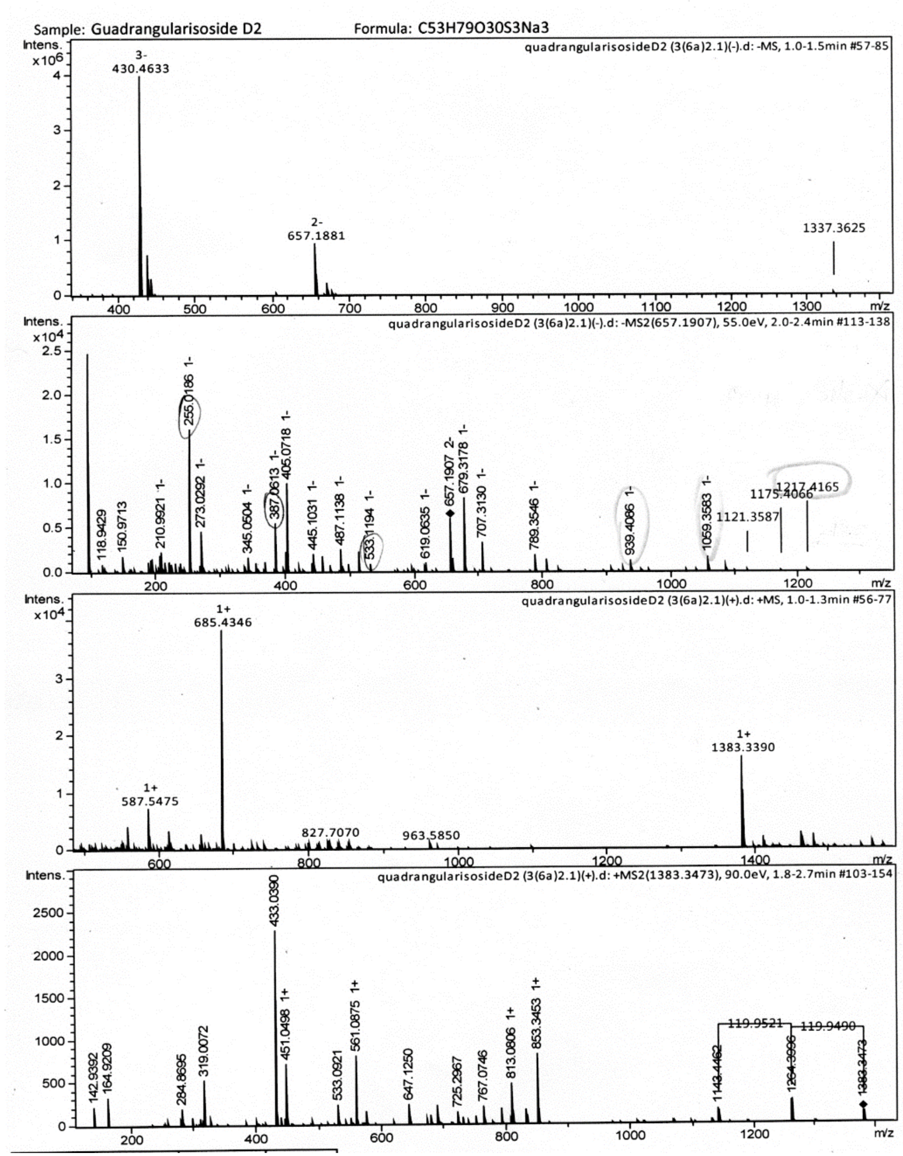

Fig. 60. HR-ESI-MS and ESI-MS/MS spectra of quadrangularisoside D_2 (**10**)

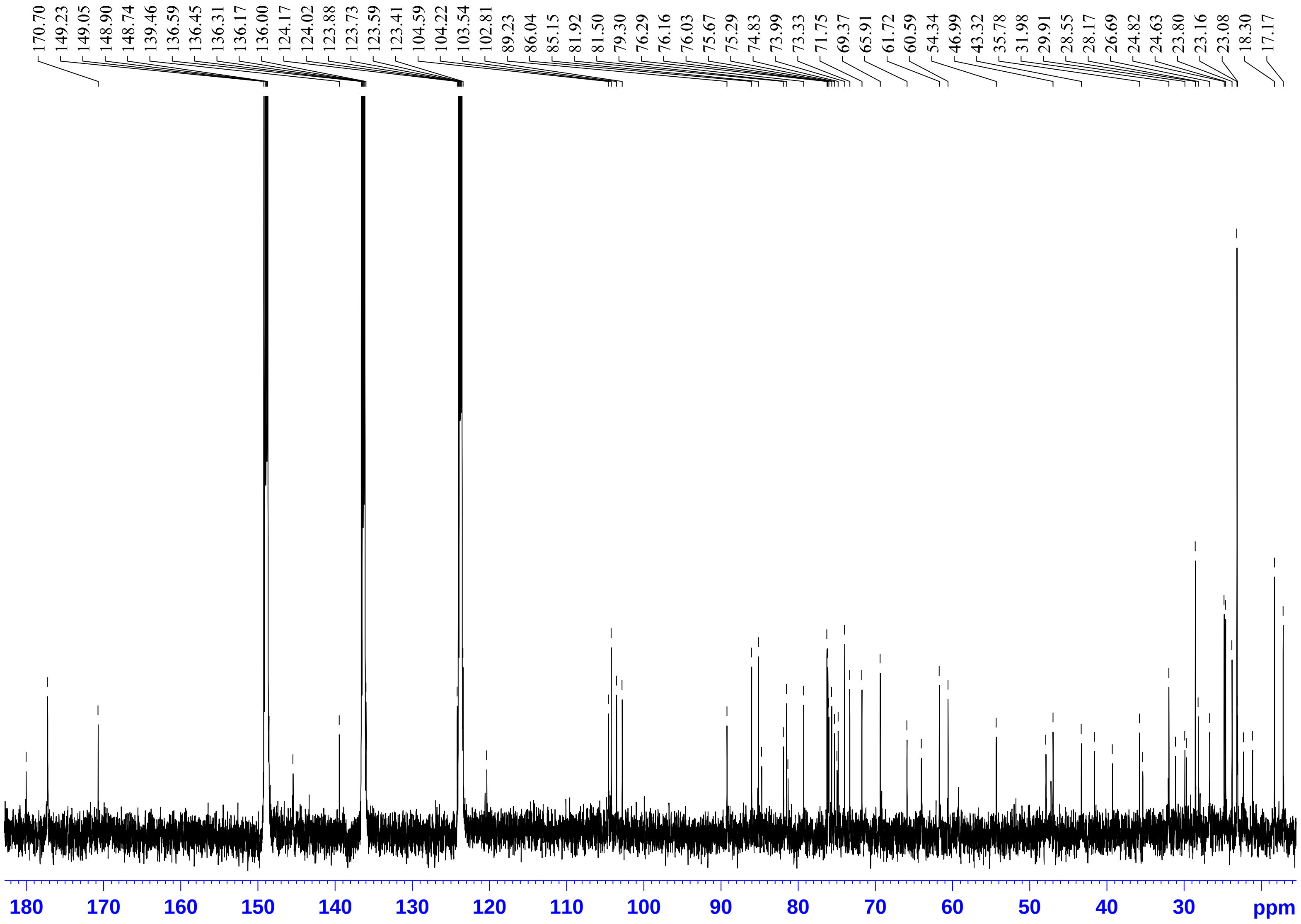

Fig. 61. The ^{13}C NMR (176.03 MHz) spectrum of quadrangularisoside D$_3$ (**11**) in C_5D_5N/D_2O (4/1)

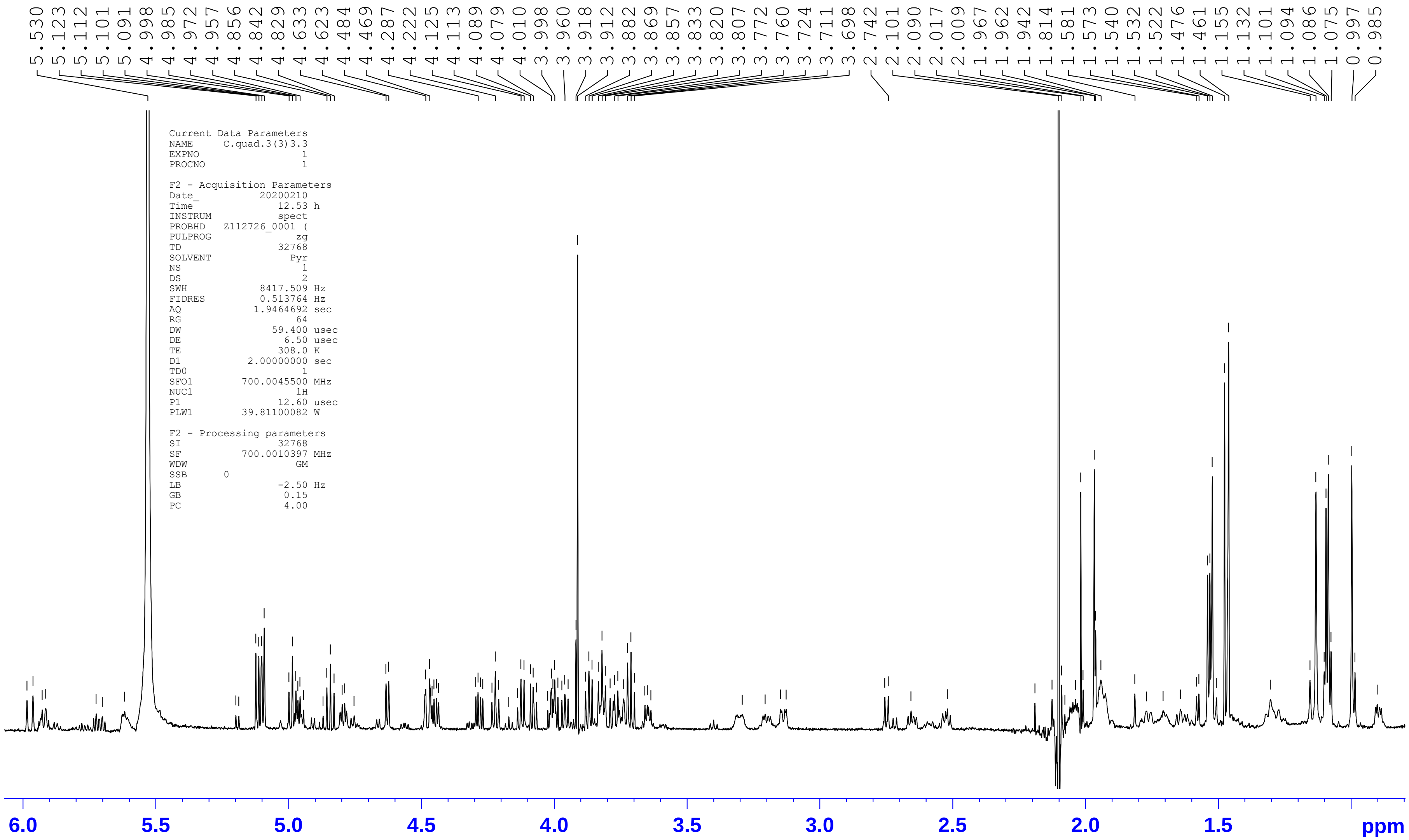

Fig. 62. The ^{1}H NMR (700.00 MHz) spectrum of quadrangularisoside D_3 (**11**) in C_5D_5N/D_2O (4/1)

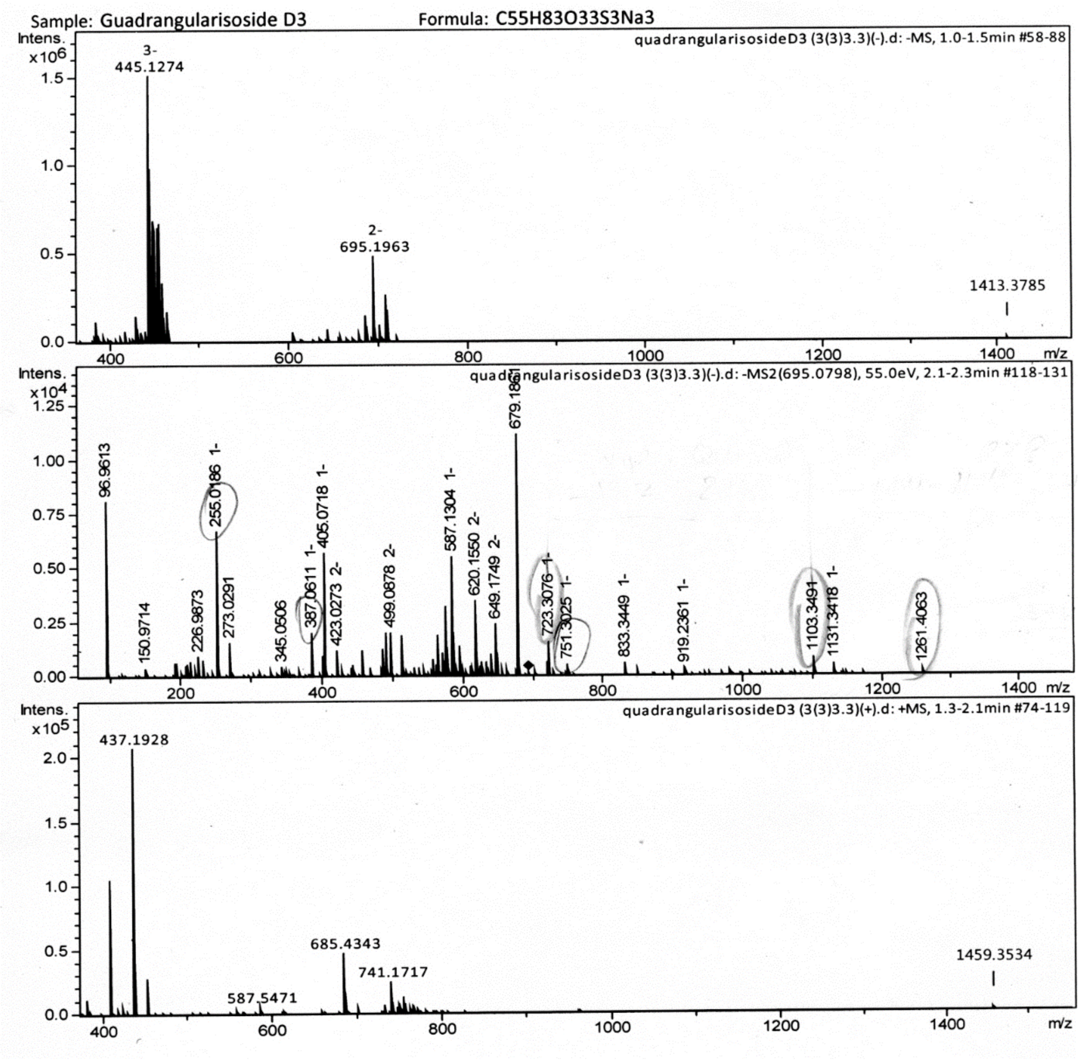

Fig. 63. HR-ESI-MS and ESI-MS/MS spectra of quadrangularisoside D_3 (**11**)

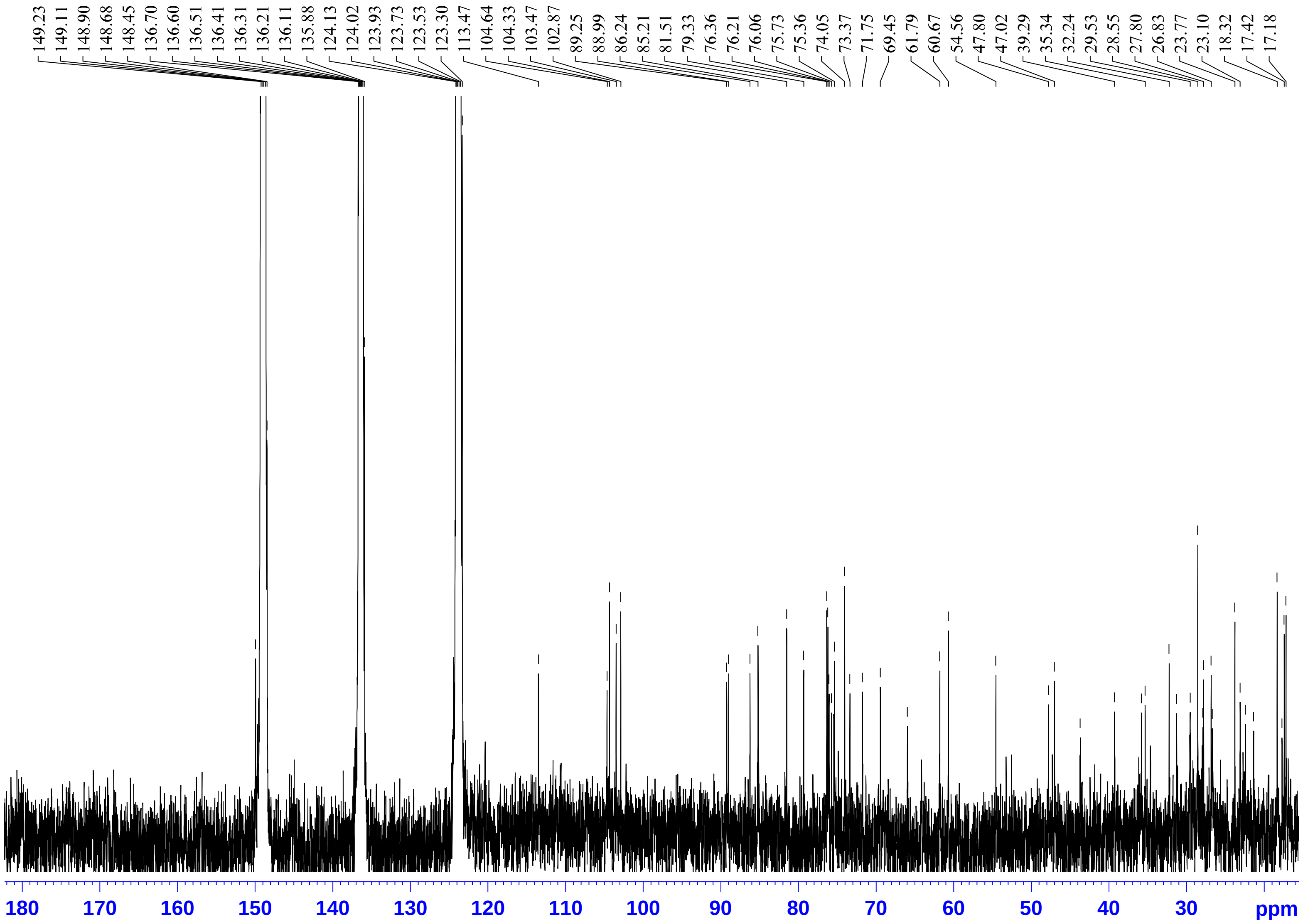

Fig. 64. The ^{13}C NMR (176.03 MHz) spectrum of quadrangularisoside D_4 (**12**) in C_5D_5N/D_2O (4/1)

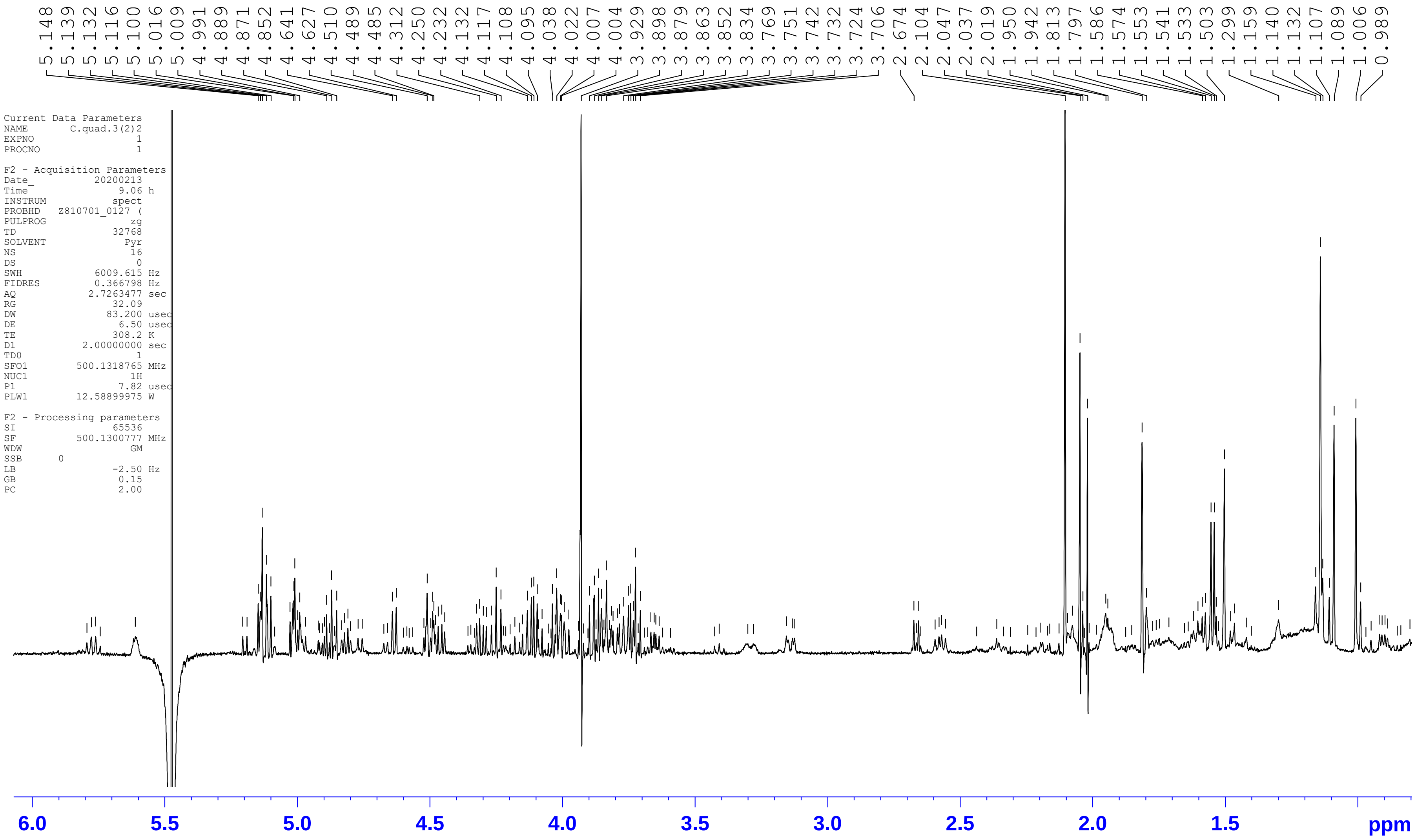

Fig. 65. The ^{1}H NMR (700.00 MHz) spectrum of quadrangularisoside D4 (**12**) in C_5D_5N/D_2O (4/1)

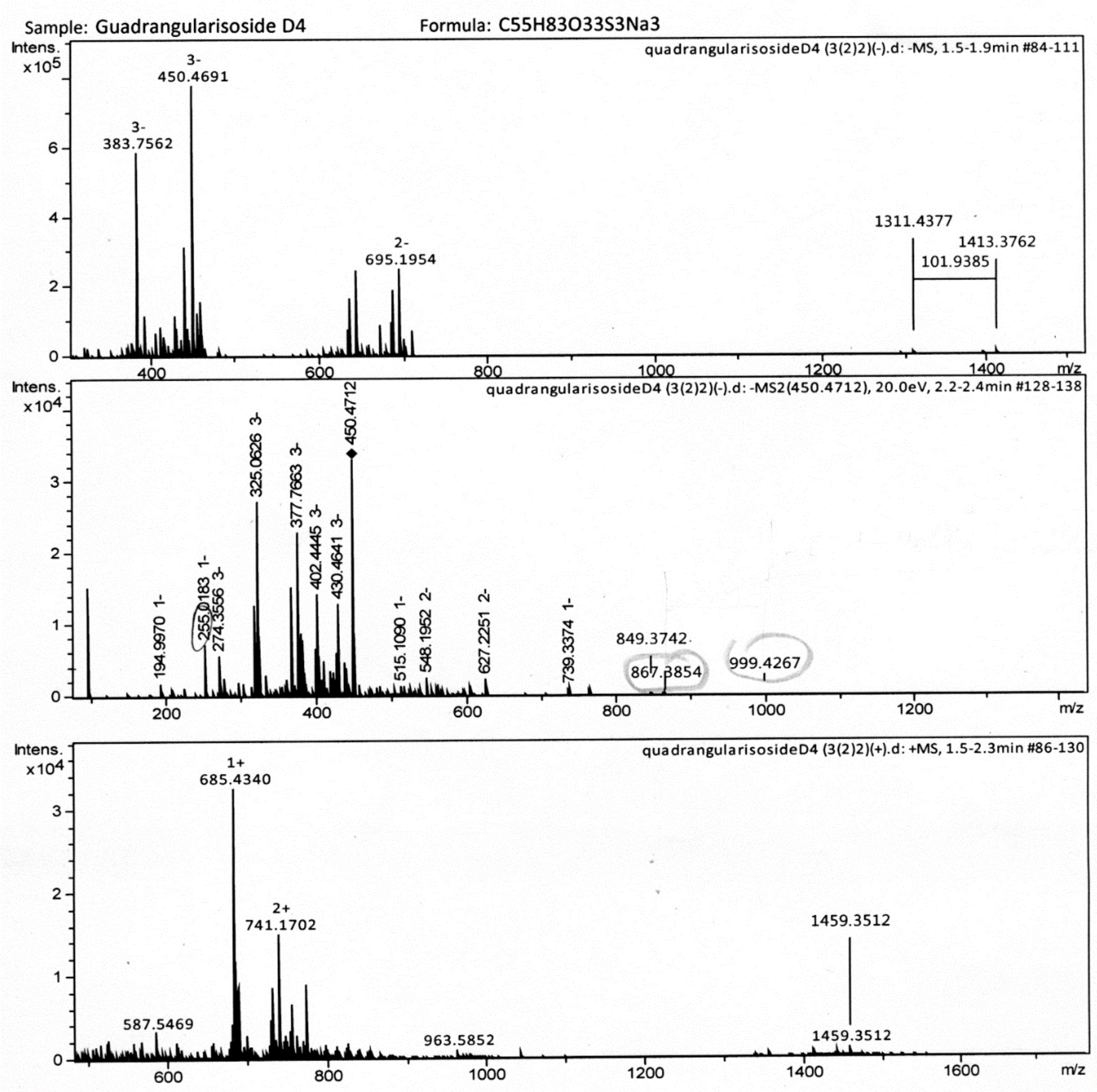

Fig. 66. HR-ESI-MS and ESI-MS/MS spectra of quadrangularisoside D$_4$ (**12**)

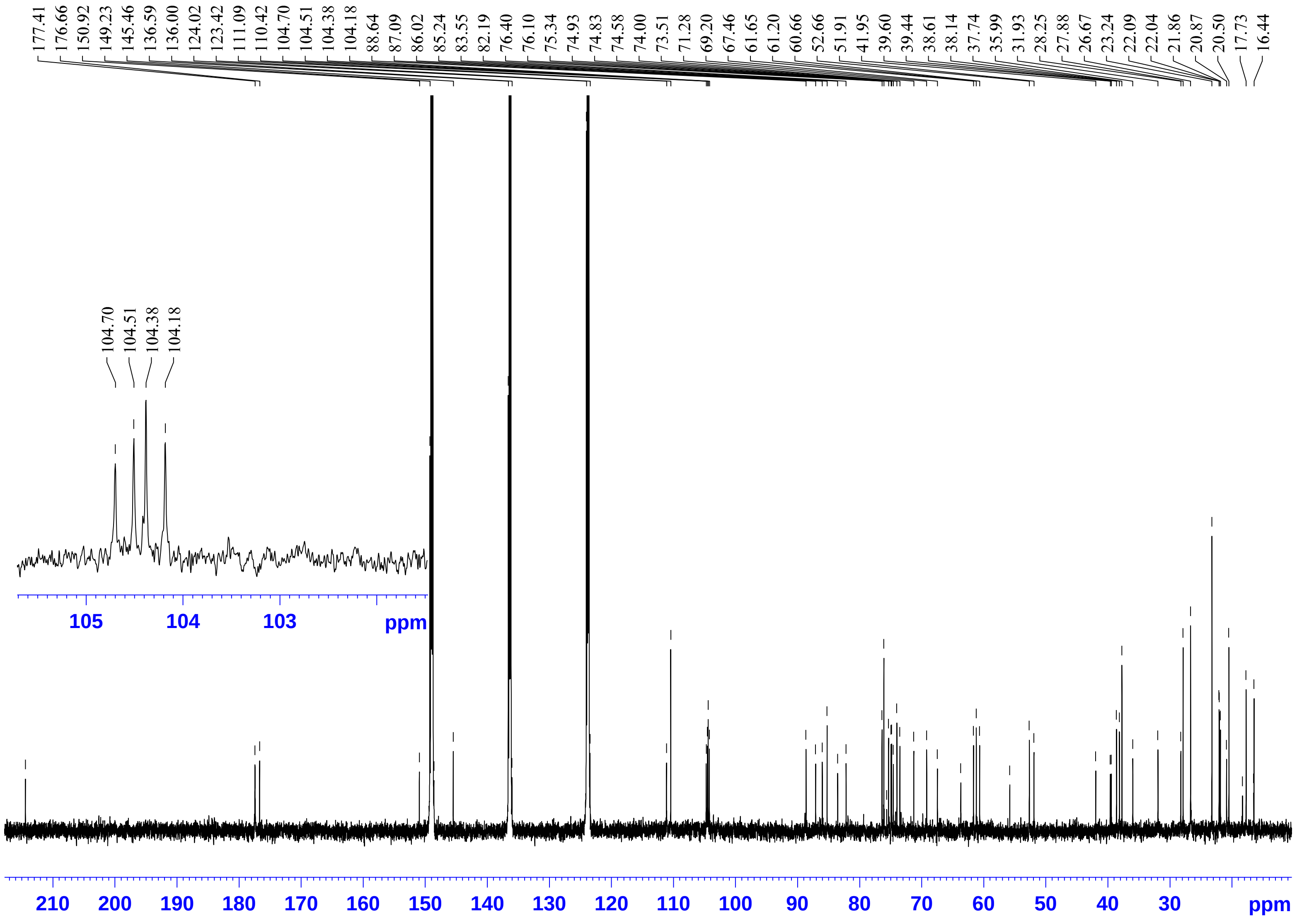

Fig. 67. The ^{13}C NMR (176.03 MHz) spectrum of quadrangularisoside E (**13**) in C_5D_5N/D_2O (4/1)

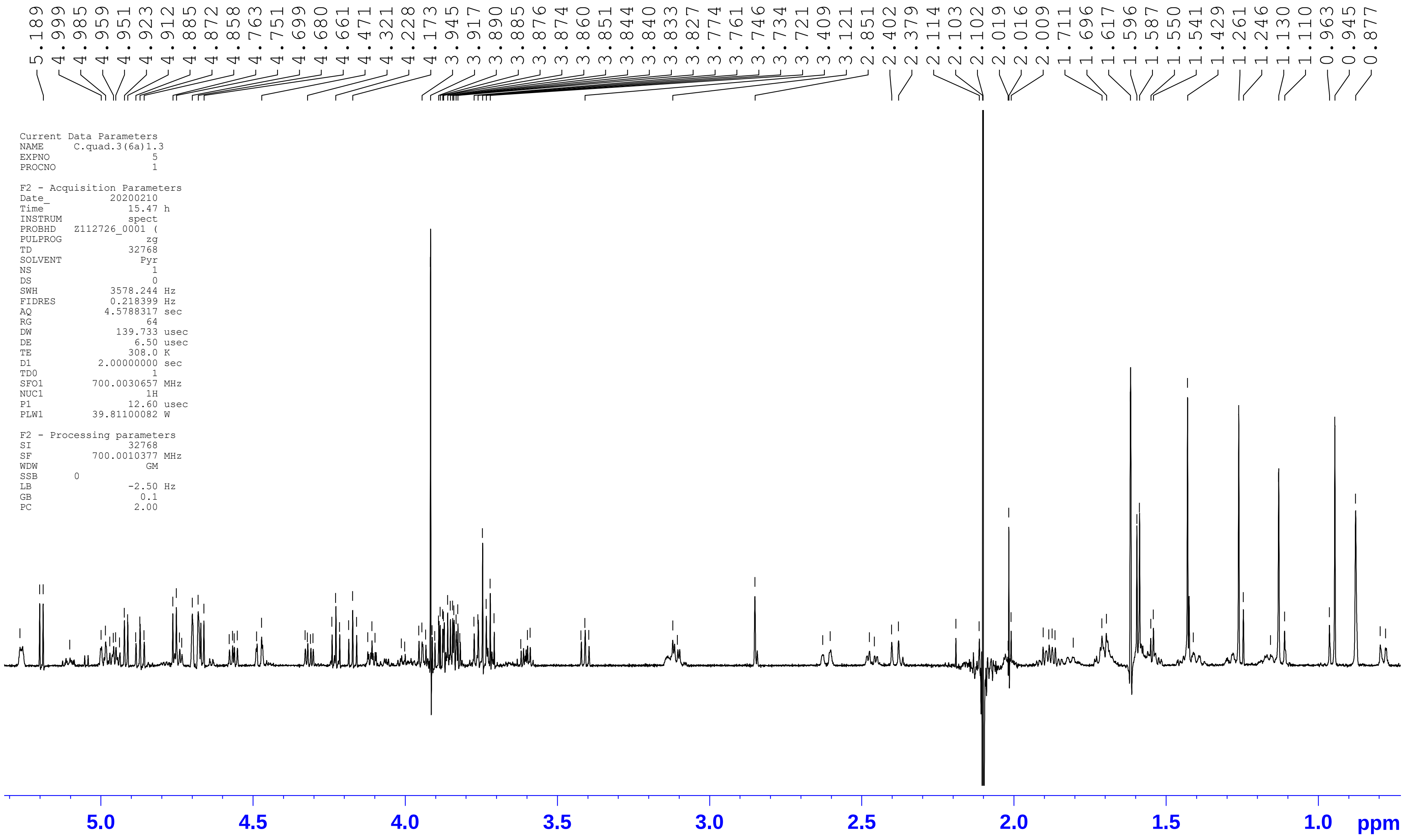

Fig. 68. The ^{1}H NMR (700.00 MHz) spectrum of quadrangularisoside E (**13**) in C_5D_5N/D_2O (4/1)

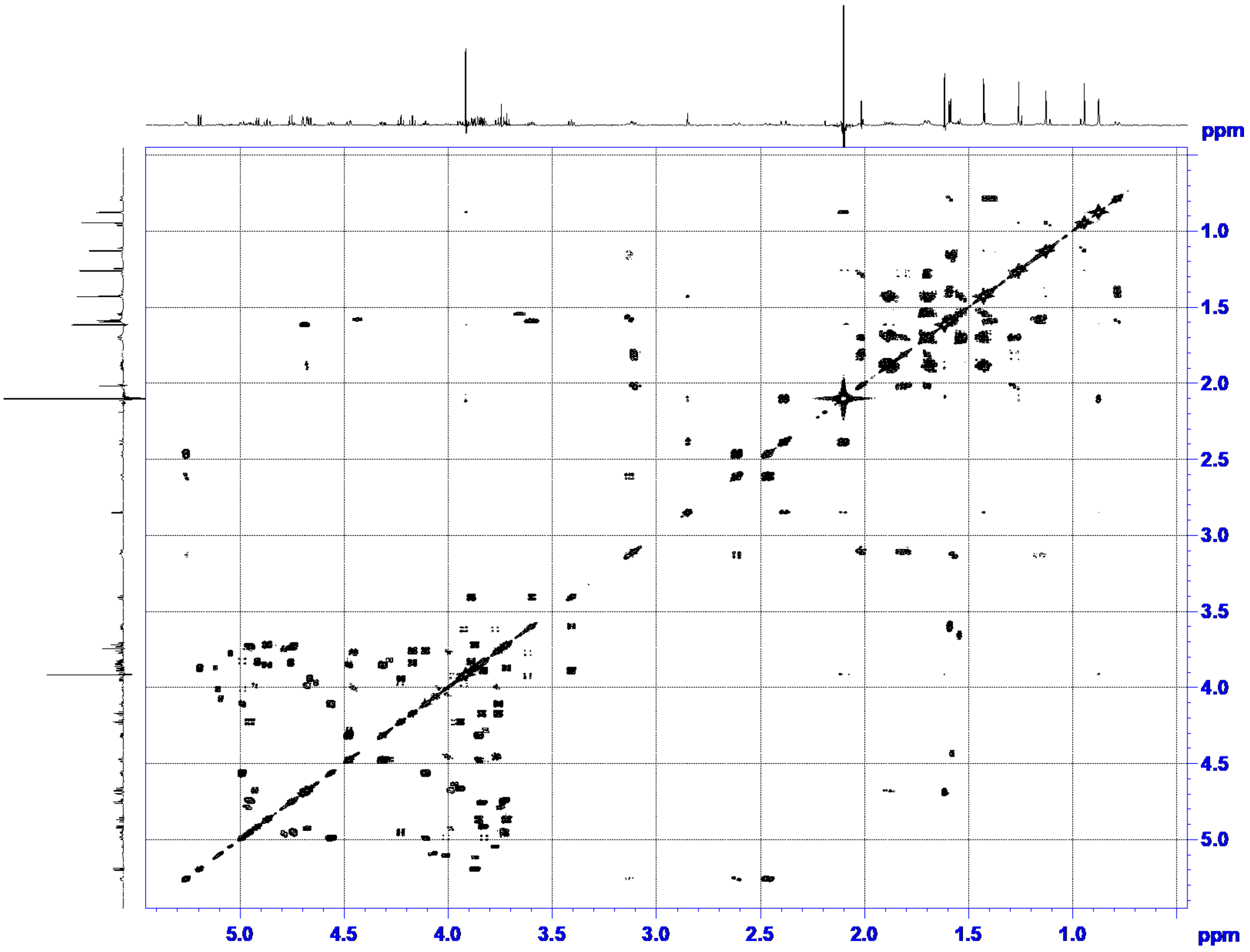

Fig. 69. The COSY (700.00 MHz) spectrum of quadrangularisoside E (**13**) in C_5D_5N/D_2O (4/1)

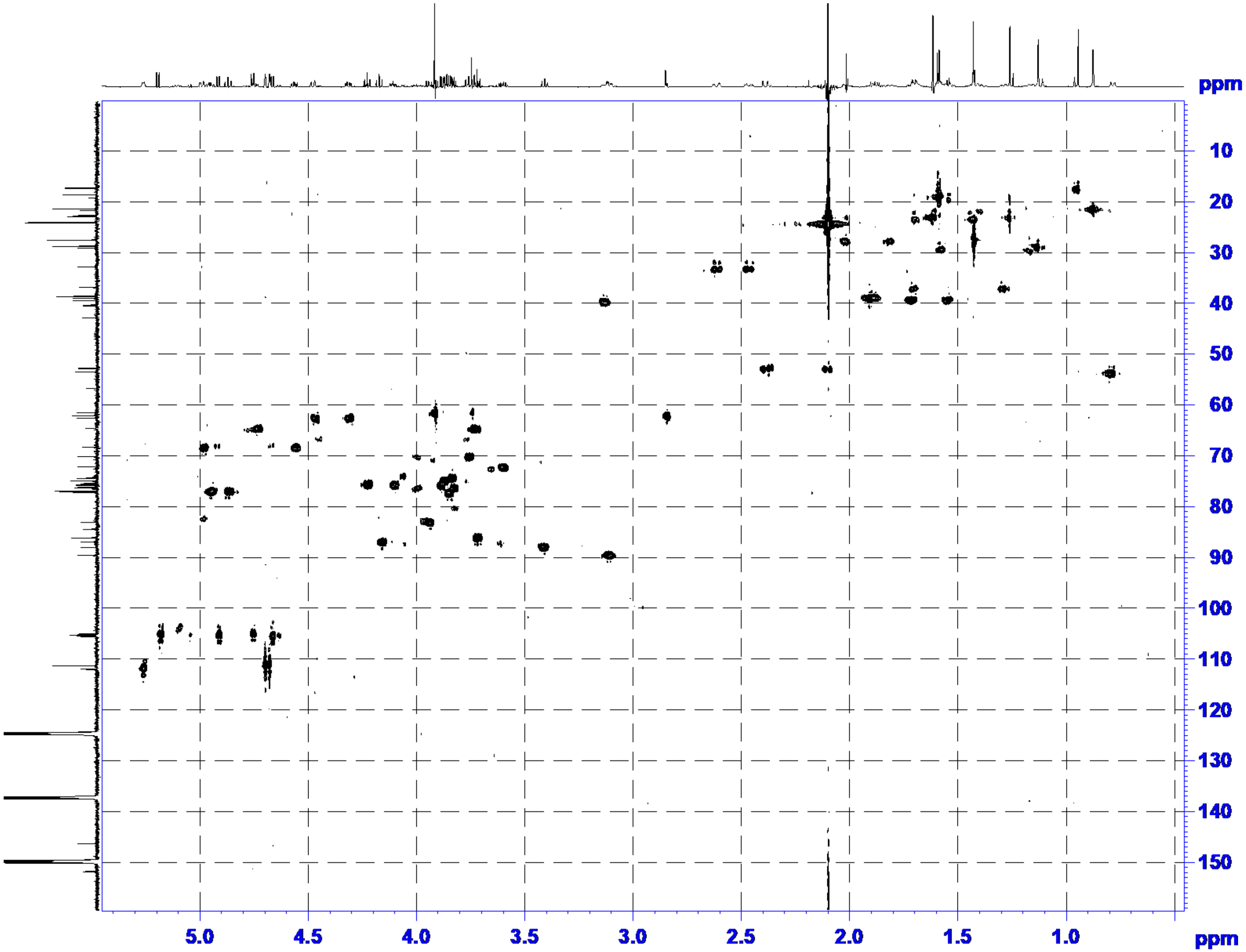

Fig. 70. The HSQC (700.00 MHz) spectrum of quadrangularisoside E (**13**) in C_5D_5N/D_2O (4/1)

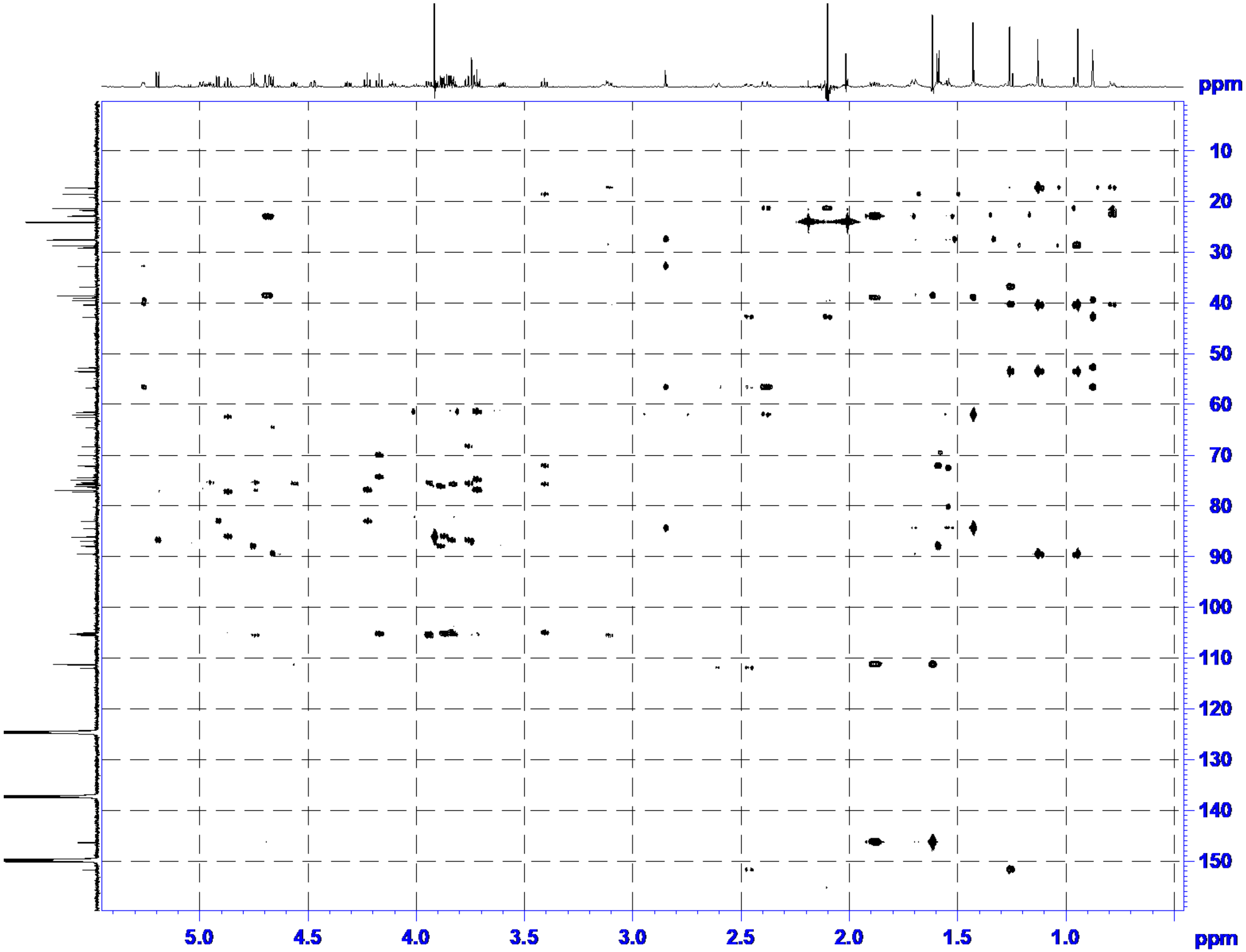

Fig. 71. The HMBC (700.00 MHz) spectrum of quadrangularisoside E (**13**) in C_5D_5N/D_2O (4/1)

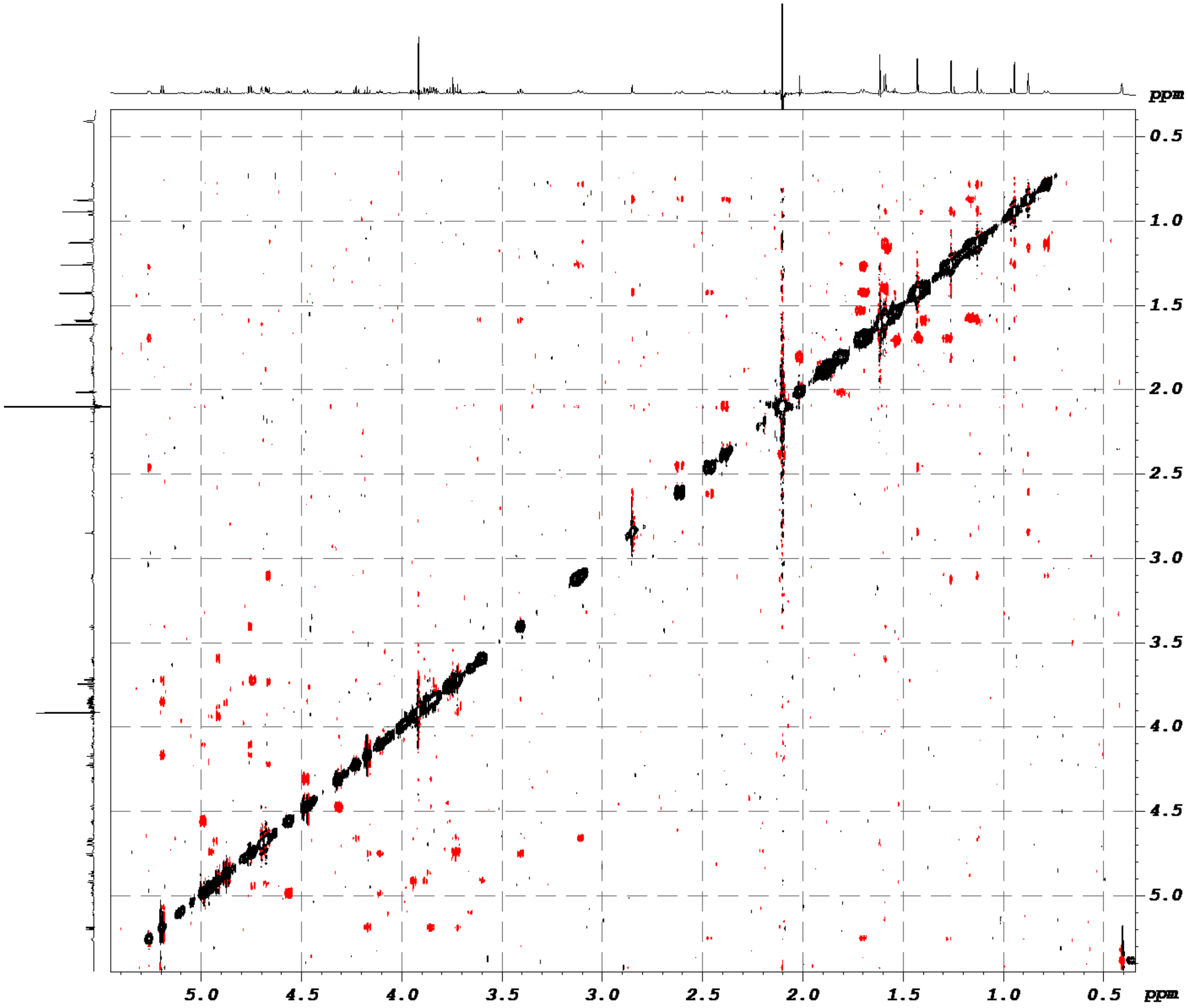

Fig. 72. The ROESY (700.00 MHz) spectrum of quadrangularisoside E (**13**) in C_5D_5N/D_2O (4/1)

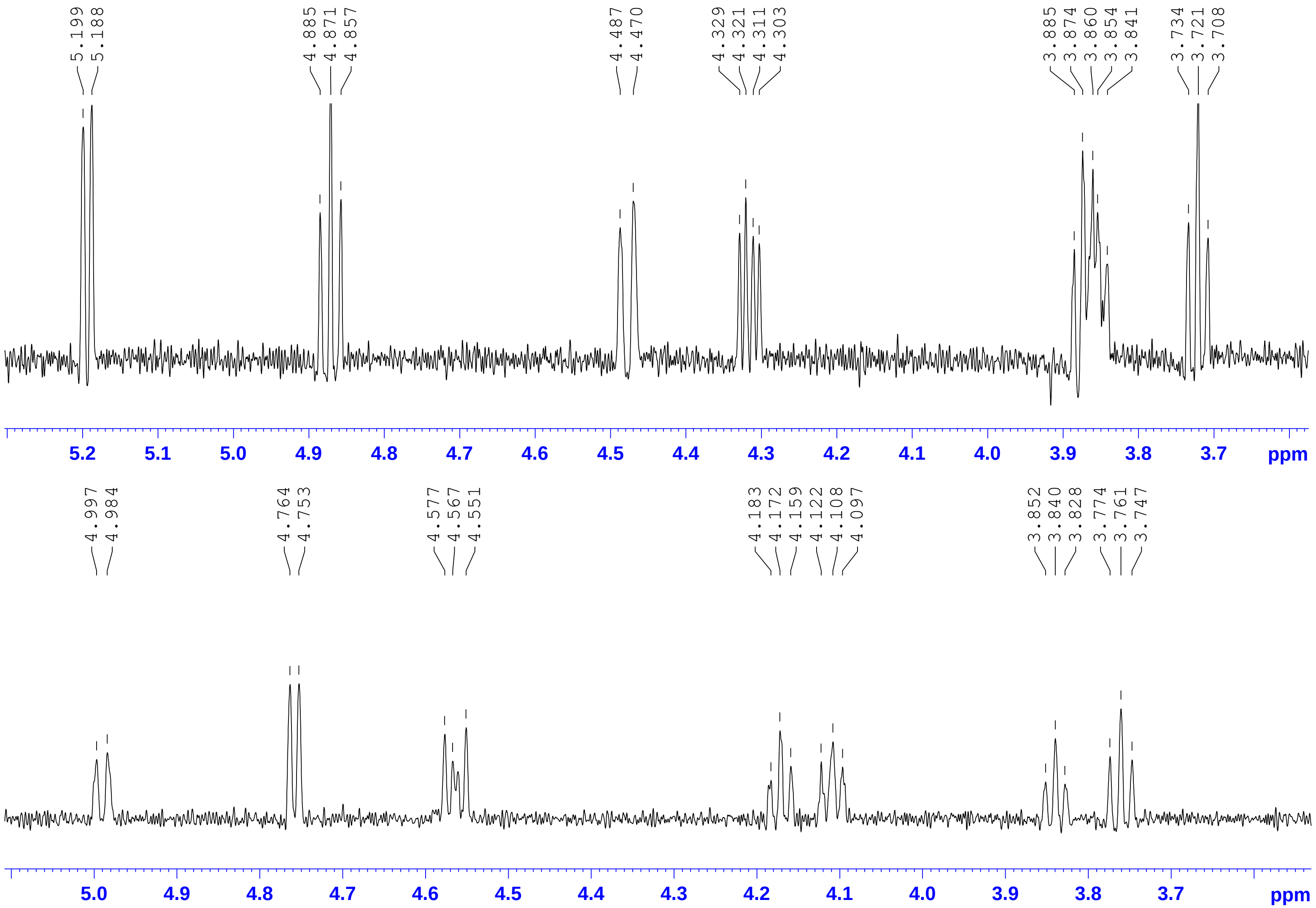

Fig. 73. 1D TOCSY (700.00 MHz) spectra of quadrangularisoside E (**13**) in C_5D_5N/D_2O (4/1)

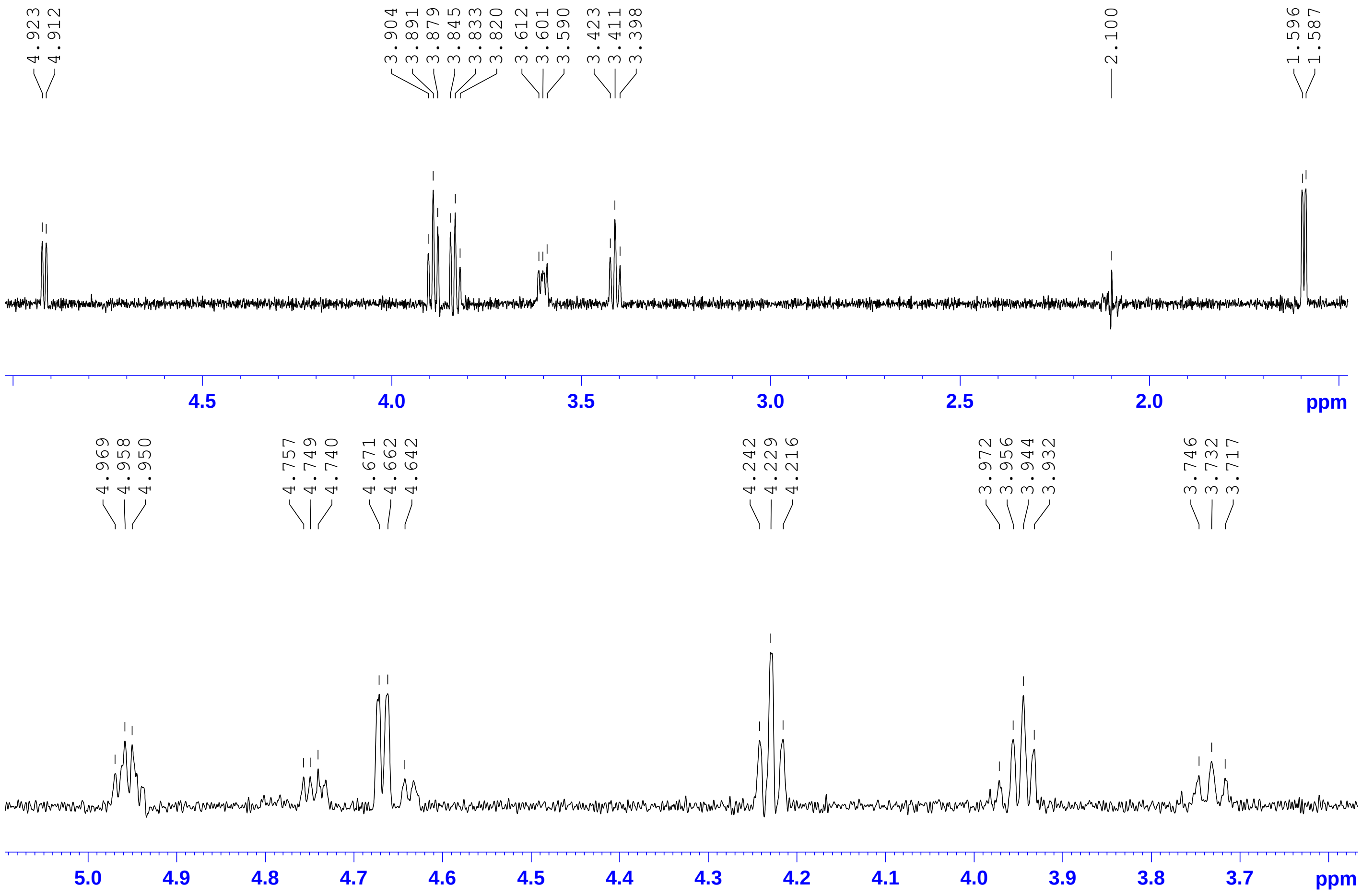

Fig. 74. 1D TOCSY (700.00 MHz) spectra of quadrangularisoside E (**14**) in C_5D_5N/D_2O (4/1)
